# Shifts in microbial communities driven by the replacement of seagrasses by benthic macroalgae may exacerbate heatwave effects in eutrophicated coastal lagoons

**DOI:** 10.1128/msystems.00291-26

**Published:** 2026-07-20

**Authors:** Esther Rubio-Portillo, Francesc Rosselló, Borja Aldeguer-Riquelme, Irene García, Fernando Santos, Raquel Gil Minguez, María Dolores Belando, Jaime Bernardeau-Esteller, Josefa Antón

**Affiliations:** 1 Department of Physiology, Genetics, and Microbiology, University of Alicante 16718, San Vicente del Raspeig, Spain; 2 Mathematics and Computer Science Department, University of the Balearic Islands 16745, Palma, Spain; 3 Balearic Islands Health Research Institute (IdISBa), Palma, Spain; 4 Department of Biomedicine and Dentistry, Faculty of Biomedical Sciences and Sports, Universidad Europea de Andalucía 738121, Málaga, Spain; 5 Applied Biomedicine Group (Alicante Institute for Health and Biomedical Research, ISABIAL), Alicante, Spain; 6 Seagrass Ecology Group (GEAM), IEO, CSIC, Centro Oceanográfico de Murcia, Murcia, Spain; 7 Multidisciplinary Institute of Environmental Studies Ramon Margalef, Alicante, Spain; Stellenbosch University, Stellenbosch, South Africa

**Keywords:** marine heatwaves, *Caulerpa*, *Cymodocea*, compositional data analysis, sediment microbiome, Mar Menor, coastal lagoon

## Abstract

Climate change is expected to increase both the frequency and intensity of marine heatwaves, prolonging periods of extreme sea surface temperatures. These events can disrupt stratification, reduce oxygen availability, and alter nutrient cycling, ultimately reshaping marine community structure and function. Impacts are likely to be particularly severe in coastal lagoons, which could compromise their biodiversity and ecosystem services. In this study, we simulated a marine heatwave under controlled mesocosm conditions to investigate its influence on microbial communities in sediments colonized by the native seagrass *Cymodocea nodosa* and the opportunistic seaweed *Caulerpa prolifera* in the eutrophicated Mar Menor coastal lagoon. Community shifts and ecologically relevant taxa were assessed by using 16S rRNA gene metabarcoding and compositional data analysis, using complementary statistical approaches combining log ratio analysis and the glmnet algorithm. Our results show that sediments colonized by different macrophyte species harbor distinct microbial assemblages with different functional capacities. While sediments associated with the invasive macroalga *Caulerpa prolifera* were enriched in sulfate-reducing bacteria (mostly *Desulfobacterota* and *Desulfosarcinaceae*), *Cymodocea nodosa* colonized sediments showed higher abundances of sulfur-oxidizing taxa such as *Thiotrichaceae*. During marine heatwaves, *Cymodocea nodosa* sediments exhibited an increase in sulfate reducers coupled with a decline in sulfur oxidizers that favored sulfide accumulation. These findings suggest that marine heatwaves may alter sediment microbial sulfur cycling in vegetated coastal systems, with potential implications for ecosystem resilience. Our results also highlight the importance of considering microbial processes when evaluating the responses of coastal lagoons to increasingly frequent and intense marine heatwaves.

**IMPORTANCE:**

Coastal lagoons are among the ecosystems most vulnerable to thermal anomalies; however, the responses of sediment-associated microbial communities remain poorly understood. Here, we assess the impact of simulated marine heatwaves on sediment microbiota associated with the seagrass *Cymodocea nodosa* and the macroalga *Caulerpa prolifera* under controlled laboratory conditions. Heatwave exposure led to the accumulation of sulfur compounds and pronounced shifts in microbial community composition, indicating altered biogeochemical functioning. These microbial responses suggest that marine heatwaves could potentially contribute to conditions that have been associated with dystrophic events in eutrophicated lagoons. Our findings provide evidence that future marine heatwaves could alter the microbial composition of coastal lagoon sediments and highlight the urgent need to incorporate microbial processes into ecosystem monitoring and management frameworks.

## INTRODUCTION

Anthropogenic climate change has emerged as a significant environmental driver, profoundly impacting marine biodiversity and ecosystem functioning, often in synergy with other human-induced stressors (1). Beyond gradual ocean warming, human activities have markedly increased the frequency and intensity of extreme climatic events, such as marine heatwaves (2). These extreme episodes are characterized by abnormally high seawater temperatures that persist for days to months. More specifically, an anomalously warm event can be considered a marine heatwave (MHW) if it lasts for 5 or more days, with temperatures warmer than the 90th percentile based on a 30-year historical baseline period (3). Over the past 20 years, MHWs have doubled in frequency globally and have become longer, more intense, and more widespread (4).

The Mediterranean Sea is among the marine regions most vulnerable to climate change. In recent decades, Mediterranean Sea surface temperatures have increased substantially, at rates ranging from +0.29°C to +0.44°C per decade, surpassing the global average (5). This accelerated warming is expected to persist throughout the 21st century, with surface temperatures potentially rising by up to 3.8°C under high-emission scenarios (6). Consequently, the frequency, duration, and intensity of MHWs are projected to rise, resulting in at least one prolonged event occurring annually by 2100 (6). These changes pose an escalating threat to marine biodiversity, a trend already reflected in unprecedented climate-induced mass mortality events observed in recent decades (7).

Coastal ecosystems are among the most productive habitats on Earth, providing essential social and economic benefits; however, they are particularly vulnerable to environmental changes (8). One such habitat is coastal lagoons, shallow water bodies separated from the ocean by natural barriers and connected through one or more restricted inlets (9). Their small water volumes and slow renewal rates make them particularly susceptible to the impacts of human activities. Additionally, the consequences of climate change, such as rising temperatures and altered hydrological cycles, are expected to amplify these pressures (10). Coastal lagoons are therefore regarded as some of the most fragile marine environments in the context of global warming and its cascading effects. Understanding the combined influence of climatic and anthropogenic drivers on these ecosystems is crucial for developing effective management strategies and ensuring their preservation and long-term sustainability.

Prokaryotes are essential components of coastal lagoons, playing a crucial role in the remineralization process and driving the recycling of nutrients for sustaining primary production (11, 12). Their activity is particularly important in organic-rich sediments, where oxygen depletion creates anoxic conditions that lead to the microbial production of hydrogen sulfide, which is highly toxic for many aquatic organisms. Certain microbial groups, such as sulfur-oxidizing bacteria, regulate sulfide levels by mediating sulfur cycling, thereby acting as a buffer system against sulfide toxicity under dystrophic conditions (13).

Coastal lagoons are frequently colonized by marine macrophytes, including seagrasses and benthic macroalgae, whose presence strongly influences sediment biogeochemistry and microbial community structure. Plant-driven inputs of organic matter, root or rhizoid oxygen release, and the production of exudates strongly shape microbial community composition and metabolic activity, often resulting in a tight coupling between sulfate-reducing and sulfur-oxidizing microorganisms in the rhizosphere (14, 15). In natural seagrass meadows under warmer temperature conditions, sediment microbial communities are often characterized by relatively high abundances of putative sulfate-reducing bacteria (16). Thus, these vegetation-associated sediment microbiomes play a central role not only in sulfur cycling but also in regulating carbon degradation and burial processes, thereby contributing to the capacity of seagrass ecosystems to act as long-term carbon sinks (17). Therefore, changes due to the increase in seawater temperature in the composition of sediment microbial communities associated with macrophytes could profoundly impact the geochemistry and overall functioning of coastal lagoon ecosystems. Experimental evidence further indicates that elevated temperatures and simulated marine heatwaves can alter seagrass-associated sediment microbiomes, with potential consequences for plant performance due to changes in microbial metabolism, sulfur cycling, and the balance between beneficial and pathogenic microorganisms (18).

The Mar Menor, located in southeastern Spain, is the largest hypersaline coastal lagoon in Europe and serves as a paradigmatic example of a highly valuable yet heavily impacted coastal marine ecosystem. Anthropogenic pressures have promoted a transition from an oligotrophic ecosystem, primarily dominated by the seagrass *Cymodocea nodosa*, to a eutrophic lagoon, where the high nutrient availability favored the expansion of the seaweed *Caulerpa prolifera* (19). As a result, most of the bottom of the lagoon is occupied by mixed meadows of both species (20). In 2016, a prolonged phytoplankton bloom disrupted the water column conditions in the lagoon, which led to a massive mortality event affecting benthic vegetation (21). Approximately 85% of the *Cy. nodosa* and *Ca. prolifera* meadows were lost, becoming restricted to the shallower areas of the lagoon (<2 m), while deeper zones were left bare (22). From 2017 onwards, following an improvement in oceanographic conditions, a recolonization process by both macrophytes began. However, the latest available data indicate that this new expansion phase has been limited for *Cy. nodosa*, which is currently only present in shallow areas (<4 m). By contrast, the seaweed has been able to recolonize all the lagoon bottoms, and it is now the dominant macrophyte also in deeper areas (19).

In a previous study, we provided a detailed characterization of the microbial community differences in sediments colonized by *Ca. prolifera* and *Cy. nodosa*, highlighting their ecological significance for the health of the lagoon. Notably, sediments colonized by *Ca. prolifera* exhibited a higher relative abundance of putative sulfate-reducing bacteria and, accordingly, a lower redox potential compared to *Cy. nodosa*. Thus, the expansion of seaweed can promote the proliferation of sulfide-producing microorganisms that negatively impact the entire ecosystem (23). These findings are particularly relevant, given their implications for the current ecological status of the lagoon, especially considering the shifts in vegetation following the 2016 dystrophic crisis and the subsequent predominance of *Ca. prolifera*. Thus, the occurrence of heatwaves in the lagoon may affect not only macrophyte and marine vegetation communities but also the sediment-associated microbial communities. Considering the distinct functional profiles of the communities associated with the two dominant macrophyte species, the changes induced by such extreme events in this compartment may be critical in determining the ecological state of the lagoon (e.g., by generating more reducing conditions and increasing the concentration of sulfides).

In this work, we hypothesized that heatwave stress would induce macrophyte-dependent changes in microbial community sediment structure, with *Cy. nodosa* sediments being more susceptible to community shifts than those associated with *Ca. prolifera*. To test this hypothesis, we evaluate changes in microbial communities within sediments colonized by *Ca. prolifera* and *Cy. nodosa* under heatwave stress simulated by mesocosm experiments. To achieve our objective, we analyzed 16S rRNA gene amplicon data using compositional data analysis (CoDA), a statistical framework appropriate for compositional microbiome data sets (24), in which observations are expressed as proportions and subject to constant-sum constraints. Moreover, we applied several CoDA-based operational taxonomic units (OTU) selection methods and implemented a filtering strategy to remove low-abundance OTUs and focus on biologically meaningful community shifts.

## MATERIALS AND METHODS

### Sampling and experimental setup

**Fig 1:**
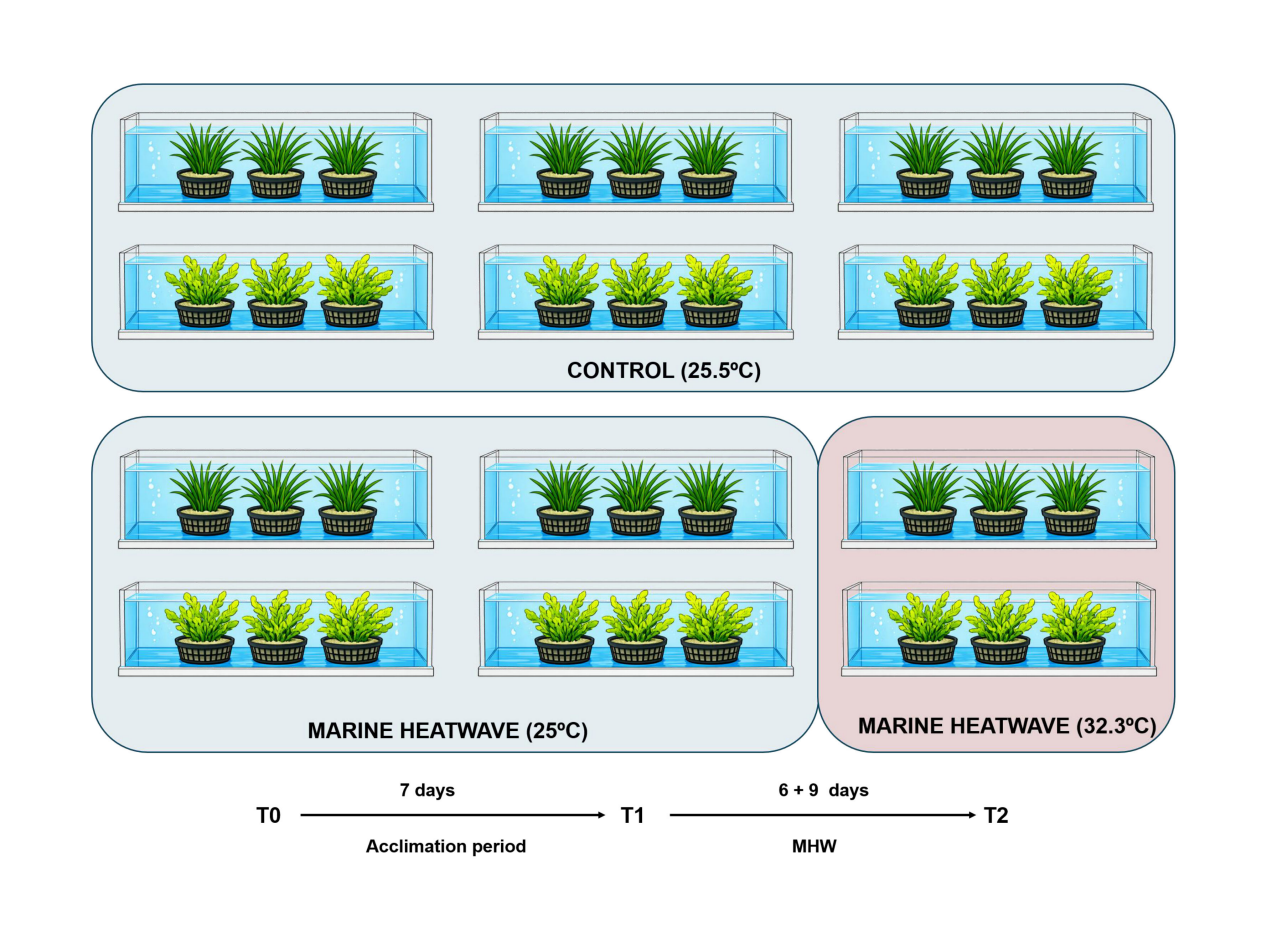
Experimental setup. Intact sediment cores associated with *Cy. nodosa* and *Ca. prolifera* were placed in independent aquaria, each containing three pots of a single species. For each macrophyte, six aquaria were used: three maintained under control temperature conditions (25.5°C) and three exposed to a simulated marine heatwave. After a 6-day acclimation period, the temperature in heatwave aquaria was gradually increased by 0.3°C day⁻¹ for 6 days until reaching 32.3°C and then maintained for 9 additional days. Control aquaria were kept at a constant temperature throughout the experiment.

Intact meadow sediment cores associated with both macrophyte species were collected manually by autonomous diving. These cores were extracted in June 2021 from natural meadows located in the northern Mar Menor (La Encanizada, 37°47'22.1" N, 0°45'56.5" W; 1.5 m depth). The cores were placed in plastic pots to maintain their structural integrity and transported filled with seawater in refrigerated coolers in darkness to the mesocosm system at the Centro Oceanográfico de Murcia (IEO-CSIC). The mesocosm system, previously validated for the cultivation of marine macrophytes (25), consisted of 12 independent 250 L PVC aquaria integrated into a seawater circulation circuit with a 500 L reserve tank, in which water quality, temperature, light, salinity, pH, and nutrients were controlled. Each aquarium contained three pots of a single species, with six aquaria per vegetation type (Fig. 1, where T0 represents the time when samples arrived at the laboratory and were placed in the aquaria). Irradiation was carried out with a 400 W metal halide lamp (Aqua Medic Aqualight-400). Water temperature was regulated by an automated control system (±0.1°C), combining heating and cooling units coupled to temperature sensors in each aquarium. pH was continuously monitored using pH electrodes (Aqua Medic AT-Control), and salinity was measured using a conductivity meter (WTW Cond 197i). Prior to the heatwave, pots were acclimated for 1 week under environmental conditions representative of the sampling site: salinity 40 psu, temperature 25.5°C, irradiance 300 μmol m⁻² s⁻¹, and a 12 h light:12 h dark photoperiod.

After acclimation (T1 in Fig. 1), temperature was progressively increased in three aquaria containing *Cy. nodosa* and three containing *Ca. prolifera* (randomly selected) at a rate of 0.3°C per day, reaching 32.3°C, which is the temperature observed in the lagoon during a MHW (Bernardeau-Esteller, unpublished data). Samples were collected after 6 days (T2 in Fig. 1). The temperature was maintained at 25.5°C in the control aquaria. To minimize position effects, pots were randomly rearranged every 2–3 days.

### Physicochemical measurements

Redox potential and sulfide concentration in the sediments were determined in each pot from every aquarium at the beginning (T0) and at the end (T2) of the experiment. For redox measurements, readings were taken 5 cm below the sediment-water interface using a Crison platinum electrode connected to a portable pH meter (Crison Model 507). The electrode was calibrated using a standard redox solution (Crison, 468 mV at 25°C), and all measurements were referenced to the standard hydrogen electrode (+207 mV) (26). Total free sulfide content was measured with an ion-selective electrode (silver/sulfide combination electrode 9616 BNWP) following the method described by Wildish et al. (27).

### Microbial counts, DNA extraction, 16S rRNA gene PCR amplification, and sequencing

For microbial analyses, three sediment samples (4.5 cm depth) were collected from three independent pots per treatment and macrophyte using 2-cm diameter cores. Sediments were homogenized in sterile 1× PBS (35 g L^−^¹ NaCl, 0.2 g L^−^¹ KCl, 1.44 g L^−^¹ Na₂HPO₄, 0.24 g L^−^¹ KH₂PO₄; pH 7.0). Cell abundance was determined by epifluorescence microscopy after DAPI staining following formaldehyde fixation (4% final concentration) (28). The remaining sediments were stored at –80°C until nucleic acid extraction.

A total of 24 sediment samples were used for DNA extraction. For each macrophyte species and experimental condition, sediments were collected from independent pots maintained within a common aquarium environment. Thus, pots represented sediment-level biological replicates within the corresponding aquarium/treatment condition. At T0 and T1, sediment samples were collected from three independent pots per macrophyte species. At T2, three independent pots were sampled for the control treatment (T2C), and three for the heatwave treatment (T2HW). Samples collected at different time points were obtained from different pots; therefore, temporal comparisons should be interpreted as differences among independent pots sampled at each time point rather than as repeated measurements of the same pots. DNA was extracted from 2 g of sediment using the RNeasy PowerSoil Total RNA Kit and the RNeasy PowerSoil DNA Elution Kit (Qiagen), following the manufacturer’s instructions. DNA concentrations were measured using Qubit assays (Invitrogen). The V3–V4 region of the 16S rRNA genes was amplified using primers 341F–805R (29) with 25 PCR cycles. Amplicons were sequenced on an Illumina MiSeq platform (2 × 250 bp) at Fundació per al Foment de la Investigació Sanitària i Biomèdica de la Comunitat Valenciana (Fisabio, Valencia, Spain).

Illumina sequences were processed using QIIME2 v 2024.2.0 (30). Reads were trimmed and quality-filtered using cutadapt (31), and sequences with Phred scores <33 were removed. OTUs were clustered *de novo* at 98.7% sequence identity (32), using the vsearch algorithm implemented in QIIME 2 (33). Taxonomy assignments were carried out using the SILVA reference database v138 (34), performing the machine learning algorithm Scikit-learn in QIIME2 (35). Chimera sequences were removed, and a frequency table was obtained using the vsearch algorithm (33) implemented in QIIME2. This OTU-based approach (instead of ASV-based) was selected to maintain consistency with previous related analyses (23) and because the study focused on broad changes in microbial community structure and dominant taxonomic groups rather than fine-scale sequence variation.

### Statistical analysis

A detailed description of all statistical analyses is included as File S1.

#### Biochemical and cellular parameters

Data on sulfide content, redox potential, and sediment cell counts in samples colonized by each species under control and heatwave conditions were analyzed using Wilcoxon signed-rank tests (36) with Benjamini-Hochberg–adjusted *P*-values (BH-adjusted *P*) for paired comparisons. Heatwave effects were further evaluated with difference-in-differences models (37) to summarize whether T0–T2 contrasts differed between the control and heatwave conditions. Because samples collected at different time points came from independent pots rather than repeated measurements of the same experimental units, these models were interpreted as exploratory interaction models rather than as formal longitudinal causal estimates. For each species, the response variable was modeled as a function of treatment, time, and their interaction. A significant treatment × time interaction was interpreted as evidence that the T0–T2 difference differed between the control and heatwave conditions.

#### Biodiversity

Shannon diversity was computed with the diversity() function (vegan, R). Species differences were tested with Wilcoxon rank-sum tests at each sampling time and in a pooled comparison across sampling times. The effect of an OTU relative-frequency threshold *P*_0_ on Shannon diversity was evaluated by retaining, for each sample, only OTUs with relative frequency ≥*P*_0_, where *P*_0_ ranged from 0.00005 to 0.05 (0.005%–5%) in increments of 0.00005. Shannon diversity was then recomputed by macrophyte species, and the proportion of OTUs retained at selected thresholds was summarized. Between-species community dissimilarity across sampling times was summarized using median Bray-Curtis distances between *Ca. prolifera* and *Cy. nodosa* samples, with statistical support assessed using a resampling-based test for differences in median distances.

#### Compositional data analysis

Compositional analyses followed standard CoDA procedures, including log ratio transformations within the Aitchison compositional data analysis framework (24, 38). Because log ratio analyses require strictly positive values, zero replacement was used as a technical requirement for log ratio calculation and should not be interpreted as assuming that true biological zeros are impossible. To reduce the influence of sparse taxa and unstable log ratios, OTUs with two or fewer total reads, representing 15.8% of the initial taxa, and OTUs present in only one sample were removed before imputation; this filtering reduced the data set from 3,088 to 2,542 taxa. The fraction of zero entries in the OTU relative-abundance matrix was 55.68% before filtering and 47.39% after filtering. Zero counts were then imputed using Bayesian-multiplicative replacement (GBM; cmultRepl, zCompositions). Sensitivity to zero replacement was assessed by comparing the main GBM imputation with CZM, using Aitchison distance matrices and clr-based OTU rankings (38, 39).

OTU signatures for binary contrasts were identified using the log ratio relevance and glmnet approaches implemented in coda4microbiome, with ALDEx2-based stochastic replication when sample size was limited. ALDEx2 Monte Carlo instances were generated to account for uncertainty in compositional count data. For ALDEx2-based testing, 1,000 Monte Carlo instances were used to estimate Kruskal-Wallis–adjusted *P*-values. In pairwise comparisons with limited sample size, ALDEx2-generated instances were used only as a stochastic sensitivity strategy for OTU signature detection, not as independent biological replicates. Signature strength was evaluated using a Dunn-like index on compositional hierarchical clustering (40). Separation strength was measured using the Sep.HC index, defined as the ratio between the total height of the hierarchical tree and the within-group dispersion of the two sample types. Higher Sep.HC values indicate stronger compositional differentiation between sample types associated with the selected OTUs.

## RESULTS

### Physicochemical characteristics of the sediments

**Fig 2:**
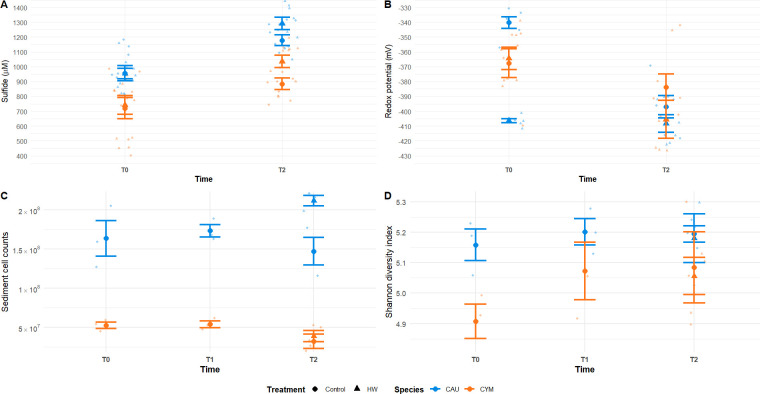
Sediment chemistry, prokaryotic cell counts, and diversity dynamics associated with *Ca. prolifera* and *Cy. nodosa*. (**A**) Sulfide concentrations, (**B**) redox potential (Eh), (**C**) prokaryotic cell concentrations, and (**D**) Shannon diversity index in sediment samples under control and heatwave conditions. Physicochemical variables (**A and B**) were measured only at T0 and T2, whereas microbial abundance and diversity (**C and D**) were assessed at T0, T1, and T2. Symbols represent individual biological replicates; larger points and error bars indicate the mean ± standard error (SE). Colors indicate macrophyte species, whereas symbol type indicates treatment conditions. Statistical comparisons were performed using Wilcoxon rank-sum or Kruskal–Wallis tests with Benjamini–Hochberg correction for multiple comparisons.

Sulfide concentrations ranged from 401 to 1,181 µM in sediment samples upon arrival to the laboratory (T0). Sediments colonized by *Cy. nodosa* exhibited lower sulfide concentrations than those colonized by *Ca. prolifera* (Fig. 2A). At T2, sulfide concentrations were higher than at T0 in both macrophyte-associated sediments, particularly under heatwave conditions. Wilcoxon rank-sum tests comparing independent pots sampled at T0 and T2 showed significant T0–T2 differences in *Ca. prolifera* control and heatwave sediments (BH-adjusted *P* = 0.011 and *P* = 0.002, respectively) and in *Cy. nodosa* heatwave sediments (BH-adjusted *P* = 0.007) but not in *Cy. nodosa* controls.

Redox potential (Eh; Fig. 2B) ranged from −427.7 to −339.1 mV at T0. At T2, Eh values were generally more negative than at T0, with the lowest values observed under heatwave conditions. Wilcoxon rank-sum tests comparing independent pots sampled at T0 and T2 indicated a significant T0–T2 difference in *Ca. prolifera* sediments under control conditions (BH-adjusted *P* = 0.020). In *Cy. nodosa*, the T0–T2 difference under heatwave conditions showed only marginal evidence after correction (BH-adjusted *P* = 0.091), whereas no significant T0–T2 differences were detected in *Ca. prolifera* under heatwave conditions or in *Cy. nodosa* controls. Exploratory difference-in-differences models were used to summarize whether T0–T2 contrasts differed between control and heatwave conditions. In *Cy. nodosa*, the treatment × time interaction was negative, but not statistically significant (β = −24.70, *P* = 0.225). In *Ca. prolifera*, the interaction was positive and significant (β = 54.83, *P* < 0.001), indicating that the T0–T2 decrease observed under control conditions was attenuated under heatwave conditions.

### Changes in microbial counts during the experiment

Microbial loads were overall higher in sediments colonized by *Ca. prolifera* (12 × 10^7^ to 22 × 10^7^ cells/g) compared to those colonized by *Cy. nodosa* (1.9 × 10^7^ to 6.9 × 10^7^ cells/g) (Wilcoxon rank-sum test, *P* = 3.66 × 10^−^⁵; Fig. 2C). When evaluated separately by sampling time, between-species differences were significant at T2 after Benjamini–Hochberg correction (Wilcoxon rank-sum test, raw *P* = 0.005; BH-adjusted *P* = 0.015). Across sampling times, microbial abundance tended to decrease in control sediments associated with both species, although these differences among independently sampled pots were not statistically significant (Kruskal–Wallis test: *Ca. prolifera*, *P* = 0.491; *Cy. nodosa*, *P* = 0.193). At T2, microbial loads were higher under heatwave conditions in sediments colonized by *Ca. prolifera*, whereas sediments associated with *Cy. nodosa* maintained lower values (Fig. 2C).

### Beta-diversity reveals macrophyte-dependent restructuring of microbial communities

A total of 4,243,805 V3–V4 16S rRNA gene sequences were obtained for the 24 samples analyzed, and a total of 3,088 OTUs at 98.7% identity were identified (Table 1). Shannon indices were generally higher in sediments colonized by *Ca. prolifera* than in those colonized by *Cy. nodosa* (Fig. 2D and Table 1). Microbial diversity showed a slight increase during the acclimation period in both macrophyte-associated sediments, followed by relatively stable values during the control and heatwave treatments. This increase appeared more pronounced in *Cy. nodosa*, although the magnitude of the change was modest (Fig. 2D).

**TABLE 1:** Sequencing and diversity metrics of samples

Species	Time	Sample ID	Raw	Trimmed sequences	Number of OTUs	Shannon
*Ca. prolifera*	T0	CAU1	121,821	32,247	1,119	5.22
		CAU3	159,243	44,942	1,005	5.05
		CAU4	104,320	26,153	1,087	5.18
	T1	CAU5	105,634	31,184	1,147	5.27
		CAU7	110,415	30,443	999	5.12
		CAU8	114,635	29,145	1,095	5.19
	T2C	CAU10	122,405	30,386	1,098	5.14
		CAU11	115,278	32,914	1,053	5.19
		CAU12	105,919	26,377	1,121	5.24
	T2HW	CAU14	191,440	62,269	1,115	5.29
		CAU15	159,008	43,539	991	5.02
		CAU16	162,682	50,446	1,083	5.21
*Cy. nodosa*	T0	CYM1	144,994	56,500	1,027	4.80
		CYM2	106,303	38,238	1,097	4.99
		CYM3	140,061	55,898	1,028	4.92
	T1	CYM5	365,352	143,829	1,079	4.91
		CYM7	302,221	121,569	1,076	5.05
		CYM8	219,040	81,297	1,181	5.24
	T2C	CYM10	224,605	96,059	1,125	5.30
		CYM11	253,049	96,159	1,117	5.05
		CYM9	243,451	90,090	1,233	4.89
	T2HW	CYM14	209,425	87,118	1,025	4.93
		CYM15	240,342	93,214	1,150	5.10
		CYM16	222,162	88,533	1,174	5.12

Shannon diversity showed no significant temporal variation under either control conditions (*Ca. prolifera*: Kruskal–Wallis *P* = 0.733; *Cy. nodosa*: *P* = 0.432) or heatwave conditions (*Ca. prolifera*: *P* = 0.875; *Cy. nodosa*: *P* = 0.288), indicating that diversity remained broadly stable throughout the experiment. However, analyses based on Bray-Curtis dissimilarities revealed marked shifts in microbial community structure over time and between macrophyte-associated sediments (File S1). At the initial sampling (T0), microbial communities associated with *Ca. prolifera* and *Cy. nodosa* sediments were highly dissimilar (median Bray-Curtis distance = 0.412). This dissimilarity slightly decreased after the acclimation period (T1; 0.410), increased again under control conditions at T2 (T2C; 0.449), and markedly decreased during the heatwave treatment (T2HW; 0.377). To identify the OTUs driving the observed changes in beta-diversity, we focused on taxa not belonging to the rare biosphere. To define the threshold for the rare biosphere, we evaluated changes in Shannon diversity in relation to OTU relative abundances (41). In sediments colonized by *Ca. prolifera*, OTUs driving changes in Shannon diversity exhibited relative abundances of 0.15%–0.2%, whereas in *Cy. nodosa,* this range was slightly lower (0.1%–0.15%) (File S1). Based on these patterns, OTUs with relative abundances below 0.1% were classified as part of the rare biosphere in this study, analogous to the threshold proposed by Pedrós-Alió (42).

### Initial differences in microbial communities depend on macrophyte identity

**Fig 3:**
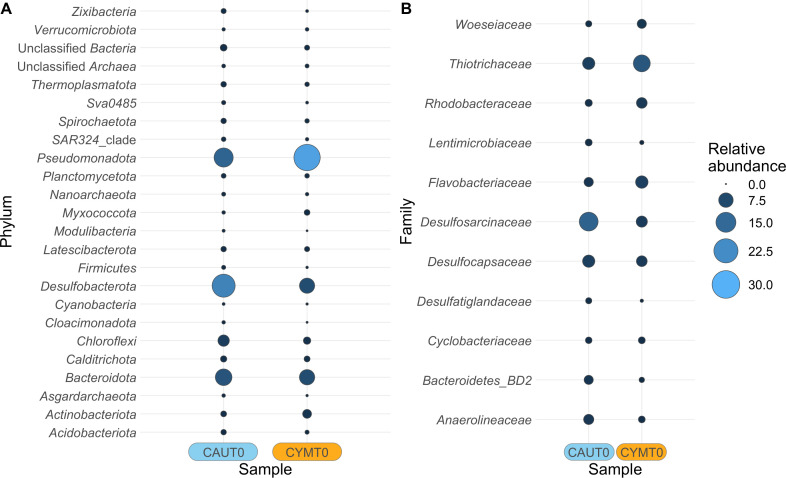
Taxonomic classification of OTUs associated with *Caulerpa* and *Cymodocea* at T0. (**A**) Classification at the phylum level and (**B**) at the family level.

Consistent with the Bray-Curtis–based beta-diversity patterns, hierarchical clustering of microbial community composition clearly differentiated sediments colonized by *Ca. prolifera* and *Cy. nodosa*, with a separation index (Sep.HC) of 2.43 when 1,472 OTUs were used. Feature selection using the log ratio (LR) approach improved sample separation, while the glmnet approach slightly increased discrimination between sample types (Sep.HC = 2.78) using a reduced set of 350 OTUs (Table 2). When restricting the analysis to the 312 OTUs selected by both methods, the separation index remained high (Sep.HC = 3.12). Among the 312 OTUs identified using both approaches, 91 exhibited relative abundances above 0.1% in at least one sample type and were therefore classified as part of the abundant biosphere. These 91 OTUs belonged to 25 different phyla and accounted for more than 50% of all sequences. Notably, sediments colonized by *Ca. prolifera* harbored all these abundant OTUs, whereas sediments associated with *Cy. nodosa* contained 65. In both sediment types, these OTUs primarily belonged to the phyla *Desulfobacterota*, *Pseudomonadota*, *Bacteroidota*, and *Chloroflexota*. However, *Desulfobacterota* was dominant in *Ca. prolifera* colonized sediments, while *Pseudomonadota* was predominant in *Cy. nodosa* sediments (Fig. 3A).

**TABLE 2:** OTU signatures associated with macrophyte identity, acclimation, and heatwave treatment, showing the number of selected OTUs and the corresponding hierarchical clustering separation index (Sep.HC)^*a*^

Comparison		Approach	Number of OTUs	Sep.HC
*Ca. Prolifera* vs. *Cy. nodosa*		Original	1,472	2.43
		LR	549	2.83
		Glmnet	350	2.78
		**Intersection LR and glmnet**	**312**	**3.12**
Effect of acclimation (T0 vs. T1)	*Ca. prolifera*	Original	1,194	no
		**LR**	**24**	**2.3**
		Glmnet	45	0.95
		Intersection LR and glmnet	1	no
	*Cy. nodosa*	Original	1,743	no
		LR	169	0.94
		**Glmnet**	**62**	**1.1**
		Intersection LR and glmnet	10	0.96
Effect of heatwave (T2C vs. T2HW)	*Ca. prolifera*	Original	1,682	no
		**LR**	**95**	**1.98**
		Glmnet	42	1.49
		Intersection LR and glmnet	10	1.96
	*Cy. nodosa*	Original	2,247	1.21
		LR	563	2.15
		Glmnet	81	3.67
		**Intersection LR and glmnet**	**60**	**4.31**

^*a*^ Bold values indicate the approach that achieved the highest hierarchical clustering separation index (Sep.HC) for each comparison.

In sediments colonized by *Ca. prolifera*, predominant OTUs primarily belonged to the phylum *Desulfobacterota*, which accounted for 20.34% ± 2.23% of the total sequences. A significant portion of these OTUs (13.17% ± 0.44%) was attributed to the family *Desulfosarcinaceae*, which was less prevalent in sediments associated with *Cy. nodosa* (4.18% ± 0.53%). In contrast, sediments colonized by *Cy. nodosa* showed a higher relative abundance of *Pseudomonadota*, which comprised 27.01% ± 0.91% of the sequences. Among these, the family *Triotrichaceae*, corresponding to colorless sulfur-oxidizing bacteria, had a particularly high relative abundance (10.46% ± 0.38%). This family was also detected in sediments associated with *Ca. prolifera*, but at a lower and more variable proportion among samples (4.21% ± 1.62%) (Fig. 3B).

### Heatwaves restructure sediment microbial communities associated with *Cy. nodosa* but not *Ca. prolifera*

Hierarchical clustering using all available OTUs did not reveal a clear separation between samples after the acclimation period (i.e., sediments from T1 compared to T0). OTU selection using LR or glmnet yielded only moderate differentiation, driven exclusively by taxa with relative abundances below 0.1%, indicating that microbial communities associated with both *Ca. prolifera* and *Cy. nodosa* sediments remained stable after acclimation. This stability suggests a limited effect of confinement conditions, supporting that differences observed under control and heatwave treatments reflect temperature-driven responses.

**Fig 4:**
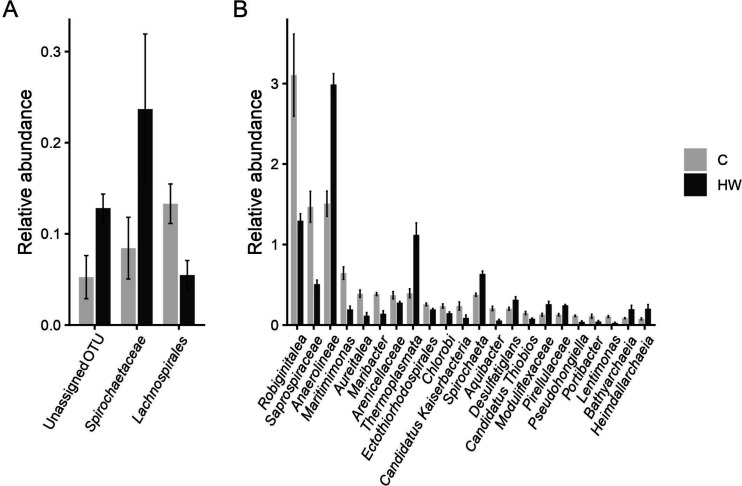
Taxonomic classification of abundant OTUs (>0.1% relative abundance) in sediment microbial communities associated with the seagrasses *Ca. prolifera* (**A**) and *Cy. nodosa* (**B**) under control (C) and heatwave (HW) conditions. Bars represent mean relative abundance values ± standard deviation (SD) of three biological replicates. Different *y*-axis scales were used in panels **A and B** to improve data visualization.

Under heatwave conditions, microbial communities associated with *Ca. prolifera* sediments remained largely unchanged, as hierarchical clustering did not separate control (CAU T2C) and heatwave (CAU T2HW) samples (Table 2). Feature selection approaches identified only 10 OTUs that marked differences, of which just three showed relative abundances above 0.1% (Fig. 4A). In contrast, microbial communities associated with *Cy. nodosa* sediments showed clear separation between control and heatwave conditions, with markedly higher separation indices, particularly when combining LR and glmnet selections (Sep.HC = 4.31; Table 2). This differentiation was primarily driven by changes in the relative abundance of 60 OTUs, of which 29 showed abundances above 0.1% (Fig. 4B). Thus, it appears that the impact of a heatwave event on *Ca. prolifera* colonized sediments primarily affected the rare biosphere, while in *Cy. nodosa,* both the rare and abundant biosphere were influenced by the heatwave.

During the heatwave, *Cy. nodosa* colonized sediments exhibited an increase in potential sulfate-reducing taxa, including *Desulfatiglans* and OTUs affiliated with *Bathyarchaeia*, accompanied by a decrease in putative sulfur oxidizers such as *Candidatus Thiobios* and members of the *Chlorobiota* phylum. Concurrently, taxa associated with aerobic metabolisms declined, while anaerobic microorganisms including *Anaerolineaceae*, *Spirochaeta*, and Marine Benthic Group D archaea increased in relative abundance. This microbial shift toward anaerobic taxa is consistent with the marked decrease in redox potential observed in *Cy. nodosa* colonized sediments during the heatwave, suggesting the development of more reduced and anoxic conditions.

## DISCUSSION

The main objective of this study was to assess microbial community changes in sediments colonized by the seagrass *Cy. nodosa* compared to those colonized by the seaweed *Ca. prolifera* in the Mar Menor lagoon during a heatwave. The prokaryotic community was analyzed using 16S rRNA gene metabarcoding, a widely applied and cost-effective approach for investigating community structure and dynamics. However, as this method is based on total DNA, it captures not only active microorganisms but also extracellular DNA and DNA from inactive or dead cells, which can persist in marine sediments (43). To minimize the risk of over-interpretation associated with these limitations, our analyses focused primarily on changes within the abundant biosphere, which is more likely to reflect active and ecologically relevant microbial responses.

In addition, sequencing-based abundance estimates are influenced by bioinformatic processing, library size variation, and the compositional nature of amplicon data. We therefore used a compositional data analysis framework to analyze OTU relative abundances and identify taxa associated with differences among sample groups. Because different feature-selection procedures may emphasize different subsets of taxa, we combined a log-ratio-based approach (44) with glmnet (39) and focused on OTUs consistently identified by both methods. This strategy was used to obtain a conservative set of OTUs for ecological interpretation, rather than to compare analytical pipelines or advocate for one methodological framework over another. A limitation of this approach is that log ratio analyses require zero replacement. This preprocessing step does not imply that true biological zeros are impossible, but rather provides the strictly positive values required for log ratio calculation. Because high log ratios may arise from very sparse taxa, we filtered rare OTUs before imputation and verified that the overall compositional structure was robust to the zero-replacement procedure. The resulting microbial signatures were therefore interpreted as taxa associated with community shifts rather than as definitive causal drivers.

Since the 2016 dystrophic crisis, *Ca. prolifera* has expanded throughout the Mar Menor, whereas *Cy. nodosa* has shown limited recolonization capacity. In this context, differences between the microbial communities associated with sediments colonized by each macrophyte may help us to better understand potential lagoon responses to climate change. Our data confirm previous observations reporting higher abundances of *Desulfobacterota* in sediments colonized by *Ca. prolifera* compared to those associated with *Cy. nodosa* (23), suggesting that this association is temporally stable. While both sediment types harbor co-abundant sulfate-oxidizing and sulfate-reducing bacteria, sediments associated with *Ca. prolifera* are dominated by sulfate-reducing bacteria, whereas sediments colonized by *Cy. nodosa* exhibit a higher abundance of sulfur-oxidizing bacteria.

Given these microbial differences, it is likely that their physiological responses to heat stress during a heatwave also vary, potentially influencing biogeochemical processes in the sediment and further shaping the ecosystem’s resilience to environmental disturbances. Indeed, in sediments colonized by *Ca. prolifera*, the microbial loads were higher under heatwave conditions; however, no significant changes in diversity were detected, and only three OTUs showed differences in abundance compared to control conditions. One of these OTUs, which was more abundant under heatwave conditions, belongs to the *Spirochaetaceae* family, while the other OTUs, which decreased in abundance, belong to the *Lachnospirales* order. Both groups are typically anaerobic or microaerophilic chemoorganotrophs (45, 46). Thus, microbial communities associated with *Ca. prolifera* colonized sediments appeared to be stable under heatwave conditions, and the higher sulfide concentration observed under heatwave conditions compared to the control is likely due to the higher cell density rather than community shifts.

In contrast, in sediments colonized by *Cy. nodosa*, cell density remained stable, but diversity decreased slightly. Additionally, a total of 29 OTUs exhibited differences in relative abundance when comparing heatwave conditions with the control. Among these OTUs, an increase of sulfate-reducing bacteria such as *Desulfatiglans* was detected. Seagrasses have been shown to provide abundant organic matter to the sediments, and sulfur reducers can utilize this organic matter as electron donors to produce reduced sulfide (13). This interpretation is consistent with the higher sulfide concentrations observed and with the tendency toward more negative Eh values under heatwave conditions, although the redox difference in *Cy. nodosa* was only marginal after multiple-comparison correction. These patterns suggest more reducing conditions that could favor sulfate reduction and the subsequent formation of reduced sulfide. Similar temperature-driven shifts toward sulfate-reducing communities have been previously reported in the rhizosphere of other marine plants, such as *Zostera marina*, where warming has been associated with increased sulfate reduction processes and promotes more reduced sediment conditions (47). Reduced sulfur compounds, especially hydrogen sulfide, are known to be phytotoxic, and their increase has been linked to seagrass die events (48). This potential risk is further exacerbated by the concomitant decrease in sulfur-oxidizing bacteria, such as members of the *Chlorobiota* (49), which could normally contribute to mitigating sulfide toxicity in the Mar Menor lagoon. Consequently, the observed microbial shifts may potentially compromise sediment biogeochemical balance and reduce the resilience of *Cy. nodosa* meadows under marine heatwave conditions.

The Mar Menor lagoon is subjected to intense anthropogenic pressures and is particularly vulnerable to eutrophication, which arises primarily due to the growing population densities along its coastline and the widespread use of fertilizers in agriculture within its surrounding watershed (50, 51). One of the main direct effects of eutrophication is the increase in phytoplankton biomass, which often leads to a reduction in water transparency and an accumulation of organic matter in the sediment. In advanced stages of eutrophication, the growth of the microbial aerobic community in the sediments exacerbates oxygen depletion in the bottom waters, promoting the proliferation of sulfate-reducing bacteria and the production of hydrogen sulfide (52). This process has been linked to unprecedented hypoxia events that lead to dystrophic crises and massive fish kills in the Mar Menor lagoon (53). In this context, the role of sulfur-oxidizing bacteria, particularly those in *Cy. nodosa* colonized sediments, becomes crucial in mitigating these effects and maintaining the proper functioning of the ecosystem. The ongoing substitution of marine seagrasses by seaweeds could further exacerbate these disruptions, influencing the buffering capacity of the ecosystem and threatening its resilience. Furthermore, marine heatwaves, which have become more frequent in the past decade, are likely to reduce the abundance of sulfur-oxidizing bacteria while stimulating sulfate-reducing bacteria, further favoring anoxic conditions that, in combination with eutrophication, could lead to massive mortality events. Therefore, based on our results, marine heatwaves may further weaken the lagoon’s ability to mitigate eutrophication and hypoxia.

### Conclusions

In this study, we show that the response of sediment microbial communities to marine heatwaves in the Mar Menor lagoon depends on the macrophyte species colonizing the sediment. Both sediments showed increased sulfide concentrations over time, particularly under heatwave conditions, suggesting conditions compatible with enhanced sulfate reduction. However, while sediments associated with *Ca. prolifera* displayed higher cell densities but relatively stable microbial structure under the increase in temperature, those colonized by *Cy. nodosa* showed similar cell concentrations but exhibited shifts in community composition, including a slight loss of diversity, an increase in sulfur-reducing bacteria, and a reduction in sulfur-oxidizing bacteria. These changes are particularly relevant because sulfur-oxidizing microorganisms play a key role in limiting sulfide accumulation in seagrass sediments. Their decline under heatwave conditions suggests a reduced capacity of *Cy. nodosa* sediments to buffer sulfide toxicity, potentially increasing the risk of hypoxic conditions in an ecosystem already affected by eutrophication. This process may, in turn, accelerate seagrass loss and favor the expansion of *Ca. prolifera*, whose associated sediments tend to promote sulfate-reducing bacterial communities, reinforcing reduced conditions in the lagoon.

Therefore, our results suggest that marine heatwaves have the potential to affect the resilience of coastal lagoons by altering sediment microbial communities that are essential for maintaining biogeochemical balance. As marine heatwaves become more frequent, incorporating microbial indicators into monitoring and management strategies may be critical to better anticipate dystrophic crises and conserve vulnerable lagoon ecosystems.

## Data Availability

The sequence data from this study have been deposited at the NCBI GenBank database under accession number PRJNA1313870. All data required to reproduce the statistical analyses are available at https://github.com/biocom-uib/CAUvsCYM.

## SUPPLEMENTAL MATERIAL

File S1 msystems.00291-26-s0001.html All statistical analyses conducted for the study.

ASM does not own the copyrights to Supplemental Material that may be linked to, or accessed through, an article. The authors have granted ASM a non-exclusive, world-wide license to publish the Supplemental Material files. Please contact the corresponding author directly for reuse.
