# Supplementary material for: Shifts in microbial communities driven by the replacement of seagrasses by benthic macroalgae may exacerbate heatwave effects in eutrophicated coastal lagoons
Source: mSystems. 2026 Jul 20;11(8):e00291-26. doi: 10.1128/msystems.00291-26 (PMC13492063; doi:10.1128/msystems.00291-26)
Supplement: Supplementary file 1 [file msystems.00291-26-s0001.html]

Shifts in microbial communities driven by the succession of seagrasses to benthic macroalgae may exacerbate heatwave effects in eutrophicated-coastal lagoons: Statistical analysis


# Shifts in microbial communities driven by the succession of seagrasses to benthic macroalgae may exacerbate heatwave effects in eutrophicated-coastal lagoons: Statistical analysis

#### E. Rubio-Portillo, F. Rosselló, B. Aldeguer-Riquelme, *et al*

#### 2026-05-21

This document contains all statistical analyses conducted for the manuscript. All data required to reproduce the analyses are available at: https://github.com/biocom-uib/CAUvsCYM. This document uses precomputed data for computationally intensive steps; see the README for details.

## 1 Setup

```
rm(list = ls())
knitr::opts_chunk$set(
   fig.width=10, 
  out.width="50%",
  fig.asp = 1,
  fig.align="center",
  echo = TRUE,
  message = FALSE, 
  warning = FALSE,
  hiline = TRUE,
  dpi = 72,
  fig.retina = 1,
  cache=TRUE
)
options(knitr.kable.NA = '')
knitr::opts_knit$set(global.par=TRUE)
par(cex.main=0.9,cex.axis=0.8,cex.lab=0.8)
options(scipen = 999)
```

We load the necessary packages:

```
library(readxl)
library(readr)
library(dplyr)
library(tidyr)
library(knitr)
library(ggplot2)
library(grid)
library(tidyverse)
library(splines)
library(zCompositions)
library(compositions)
library(robCompositions)
library(easyCODA)
library(ALDEx2)
library(viridis)
library(plotly)
library(ComplexHeatmap)
library(stats)
library(dendextend)
library(RColorBrewer)
library(kableExtra)
library(vegan)
library(ecolTest)
library(factoextra)
library(coda4microbiome)
library(broom)
library(flextable)
source("funcionsCODAMETACIRCLE.R")

opar=par()

set_flextable_defaults(
  font.family = "Arial", font.size = 10, 
  border.color = "gray", big.mark = "")
```

## 2 Statistical analysis of biochemical and cellular parameters

Because samples collected at different time points came from different pots rather than repeated measurements of the same experimental units, temporal comparisons are reported as contrasts among independent pots sampled at each time point. Non-parametric pairwise comparisons were performed using Wilcoxon rank-sum tests. Whenever multiple pairwise comparisons were conducted across species, treatments or sampling times, p-values were adjusted using the Benjamini–Hochberg false discovery rate procedure, and significance was interpreted using adjusted p-values.

### 2.1 Sulfide context

```
# Data and factors
datos <- read_table("Sulfur_data_28ago25.txt")
datos$Species <- as.factor(datos$Species)
datos$Treatment <- as.factor(datos$Treatment)
datos$Time <- factor(datos$Time, levels = c("T0", "T2"))
```

```
# Group summary for plotting
df_summary <- datos %>%
  dplyr::group_by(Time, Species, Treatment) %>%
  dplyr::summarise(
    mean_sulfide = mean(Sulfur, na.rm = TRUE),
    sd_sulfide   = sd(Sulfur, na.rm = TRUE),
    n            = n(),
    se_sulfide   = sd_sulfide / sqrt(n),
    .groups      = "drop"
  )

p_a <- ggplot() +
  geom_jitter(
    data = datos,
    aes(x = Time, y = Sulfur, color = Species, shape = Treatment),
    width = 0.1,
    alpha = 0.4,
    size = 1.2
  ) +
  geom_point(
    data = df_summary,
    aes(x = Time, y = mean_sulfide, color = Species, shape = Treatment),
    size = 3
  ) +
  geom_errorbar(
    data = df_summary,
    aes(x = Time,
        ymin = mean_sulfide - se_sulfide,
        ymax = mean_sulfide + se_sulfide,
        color = Species),
    width = 0.1,
    size = 1.2
  ) +
  scale_color_manual(values = c("CAU" = "#008ae6", "CYM" = "#ff751a")) +
  labs(
    x = "Time",
    y = expression(paste("Sulfide (", mu, "M)")),
    color = "Species",
    shape = "Treatment",
    linetype = "Treatment"
  ) +
  theme_minimal() +
  theme(
    axis.title.x = element_text(face = "bold"),
    axis.title.y = element_text(face = "bold"),
    axis.text.x  = element_text(face = "bold"),
    legend.title = element_text(face = "bold")
  ) +
  coord_cartesian(ylim = c(400, 1400)) +
  scale_y_continuous(breaks = seq(400, 1400, by = 100))

p_a
```

The figure provides a descriptive overview of sulfide concentrations (mean ± SE) at baseline (T0) and after exposure (T2) for sediments colonized by *Caulerpa* (blue) and *Cymodocea* (orange) under control and heatwave conditions. Individual data points illustrate within-group variability. Because different pots were sampled at T0 and T2, comparisons between sampling times represent contrasts among independent pots rather than repeated measurements of the same pots.

We first report group medians at T0 and T2 and their difference (\(\Delta = T2 - T0\)) for each Species \(\times\) Treatment as descriptive summaries.

```
medians <- datos %>%
  dplyr::group_by(Species, Treatment, Time) %>%
  dplyr::summarise(
    median_sulfide = median(Sulfur, na.rm = TRUE),
    n = dplyr::n(),
    .groups = "drop"
  ) %>%
  tidyr::pivot_wider(names_from = Time, values_from = median_sulfide) %>%
  dplyr::mutate(delta_median = `T2` - `T0`)

knitr::kable(medians, digits = 2, caption = "Medians by group and change (T2 - T0)")
```

Table 1: Medians by group and change (T2 - T0)

| Species | Treatment | n | T0 | T2 | delta\_median |
| --- | --- | --- | --- | --- | --- |
| CAU | Control | 9 | 945.91 | 1151.37 | 205.46 |
| CAU | HW | 9 | 939.62 | 1284.93 | 345.31 |
| CYM | Control | 9 | 785.53 | 894.56 | 109.03 |
| CYM | HW | 9 | 835.85 | 1109.06 | 273.21 |

We then formally tested T0 vs T2 within each Species \(\times\) Treatment using Wilcoxon rank-sum tests, controlling the false discovery rate with Benjamini–Hochberg adjustment.

```
time_tests <- datos %>%
  dplyr::group_by(Species, Treatment) %>%
  dplyr::summarise(
    p = {
      d <- dplyr::cur_data()
      x <- d$Sulfur[d$Time == "T0"]
      y <- d$Sulfur[d$Time == "T2"]
      if (length(x) > 0 && length(y) > 0) {
        suppressWarnings(stats::wilcox.test(x, y, exact = FALSE)$p.value)
      } else {
        NA_real_
      }
    },
    .groups = "drop"
  ) %>%
  dplyr::mutate(p_adj = p.adjust(p, method = "BH"))

knitr::kable(
  time_tests,
  digits = 3,
  caption = "Wilcoxon rank-sum tests comparing T0 vs T2 per Species × Treatment (BH-adjusted p)"
)
```

Table 2: Wilcoxon rank-sum tests comparing T0 vs T2 per Species × Treatment (BH-adjusted p)

| Species | Treatment | p | p\_adj |
| --- | --- | --- | --- |
| CAU | Control | 0.008 | 0.011 |
| CAU | HW | 0.001 | 0.002 |
| CYM | Control | 0.158 | 0.158 |
| CYM | HW | 0.004 | 0.007 |

Treatment differences at T2 were also compared within each species, with Benjamini–Hochberg adjustment across species-specific comparisons.

```
sulfide_t2_bt <- datos %>%
  dplyr::filter(Time == "T2") %>%
  dplyr::group_by(Species) %>%
  dplyr::summarise(
    p = {
      d <- dplyr::cur_data()
      if (dplyr::n_distinct(d$Treatment) >= 2) {
        suppressWarnings(stats::wilcox.test(Sulfur ~ Treatment, data = d, exact = FALSE)$p.value)
      } else {
        NA_real_
      }
    },
    .groups = "drop"
  ) %>%
  dplyr::mutate(p_adj = p.adjust(p, method = "BH"))

knitr::kable(
  sulfide_t2_bt,
  digits = 3,
  caption = "Wilcoxon rank-sum tests comparing treatments at T2 by Species (BH-adjusted p)"
)
```

Table 3: Wilcoxon rank-sum tests comparing treatments at T2 by Species (BH-adjusted p)

| Species | p | p\_adj |
| --- | --- | --- |
| CAU | 0.064 | 0.064 |
| CYM | 0.038 | 0.064 |

Overall, sulfide concentrations were higher in T2 samples than in T0 samples for several Species \(\times\) Treatment groups, but statistical significance was interpreted using BH-adjusted p-values. The models below provide an exploratory summary of whether T0–T2 contrasts differed between control and heatwave conditions.

#### 2.1.1 Difference-in-differences-type models

Heatwave effects were further explored using difference-in-differences-type models to assess whether T0–T2 contrasts differed between control and heatwave conditions. Because different pots were sampled at each time point, these models were interpreted as exploratory treatment \(\times\) time interaction models rather than as formal longitudinal causal estimates. For each species, the response was modeled as a function of treatment, time, and their interaction.

*Cymodocea — sulfide analysis*

```
cy <- datos %>% dplyr::filter(Species == "CYM")
cy$Treatment <- factor(cy$Treatment, levels = c("Control", "HW"))
cy$Time <- factor(cy$Time, levels = c("T0", "T2"))

fit_cy <- lm(Sulfur ~ Treatment + Time + Treatment:Time, data = cy)
summary(fit_cy)
```

```
## 
## Call:
## lm(formula = Sulfur ~ Treatment + Time + Treatment:Time, data = cy)
## 
## Residuals:
##     Min      1Q  Median      3Q     Max 
## -318.31 -118.50   32.97   95.49  266.60 
## 
## Coefficients:
##                    Estimate Std. Error t value           Pr(>|t|)    
## (Intercept)          720.20      56.15  12.826 0.0000000000000372 ***
## TreatmentHW           21.73      79.41   0.274             0.7861    
## TimeT2               163.62      79.41   2.061             0.0476 *  
## TreatmentHW:TimeT2   129.66     112.30   1.155             0.2568    
## ---
## Signif. codes:  0 '***' 0.001 '**' 0.01 '*' 0.05 '.' 0.1 ' ' 1
## 
## Residual standard error: 168.5 on 32 degrees of freedom
## Multiple R-squared:  0.3877, Adjusted R-squared:  0.3303 
## F-statistic: 6.754 on 3 and 32 DF,  p-value: 0.001172
```

```
knitr::kable(broom::tidy(fit_cy), digits = 3, caption = "Exploratory treatment × time model for sulfide in Cymodocea")
```

Table 4: Exploratory treatment × time model for sulfide in Cymodocea

| term | estimate | std.error | statistic | p.value |
| --- | --- | --- | --- | --- |
| (Intercept) | 720.196 | 56.151 | 12.826 | 0.000 |
| TreatmentHW | 21.733 | 79.410 | 0.274 | 0.786 |
| TimeT2 | 163.624 | 79.410 | 2.061 | 0.048 |
| TreatmentHW:TimeT2 | 129.660 | 112.303 | 1.155 | 0.257 |

*Caulerpa — sulfide analysis*

```
ca <- datos %>% dplyr::filter(Species == "CAU")
ca$Treatment <- factor(ca$Treatment, levels = c("Control", "HW"))
ca$Time <- factor(ca$Time, levels = c("T0", "T2"))

fit_ca <- lm(Sulfur ~ Treatment + Time + Treatment:Time, data = ca)
summary(fit_ca)
```

```
## 
## Call:
## lm(formula = Sulfur ~ Treatment + Time + Treatment:Time, data = ca)
## 
## Residuals:
##      Min       1Q   Median       3Q      Max 
## -242.770  -71.945   -7.951   83.563  226.415 
## 
## Coefficients:
##                    Estimate Std. Error t value             Pr(>|t|)    
## (Intercept)         955.346     40.921  23.346 < 0.0000000000000002 ***
## TreatmentHW          -1.572     57.872  -0.027             0.978493    
## TimeT2              222.755     57.872   3.849             0.000534 ***
## TreatmentHW:TimeT2  114.871     81.843   1.404             0.170080    
## ---
## Signif. codes:  0 '***' 0.001 '**' 0.01 '*' 0.05 '.' 0.1 ' ' 1
## 
## Residual standard error: 122.8 on 32 degrees of freedom
## Multiple R-squared:  0.6131, Adjusted R-squared:  0.5769 
## F-statistic: 16.91 on 3 and 32 DF,  p-value: 0.0000009271
```

```
knitr::kable(broom::tidy(fit_ca), digits = 3, caption = "Exploratory treatment × time model for sulfide in Caulerpa")
```

Table 5: Exploratory treatment × time model for sulfide in Caulerpa

| term | estimate | std.error | statistic | p.value |
| --- | --- | --- | --- | --- |
| (Intercept) | 955.346 | 40.921 | 23.346 | 0.000 |
| TreatmentHW | -1.572 | 57.872 | -0.027 | 0.978 |
| TimeT2 | 222.755 | 57.872 | 3.849 | 0.001 |
| TreatmentHW:TimeT2 | 114.871 | 81.843 | 1.404 | 0.170 |

The treatment \(\times\) time interaction term was used to summarize whether the T0–T2 contrast differed between control and heatwave conditions. These results were interpreted cautiously because the model summarizes differences among independent pots sampled at each time point.

### 2.2 Redox potential

```
# Data used only for the redox analysis block
datos <- read_table("redox_data_12feb25.txt")
datos$Species <- as.factor(datos$Species)
datos$Treatment <- as.factor(datos$Treatment)
datos$Time <- factor(datos$Time, levels = c("T0", "T2"))
```

```
# Group summary for plotting
df_summary <- datos %>%
  dplyr::group_by(Time, Species, Treatment) %>%
  dplyr::summarise(
    mean_redox = mean(redox, na.rm = TRUE),
    sd_redox   = sd(redox, na.rm = TRUE),
    n          = n(),
    se_redox   = sd_redox / sqrt(n),
    .groups    = "drop"
  )

p_b <- ggplot() +
  geom_jitter(
    data = datos,
    aes(x = Time, y = redox, color = Species, shape = Treatment),
    width = 0.1,
    alpha = 0.4,
    size = 1.2
  ) +
  geom_point(
    data = df_summary,
    aes(x = Time, y = mean_redox, color = Species, shape = Treatment),
    size = 3
  ) +
  geom_errorbar(
    data = df_summary,
    aes(x = Time,
        ymin = mean_redox - se_redox,
        ymax = mean_redox + se_redox,
        color = Species),
    width = 0.1,
    size = 1.2
  ) +
  scale_color_manual(values = c("CAU" = "#008ae6", "CYM" = "#ff751a")) +
  labs(
    x = "Time",
    y = expression(paste("redox (", m, "V)")),
    color = "Species",
    shape = "Treatment",
    linetype = "Treatment"
  ) +
  theme_minimal() +
  theme(
    axis.title.x = element_text(face = "bold"),
    axis.title.y = element_text(face = "bold"),
    axis.text.x = element_text(face = "bold"),
    legend.title = element_text(face = "bold")
  ) +
  coord_cartesian(ylim = c(-430, -330)) +
  scale_y_continuous(breaks = seq(-430, -330, by = 10))

p_b
```

The figure provides a descriptive overview of sediment redox potential (mean ± SE) at baseline (T0) and after exposure (T2) for sediments colonized by *Caulerpa* and *Cymodocea* under control and heatwave conditions. Because different pots were sampled at T0 and T2, comparisons between sampling times represent contrasts among independent pots rather than repeated measurements of the same pots.

To formally assess these visual patterns, we evaluated T0–T2 contrasts for each Species \(\times\) Treatment using Wilcoxon rank-sum tests and compared treatments at T2 within each species. P-values were adjusted for multiple testing using the Benjamini–Hochberg procedure.

```
eh_within <- datos %>%
  dplyr::group_by(Species, Treatment) %>%
  dplyr::summarise(
    p = {
      d <- dplyr::cur_data()
      x <- d$redox[d$Time == "T0"]
      y <- d$redox[d$Time == "T2"]
      if (length(x) > 0 && length(y) > 0) {
        suppressWarnings(stats::wilcox.test(x, y, exact = FALSE)$p.value)
      } else {
        NA_real_
      }
    },
    .groups = "drop"
  ) %>%
  dplyr::mutate(p_adj = p.adjust(p, method = "BH"))

knitr::kable(
  eh_within,
  digits = 3,
  caption = "Wilcoxon rank-sum tests comparing T0 vs T2 by Species × Treatment (BH-adjusted p)"
)
```

Table 6: Wilcoxon rank-sum tests comparing T0 vs T2 by Species × Treatment (BH-adjusted p)

| Species | Treatment | p | p\_adj |
| --- | --- | --- | --- |
| CAU | Control | 0.005 | 0.020 |
| CAU | HW | 0.575 | 0.575 |
| CYM | Control | 0.298 | 0.397 |
| CYM | HW | 0.045 | 0.091 |

```
eh_t2_bt <- datos %>%
  dplyr::filter(Time == "T2") %>%
  dplyr::group_by(Species) %>%
  dplyr::summarise(
    p = {
      d <- dplyr::cur_data()
      if (dplyr::n_distinct(d$Treatment) >= 2) {
        suppressWarnings(stats::wilcox.test(redox ~ Treatment, data = d, exact = FALSE)$p.value)
      } else {
        NA_real_
      }
    },
    .groups = "drop"
  ) %>%
  dplyr::mutate(p_adj = p.adjust(p, method = "BH"))

knitr::kable(
  eh_t2_bt,
  digits = 3,
  caption = "Wilcoxon rank-sum tests comparing treatments at T2 by Species (BH-adjusted p)"
)
```

Table 7: Wilcoxon rank-sum tests comparing treatments at T2 by Species (BH-adjusted p)

| Species | p | p\_adj |
| --- | --- | --- |
| CAU | 0.298 | 0.298 |
| CYM | 0.045 | 0.091 |

Overall, redox potential tended to be more negative in T2 samples than in T0 samples, but the magnitude and statistical support of these contrasts varied across species and treatments. Significance was interpreted using BH-adjusted p-values.

#### 2.2.1 Difference-in-differences-type models

We then fitted exploratory difference-in-differences-type models for each species to summarize whether T0–T2 contrasts in redox potential differed between control and heatwave conditions. Because different pots were sampled at each time point, these models were interpreted as treatment \(\times\) time interaction models rather than as formal longitudinal causal estimates.

*Cymodocea — redox analysis*

```
cy <- datos %>% dplyr::filter(Species == "CYM")
cy$Treatment <- factor(cy$Treatment, levels = c("Control", "HW"))
cy$Time <- factor(cy$Time, levels = c("T0", "T2"))

fit_cy <- lm(redox ~ Treatment + Time + Treatment:Time, data = cy)
summary(fit_cy)
```

```
## 
## Call:
## lm(formula = redox ~ Treatment + Time + Treatment:Time, data = cy)
## 
## Residuals:
##     Min      1Q  Median      3Q     Max 
## -42.133 -15.604  -4.217  12.767  59.883 
## 
## Coefficients:
##                    Estimate Std. Error t value            Pr(>|t|)    
## (Intercept)         -367.57       9.87 -37.242 <0.0000000000000002 ***
## TreatmentHW            3.10      13.96   0.222               0.826    
## TimeT2               -16.32      13.96  -1.169               0.256    
## TreatmentHW:TimeT2   -24.70      19.74  -1.251               0.225    
## ---
## Signif. codes:  0 '***' 0.001 '**' 0.01 '*' 0.05 '.' 0.1 ' ' 1
## 
## Residual standard error: 24.18 on 20 degrees of freedom
## Multiple R-squared:  0.3523, Adjusted R-squared:  0.2552 
## F-statistic: 3.627 on 3 and 20 DF,  p-value: 0.03075
```

```
knitr::kable(broom::tidy(fit_cy), digits = 3, caption = "Exploratory treatment × time model for redox potential in Cymodocea")
```

Table 8: Exploratory treatment × time model for redox potential in Cymodocea

| term | estimate | std.error | statistic | p.value |
| --- | --- | --- | --- | --- |
| (Intercept) | -367.567 | 9.870 | -37.242 | 0.000 |
| TreatmentHW | 3.100 | 13.958 | 0.222 | 0.826 |
| TimeT2 | -16.317 | 13.958 | -1.169 | 0.256 |
| TreatmentHW:TimeT2 | -24.700 | 19.739 | -1.251 | 0.225 |

*Caulerpa — redox analysis*

```
ca <- datos %>% dplyr::filter(Species == "CAU")
ca$Treatment <- factor(ca$Treatment, levels = c("Control", "HW"))
ca$Time <- factor(ca$Time, levels = c("T0", "T2"))

fit_ca <- lm(redox ~ Treatment + Time + Treatment:Time, data = ca)
summary(fit_ca)
```

```
## 
## Call:
## lm(formula = redox ~ Treatment + Time + Treatment:Time, data = ca)
## 
## Residuals:
##      Min       1Q   Median       3Q      Max 
## -21.1833  -5.8750   0.7333   5.6438  27.6167 
## 
## Coefficients:
##                    Estimate Std. Error t value             Pr(>|t|)    
## (Intercept)        -340.325      5.159 -65.969 < 0.0000000000000002 ***
## TreatmentHW         -66.158      7.296  -9.068          0.000000016 ***
## TimeT2              -56.692      7.296  -7.770          0.000000182 ***
## TreatmentHW:TimeT2   54.825     10.318   5.314          0.000033565 ***
## ---
## Signif. codes:  0 '***' 0.001 '**' 0.01 '*' 0.05 '.' 0.1 ' ' 1
## 
## Residual standard error: 12.64 on 20 degrees of freedom
## Multiple R-squared:  0.8539, Adjusted R-squared:  0.8319 
## F-statistic: 38.95 on 3 and 20 DF,  p-value: 0.00000001531
```

```
knitr::kable(broom::tidy(fit_ca), digits = 3, caption = "Exploratory treatment × time model for redox potential in Caulerpa")
```

Table 9: Exploratory treatment × time model for redox potential in Caulerpa

| term | estimate | std.error | statistic | p.value |
| --- | --- | --- | --- | --- |
| (Intercept) | -340.325 | 5.159 | -65.969 | 0 |
| TreatmentHW | -66.158 | 7.296 | -9.068 | 0 |
| TimeT2 | -56.692 | 7.296 | -7.770 | 0 |
| TreatmentHW:TimeT2 | 54.825 | 10.318 | 5.314 | 0 |

The treatment \(\times\) time interaction term was used to summarize whether the T0–T2 contrast differed between control and heatwave conditions. These results were interpreted cautiously because the model summarizes differences among independent pots sampled at each time point.

### 2.3 Sediment cell counts

We analyzed cell counts in sediments colonized by either *Caulerpa* or *Cymodocea* under control and heatwave conditions. We first describe the patterns across sampling times (T0–T1–T2) within each Species \(\times\) Treatment group and then report the corresponding inferential comparisons, followed by between-species contrasts. Because different pots were sampled at each time point, temporal comparisons are interpreted as contrasts among independent pots sampled at each time point.

```
# Data
datos <- read_table("cell_shanon_data_12_05_25.txt") %>%
  dplyr::mutate(
    Species = factor(Species),
    Time = factor(Time, levels = c("T0", "T1", "T2")),
    Treatment = factor(Treatment),
    cells = as.numeric(cells)
  )

# Group summary for plotting
summary_data <- datos %>%
  dplyr::group_by(Species, Treatment, Time) %>%
  dplyr::summarise(
    mean_cells = mean(cells, na.rm = TRUE),
    sd = sd(cells, na.rm = TRUE),
    n = n(),
    se = sd / sqrt(n),
    .groups = "drop"
  )

t1_hw <- summary_data %>%
  dplyr::filter(Treatment == "HW", Time == "T1") %>%
  dplyr::select(Species, y = mean_cells)

t2_hw <- summary_data %>%
  dplyr::filter(Treatment == "HW", Time == "T2") %>%
  dplyr::select(Species, yend = mean_cells)

segments_hw <- dplyr::left_join(t1_hw, t2_hw, by = "Species")

t12 <- summary_data %>% dplyr::filter(Time %in% c("T1", "T2"))
rango12 <- range(t12$mean_cells - t12$se, t12$mean_cells + t12$se)
ymin12 <- rango12[1] - 0.025 * diff(rango12)
ymax12 <- rango12[2] + 0.025 * diff(rango12)

p_c <- ggplot() +
  geom_jitter(
    data = datos,
    aes(x = Time, y = cells, color = Species, shape = Treatment),
    width = 0.1,
    alpha = 0.4,
    size = 1.2
  ) +
  geom_point(
    data = summary_data,
    aes(x = Time, y = mean_cells, color = Species, shape = Treatment),
    size = 3
  ) +
  geom_errorbar(
    data = summary_data,
    aes(x = Time,
        ymin = mean_cells - se,
        ymax = mean_cells + se,
        color = Species),
    width = 0.2,
    size = 1.25
  ) +
  scale_color_manual(
    name = "Species",
    values = c("CAU" = "#008ae6", "CYM" = "#ff751a")
  ) +
  scale_shape_manual(
    name = "Treatment",
    values = c("Control" = 16, "HW" = 17)
  ) +
  scale_linetype_manual(
    name = "Treatment",
    values = c("Control" = "solid", "HW" = "dashed")
  ) +
  guides(
    shape = guide_legend(override.aes = list(size = 3)),
    linetype = guide_legend(override.aes = list(size = 1.5))
  ) +
  labs(
    x = "Time",
    y = "Cells",
    color = "Species",
    shape = "Treatment",
    linetype = "Treatment"
  ) +
  theme_minimal(base_size = 12) +
  theme(
    axis.title.x = element_text(face = "bold"),
    axis.title.y = element_text(face = "bold"),
    axis.text.x = element_text(face = "bold"),
    legend.title = element_text(face = "bold")
  ) +
  coord_cartesian(ylim = c(ymin12, ymax12)) +
  scale_y_continuous(
    breaks = pretty(c(ymin12, ymax12), n = 5),
    labels = expression(0, 5 %*% 10^7, 1 %*% 10^8, 1.5 %*% 10^8, 2 %*% 10^8, 2.5 %*% 10^8),
    expand = expansion(mult = c(0, 0))
  )

p_c
```

The figure shows cell counts in sediments colonized by *Caulerpa* and *Cymodocea* under control and heatwave conditions across three sampling times (T0, T1, T2). The figure is interpreted descriptively, and formal tests are reported below.

We first quantified the overall range of cell counts observed in each species to provide context for the magnitude of the differences.

```
ranges <- datos %>%
  dplyr::group_by(Species) %>%
  dplyr::summarise(
    min_cells = min(cells, na.rm = TRUE),
    max_cells = max(cells, na.rm = TRUE),
    .groups = "drop"
  ) %>%
  dplyr::mutate(
    min_x1e9 = round(min_cells / 1e9, 1),
    max_x1e9 = round(max_cells / 1e9, 1)
  )

knitr::kable(ranges, digits = 2, caption = "Species-wise ranges (×10^9 cells/g)")
```

Table 10: Species-wise ranges (×10^9 cells/g)

| Species | min\_cells | max\_cells | min\_x1e9 | max\_x1e9 |
| --- | --- | --- | --- | --- |
| CAU | 116000000 | 221000000 | 0.1 | 0.2 |
| CYM | 19800000 | 61900000 | 0.0 | 0.1 |

To evaluate species-level differences, we first applied a Wilcoxon rank-sum test pooling across all sampling times and treatments. This overall comparison was used as a descriptive global contrast, because it pools observations across experimental conditions. We then compared species separately at each sampling time and adjusted p-values across these time-specific comparisons using the Benjamini–Hochberg procedure.

```
# Overall (pooling all times/treatments)
bt_overall <- suppressWarnings(stats::wilcox.test(cells ~ Species, data = datos, exact = FALSE))

# By time (T0, T1, T2), with BH adjustment across time-specific comparisons
bt_by_time <- datos %>%
  dplyr::group_by(Time) %>%
  dplyr::summarise(
    p = {
      d <- dplyr::cur_data()
      suppressWarnings(stats::wilcox.test(d$cells ~ d$Species, exact = FALSE)$p.value)
    },
    .groups = "drop"
  ) %>%
  dplyr::mutate(p_adj = p.adjust(p, method = "BH"))

knitr::kable(bt_by_time, digits = 3, caption = "Between-species Wilcoxon rank-sum tests by sampling time (BH-adjusted p)")
```

Table 11: Between-species Wilcoxon rank-sum tests by sampling time (BH-adjusted p)

| Time | p | p\_adj |
| --- | --- | --- |
| T0 | 0.081 | 0.081 |
| T1 | 0.081 | 0.081 |
| T2 | 0.005 | 0.015 |

```
bt_overall$p.value
```

```
## [1] 0.00003658455
```

This overall comparison indicated that cell counts differed between species, with *Caulerpa* generally showing higher values than *Cymodocea* across the experiment. Time-specific comparisons were interpreted using BH-adjusted p-values.

To assess whether cell counts differed among sampling times within each Species \(\times\) Treatment combination, we first applied a nonparametric global test across the three sampling points (T0, T1, T2). Pairwise Wilcoxon rank-sum tests were then performed within each Species \(\times\) Treatment group, with Benjamini–Hochberg adjustment across the three pairwise contrasts.

```
# Kruskal-Wallis per group
kw_time <- datos %>%
  dplyr::group_by(Species, Treatment) %>%
  dplyr::summarise(
    kw_p = {
      d <- dplyr::cur_data()
      if (dplyr::n_distinct(d$Time) >= 2) {
        suppressWarnings(kruskal.test(cells ~ Time, data = d)$p.value)
      } else {
        NA_real_
      }
    },
    .groups = "drop"
  )

# Pairwise Wilcoxon per group (BH across the 3 pairwise contrasts within each group)
pw_time <- datos %>%
  dplyr::group_by(Species, Treatment) %>%
  dplyr::group_modify(~{
    d <- .x
    if (dplyr::n_distinct(d$Time) < 2) return(tibble())

    pw <- pairwise.wilcox.test(d$cells, d$Time, p.adjust.method = "BH", exact = FALSE)

    M <- as.data.frame(as.table(pw$p.value))
    names(M) <- c("Time1", "Time2", "p_adj")
    M <- M %>% dplyr::filter(!is.na(p_adj))

    meds <- d %>% dplyr::group_by(Time) %>% dplyr::summarise(med = median(cells, na.rm = TRUE), .groups = "drop")

    M <- M %>%
      dplyr::left_join(meds, by = c("Time1" = "Time")) %>% dplyr::rename(med1 = med) %>%
      dplyr::left_join(meds, by = c("Time2" = "Time")) %>% dplyr::rename(med2 = med) %>%
      dplyr::mutate(direction = dplyr::case_when(
        med2 > med1 ~ "increase",
        med2 < med1 ~ "decrease",
        TRUE ~ "no_change"
      ))
    M
  }) %>%
  dplyr::ungroup()

knitr::kable(kw_time, digits = 3, caption = "Kruskal-Wallis tests across T0-T1-T2 by Species × Treatment")
```

Table 12: Kruskal-Wallis tests across T0-T1-T2 by Species × Treatment

| Species | Treatment | kw\_p |
| --- | --- | --- |
| CAU | Control | 0.491 |
| CAU | HW |  |
| CYM | Control | 0.193 |
| CYM | HW |  |

```
knitr::kable(pw_time, digits = 3, caption = "Pairwise Wilcoxon rank-sum tests across sampling times (BH-adjusted p) and median directions")
```

Table 12: Pairwise Wilcoxon rank-sum tests across sampling times (BH-adjusted p) and median directions

| Species | Treatment | Time1 | Time2 | p\_adj | med1 | med2 | direction |
| --- | --- | --- | --- | --- | --- | --- | --- |
| CAU | Control | T1 | T0 | 0.663 | 168000000 | 159000000 | decrease |
| CAU | Control | T2 | T0 | 0.663 | 148000000 | 159000000 | increase |
| CAU | Control | T2 | T1 | 0.663 | 148000000 | 168000000 | increase |
| CYM | Control | T1 | T0 | 1.000 | 52400000 | 54000000 | increase |
| CYM | Control | T2 | T0 | 0.286 | 26500000 | 54000000 | increase |
| CYM | Control | T2 | T1 | 0.286 | 26500000 | 52400000 | increase |

The nonparametric tests did not detect statistically significant within-group differences among sampling times. The graphical patterns may indicate biologically relevant variation, but these patterns should be interpreted cautiously because of the limited sample size, between-pot variability, and the fact that different pots were sampled at each time point.

## 3 Zero-inflation and zero-replacement sensitivity analysis

This section quantifies the fraction of zero entries in the OTU relative-abundance matrix before and after filtering rare and singleton OTUs. It also assesses the sensitivity of the compositional structure to the zero-replacement procedure by comparing the main GBM imputation with an alternative CZM imputation using Aitchison distances and clr-based OTU rankings.

```
options(scipen = 999)

required_packages <- c("readxl", "zCompositions", "vegan")


library(readxl)
library(zCompositions)
library(vegan)

# -------------------------
# Settings
# -------------------------

data_file <- "TablaOTUs.xlsx"

# Remove OTUs with <= 2 total reads
low_count_threshold <- 2

# Number of top OTUs used for the GBM-vs-CZM sensitivity check
top_k <- 100

# -------------------------
# Functions
# -------------------------

remove_cols_safe <- function(mat, cols_to_remove) {
  if (length(cols_to_remove) == 0) return(mat)
  mat[, -cols_to_remove, drop = FALSE]
}

closure_rows <- function(mat) {
  mat <- as.matrix(mat)
  storage.mode(mat) <- "numeric"
  
  rs <- rowSums(mat)
  
  if (any(rs == 0)) {
    stop("At least one sample has total count equal to zero.")
  }
  
  sweep(mat, 1, rs, FUN = "/")
}

zero_summary_row <- function(mat, step) {
  rel_mat <- closure_rows(mat)
  
  data.frame(
    step = step,
    samples = nrow(rel_mat),
    taxa = ncol(rel_mat),
    cells = length(rel_mat),
    zeros = sum(rel_mat == 0, na.rm = TRUE),
    zero_fraction = mean(rel_mat == 0, na.rm = TRUE),
    zero_percent = 100 * mean(rel_mat == 0, na.rm = TRUE),
    stringsAsFactors = FALSE
  )
}

impute_safe <- function(mat, method) {
  out <- tryCatch(
    zCompositions::cmultRepl(
      mat,
      method = method,
      output = "p-counts",
      suppress.print = TRUE,
      z.warning = 0.99
    ),
    error = function(e) {
      warning(
        "cmultRepl method '", method, "' failed: ", conditionMessage(e),
        ". Falling back to a small pseudocount replacement."
      )
      
      mat2 <- as.matrix(mat)
      storage.mode(mat2) <- "numeric"
      
      min_positive <- min(mat2[mat2 > 0], na.rm = TRUE)
      pseudo <- min_positive / 2
      mat2[mat2 == 0] <- pseudo
      
      sweep(mat2, 1, rowSums(mat2), FUN = "/") * rowSums(mat)
    }
  )
  
  out <- as.matrix(out)
  rownames(out) <- rownames(mat)
  colnames(out) <- colnames(mat)
  out
}

clr_transform <- function(mat) {
  rel_mat <- closure_rows(mat)
  log_mat <- log(rel_mat)
  sweep(log_mat, 1, rowMeans(log_mat), FUN = "-")
}

aitchison_dist <- function(mat) {
  dist(clr_transform(mat), method = "euclidean")
}

simple_clr_signature <- function(imputed_counts, groups, top_k = 100) {
  clr_mat <- clr_transform(imputed_counts)
  
  groups <- factor(groups)
  
  if (nlevels(groups) != 2) {
    stop("groups must have exactly two levels.")
  }
  
  lev <- levels(groups)
  
  effect <- colMeans(clr_mat[groups == lev[1], , drop = FALSE]) -
    colMeans(clr_mat[groups == lev[2], , drop = FALSE])
  
  ord <- order(abs(effect), decreasing = TRUE)
  names(effect)[ord][seq_len(min(top_k, length(ord)))]
}

jaccard_index <- function(a, b) {
  a <- unique(a)
  b <- unique(b)
  union_set <- union(a, b)
  
  if (length(union_set) == 0) return(NA_real_)
  
  length(intersect(a, b)) / length(union_set)
}

# -------------------------
# Read data and select OTU columns by position
# -------------------------

tax_otu <- data.frame(read_excel(data_file), check.names = FALSE)

if (!("ID" %in% names(tax_otu))) {
  stop("Column 'ID' was not found in TablaOTUs.xlsx.")
}

Samples <- tax_otu$ID

# OTU columns are selected by position:
# columns 1 and 2 are metadata; the last two columns are non-OTU auxiliary columns.
otu_cols <- 3:(ncol(tax_otu) - 2)

excluded_cols <- setdiff(names(tax_otu), names(tax_otu)[otu_cols])

DF.0 <- tax_otu[, otu_cols, drop = FALSE]

n.OTUs.original <- ncol(DF.0)

taxa <- colnames(DF.0)
taxa.original <- taxa

OTUs <- paste("OTU", 1:n.OTUs.original, sep = "")

colnames(DF.0) <- OTUs

DF.0.total <- DF.0
colnames(DF.0.total) <- OTUs
rownames(DF.0.total) <- Samples

# Convert to numeric
DF.0 <- as.data.frame(lapply(DF.0, function(x) as.numeric(as.character(x))))
DF.0[is.na(DF.0)] <- 0
DF.0 <- as.matrix(DF.0)
rownames(DF.0) <- Samples

DF.0.total <- as.data.frame(lapply(DF.0.total, function(x) as.numeric(as.character(x))))
DF.0.total[is.na(DF.0.total)] <- 0
DF.0.total <- as.matrix(DF.0.total)
rownames(DF.0.total) <- Samples

cat("\nCOLUMN DIAGNOSTIC\n")
```

```
## 
## COLUMN DIAGNOSTIC
```

```
cat("Total columns in Excel:", ncol(tax_otu), "\n")
```

```
## Total columns in Excel: 3090
```

```
cat("OTU columns retained:", ncol(DF.0), "\n")
```

```
## OTU columns retained: 3086
```

```
cat("Non-OTU columns excluded:\n")
```

```
## Non-OTU columns excluded:
```

```
print(excluded_cols)
```

```
## [1] "index"                                                                                                                     
## [2] "ID"                                                                                                                        
## [3] "d__Bacteria;p__Desulfobacterota;c__Desulfuromonadia;o__PB19;f__PB19;g__PB19;s__uncultured_delta"                           
## [4] "d__Bacteria;p__Proteobacteria;c__Alphaproteobacteria;o__Holosporales;f__Holosporaceae;g__uncultured;s__uncultured_organism"
```

```
# -------------------------
# Zero fraction before filtering
# -------------------------

n_initial_taxa <- ncol(DF.0)

zero_before <- zero_summary_row(
  DF.0,
  "Before filtering"
)

# -------------------------
# Remove OTUs with <= 2 reads
# -------------------------

Miseria <- as.vector(which(colSums(DF.0) <= low_count_threshold))

DF.0 <- remove_cols_safe(DF.0, Miseria)
taxa <- taxa[-Miseria]
OTUs <- OTUs[-Miseria]

zero_after_low_count <- zero_summary_row(
  DF.0,
  "After removing OTUs with <= 2 total reads"
)

# -------------------------
# Detect and remove OTUs present in only one sample
# -------------------------

hit <- function(x) {
  min(c(x, 1))
}

DF.0.hits <- as.matrix(DF.0)
DF.0.hits[] <- vapply(DF.0.hits, hit, numeric(1))

singleton_cols <- which(colSums(DF.0.hits) == 1)

DF.0 <- remove_cols_safe(DF.0, singleton_cols)
taxa <- taxa[-singleton_cols]
OTUs <- OTUs[-singleton_cols]

rownames(DF.0) <- Samples

counts_filtered <- DF.0

zero_after_filtering <- zero_summary_row(
  counts_filtered,
  "After filtering, before imputation"
)

# -------------------------
# Filtering summary
# -------------------------

n_final_taxa <- ncol(counts_filtered)

removed_taxa <- n_initial_taxa - n_final_taxa
removed_percent <- 100 * removed_taxa / n_initial_taxa

low_count_percent <- 100 * length(Miseria) / n_initial_taxa
singleton_percent <- 100 * length(singleton_cols) / n_initial_taxa

zero_summary <- rbind(
  zero_before,
  zero_after_low_count,
  zero_after_filtering
)

zero_summary$zero_fraction <- round(zero_summary$zero_fraction, 4)
zero_summary$zero_percent <- round(zero_summary$zero_percent, 2)

write.csv(
  zero_summary,
  "zero_fraction_summary.csv",
  row.names = FALSE
)

cat("\nZERO FRACTION SUMMARY\n")
```

```
## 
## ZERO FRACTION SUMMARY
```

```
print(zero_summary)
```

```
##                                        step samples taxa cells zeros
## 1                          Before filtering      24 3086 74064 41217
## 2 After removing OTUs with <= 2 total reads      24 2599 62376 30225
## 3        After filtering, before imputation      24 2542 61008 28914
##   zero_fraction zero_percent
## 1        0.5565        55.65
## 2        0.4846        48.46
## 3        0.4739        47.39
```

```
cat("\nFILTERING SUMMARY\n")
```

```
## 
## FILTERING SUMMARY
```

```
cat("Initial taxa:", n_initial_taxa, "\n")
```

```
## Initial taxa: 3086
```

```
cat(
  "Taxa with <=", low_count_threshold, "total reads:",
  length(Miseria),
  sprintf("(%.1f%% of initial taxa)\n", low_count_percent)
)
```

```
## Taxa with <= 2 total reads: 487 (15.8% of initial taxa)
```

```
cat(
  "Singleton taxa removed after low-count filtering:",
  length(singleton_cols),
  sprintf("(%.1f%% of initial taxa)\n", singleton_percent)
)
```

```
## Singleton taxa removed after low-count filtering: 57 (1.8% of initial taxa)
```

```
cat("Final taxa:", n_final_taxa, "\n")
```

```
## Final taxa: 2542
```

```
cat("Total taxa removed:", removed_taxa, sprintf("(%.1f%%)\n", removed_percent))
```

```
## Total taxa removed: 544 (17.6%)
```

```
# -------------------------
# Zero replacement sensitivity: GBM vs CZM
# -------------------------

GBM <- impute_safe(counts_filtered, method = "GBM")
CZM <- impute_safe(counts_filtered, method = "CZM")

D_GBM <- aitchison_dist(GBM)
D_CZM <- aitchison_dist(CZM)

mantel_res <- vegan::mantel(
  D_GBM,
  D_CZM,
  method = "spearman",
  permutations = 999
)

sensitivity_summary <- data.frame(
  comparison = "GBM_vs_CZM",
  distance = "Aitchison",
  pearson_correlation = cor(as.vector(D_GBM), as.vector(D_CZM), method = "pearson"),
  spearman_correlation = cor(as.vector(D_GBM), as.vector(D_CZM), method = "spearman"),
  mantel_r = unname(mantel_res$statistic),
  mantel_p = mantel_res$signif,
  mean_absolute_difference = mean(abs(as.vector(D_GBM) - as.vector(D_CZM))),
  median_absolute_difference = median(abs(as.vector(D_GBM) - as.vector(D_CZM))),
  max_absolute_difference = max(abs(as.vector(D_GBM) - as.vector(D_CZM))),
  stringsAsFactors = FALSE
)

# -------------------------
# Optional top-OTU overlap based on clr differences between CAU and CYM
# -------------------------

sample_ids <- rownames(counts_filtered)

if (all(grepl("^(CAU|CYM)", sample_ids))) {
  groups <- sub("^(CAU|CYM).*", "\\1", sample_ids)
  
  sig_GBM <- simple_clr_signature(GBM, groups, top_k = top_k)
  sig_CZM <- simple_clr_signature(CZM, groups, top_k = top_k)
  
  sensitivity_summary$top_k <- top_k
  sensitivity_summary$top_otu_overlap <- length(intersect(sig_GBM, sig_CZM))
  sensitivity_summary$top_otu_jaccard <- jaccard_index(sig_GBM, sig_CZM)
} else {
  sensitivity_summary$top_k <- NA
  sensitivity_summary$top_otu_overlap <- NA
  sensitivity_summary$top_otu_jaccard <- NA
  
  warning(
    "Sample IDs do not all start with CAU or CYM; clr-based top-OTU overlap was skipped."
  )
}

sensitivity_summary$pearson_correlation <- round(
  sensitivity_summary$pearson_correlation, 3
)
sensitivity_summary$spearman_correlation <- round(
  sensitivity_summary$spearman_correlation, 3
)
sensitivity_summary$mantel_r <- round(
  sensitivity_summary$mantel_r, 3
)
sensitivity_summary$mantel_p <- signif(
  sensitivity_summary$mantel_p, 3
)
sensitivity_summary$mean_absolute_difference <- round(
  sensitivity_summary$mean_absolute_difference, 3
)
sensitivity_summary$median_absolute_difference <- round(
  sensitivity_summary$median_absolute_difference, 3
)
sensitivity_summary$max_absolute_difference <- round(
  sensitivity_summary$max_absolute_difference, 3
)

if (!is.na(sensitivity_summary$top_otu_jaccard)) {
  sensitivity_summary$top_otu_jaccard <- round(
    sensitivity_summary$top_otu_jaccard, 3
  )
}

write.csv(
  sensitivity_summary,
  "zero_replacement_sensitivity.csv",
  row.names = FALSE
)

cat("\nZERO-REPLACEMENT SENSITIVITY SUMMARY\n")
```

```
## 
## ZERO-REPLACEMENT SENSITIVITY SUMMARY
```

```
print(sensitivity_summary)
```

```
##   comparison  distance pearson_correlation spearman_correlation mantel_r
## 1 GBM_vs_CZM Aitchison               0.986                0.976    0.976
##   mantel_p mean_absolute_difference median_absolute_difference
## 1    0.001                    4.301                      4.284
##   max_absolute_difference top_k top_otu_overlap top_otu_jaccard
## 1                  10.218   100              78           0.639
```

## 4 Biodiversity

### 4.1 Shannon diversity patterns over time

Shannon diversity indices were computed with the `diversity` function of the `vegan` package and summarized by Species × Treatment × Time. We first visualized mean trajectories (±SE) together with individual observations to inspect temporal patterns and treatment contrasts.

```
library(readr)
library(dplyr)
library(ggplot2)
library(grid)    

datos <- read_table("cell_shanon_data_12_05_25.txt") %>% 
  mutate(
    Species   = factor(Species),
    Time      = factor(Time, levels = c("T0","T1","T2")),
    Treatment = factor(Treatment),
    shanon     = as.numeric(shanon)
  )

summary_data <- datos %>%
  group_by(Species, Treatment, Time) %>%
  summarise(
    mean_shanon = mean(shanon, na.rm = TRUE),
    sd         = sd(shanon,   na.rm = TRUE),
    n          = n(),
    se         = sd / sqrt(n),
    .groups    = "drop"
  )


t1_hw <- summary_data %>% 
  filter(Treatment == "HW", Time == "T1") %>%
  select(Species, y    = mean_shanon)
t2_hw <- summary_data %>% 
  filter(Treatment == "HW", Time == "T2") %>%
  select(Species, yend = mean_shanon)
segments_hw <- left_join(t1_hw, t2_hw, by = "Species")


t12     <- summary_data %>% filter(Time %in% c("T1","T2"))
rango12 <- range(t12$mean_shanon - t12$se, t12$mean_shanon + t12$se)
ymin12  <- rango12[1] - 0.55 * diff(rango12)
ymax12  <- rango12[2] + 0.25 * diff(rango12)


p_d<- ggplot() +
  geom_jitter(
    data  = datos,
    aes(x = Time, y = shanon, color = Species, shape = Treatment),
    width = 0.1, alpha = 0.4, size = 1.2
  ) +
  geom_point(
    data  = summary_data,
    aes(x = Time, y = mean_shanon, color = Species, shape = Treatment),
    size = 3
  ) +
  geom_errorbar(
    data = summary_data,
    aes(x = Time,
        ymin  = mean_shanon - se,
        ymax  = mean_shanon + se,
        color = Species),
    width = 0.2, size = 1.25
  ) +
  scale_color_manual(name   = "Species",
                     values = c("CAU" = "#008ae6", "CYM" = "#ff751a")) +
  scale_shape_manual(name   = "Treatment",
                     values = c("Control" = 16,  # circles filled
                                "HW"      = 17)) +# triangles filled
  scale_linetype_manual(name   = "Treatment",
                        values = c("Control" = "solid",
                                   "HW"      = "dashed")) +
  guides(
    shape    = guide_legend(override.aes = list(size = 3)),
    linetype = guide_legend(override.aes = list(size = 1.5))
  ) +
  labs(
    x        = "Time",
    y        = "Shannon diversity index",
    color    = "Species",
    shape    = "Treatment",
    linetype = "Treatment"
  ) +
  theme_minimal(base_size = 12) +
  theme(
    axis.title.x = element_text(face = "bold"),
    axis.title.y = element_text(face = "bold"),
    axis.text.x  = element_text(face = "bold"),
    legend.title = element_text(face = "bold")
  ) +
  coord_cartesian(ylim = c(ymin12, ymax12)) +
  scale_y_continuous(
    breaks = pretty(c(ymin12, ymax12), n = 5),
    expand = expansion(mult = c(0,0))
  ) 
p_d
```

The plot shows that Caulerpa maintains relatively high and stable diversity across the experiment, with only slight changes between T1 and T2. Cymodocea exhibits a marked rise from T0 to T1, followed by a modest leveling or decline toward T2 under heatwave exposure, while control samples remain comparatively stable. These descriptive trends motivated formal between-species comparisons.

To test whether species differed in Shannon diversity, we applied Wilcoxon rank–sum tests overall (pooling time points) and separately at each sampling time.

```
## Overall (all times + treatments pooled)
bt_overall <- suppressWarnings(stats::wilcox.test(shanon ~ Species, data = datos, exact = FALSE))
bt_overall_p <- bt_overall$p.value

## By time (T0, T1, T2)
bt_by_time <- datos %>%
  group_by(Time) %>%
  summarise(
    med_CAU = median(shanon[Species == "CAU"], na.rm = TRUE),
    med_CYM = median(shanon[Species == "CYM"], na.rm = TRUE),
    p = {
      d <- cur_data()
      suppressWarnings(stats::wilcox.test(d$shanon ~ d$Species, exact = FALSE)$p.value)
    },
    .groups = "drop"
  )

kable(bt_by_time, digits = 3, caption = "Between-species Wilcoxon by time (Shannon)")
```

Table 13: Between-species Wilcoxon by time (Shannon)

| Time | med\_CAU | med\_CYM | p |
| --- | --- | --- | --- |
| T0 | 5.188 | 4.927 | 0.081 |
| T1 | 5.198 | 5.056 | 0.383 |
| T2 | 5.207 | 5.080 | 0.173 |

```
bt_overall_p
```

```
## [1] 0.01300303
```

The overall Wilcoxon test indicated a significant species effect across time points. By-time Wilcoxon tests (unadjusted p-values as printed in the table) suggest a marginal difference at T0, with higher diversity in Caulerpa, whereas differences at T1 and T2 were not statistically significant. Taken together, the figure and tests indicate that Caulerpa generally sustains higher Shannon diversity than Cymodocea, while temporal dynamics—especially the T0→T1 increase in Cymodocea and the slight T1→T2 softening under heatwave—are modest in magnitude.

#### 4.1.1 Acclimation change (T0 → T1) within species

We next examined acclimation effects by directly comparing Shannon diversity between T0 and T1 for each species.

```
acclim <- datos %>%
  group_by(Species) %>%
  summarise(
    med_T0 = median(shanon[Time == "T0"], na.rm = TRUE),
    med_T1 = median(shanon[Time == "T1"], na.rm = TRUE),
    p = {
      x <- shanon[Time == "T0"]; y <- shanon[Time == "T1"]
      if (length(x) > 0 && length(y) > 0)
        suppressWarnings(stats::wilcox.test(x, y, exact = FALSE)$p.value)
      else NA_real_
    },
    direction = case_when(med_T1 > med_T0 ~ "increase",
                          med_T1 < med_T0 ~ "decrease",
                          TRUE            ~ "no_change"),
    .groups = "drop"
  )

kable(acclim, digits = 3, caption = "T0 vs T1 (acclimation) by species")
```

Table 14: T0 vs T1 (acclimation) by species

| Species | med\_T0 | med\_T1 | p | direction |
| --- | --- | --- | --- | --- |
| CAU | 5.188 | 5.198 | 0.663 | increase |
| CYM | 4.927 | 5.056 | 0.383 | increase |

During the acclimation period (T0 → T1), Shannon diversity showed a slight increase in both Caulerpa and Cymodocea sediments (median values rising from 5.19 to 5.20 and from 4.93 to 5.06, respectively). However, these changes were not statistically significant (p = 0.66 for Caulerpa, p = 0.38 for Cymodocea), indicating that although diversity tended to rise during acclimation, the magnitude of the increase was modest and within the range of sampling variability.

#### 4.1.2 Temporal change under Control (T0–T1–T2)

To assess whether Shannon diversity varied over time in the absence of heatwave stress, we applied Kruskal–Wallis tests separately for Caulerpa and Cymodocea under control conditions. This approach evaluates overall differences across the three sampling times (T0, T1, T2) without assuming normality.

```
datos <- read_table("cell_shanon_data_12_05_25_complete.txt") %>% 
  mutate(
    Species   = factor(Species),
    Time      = factor(Time, levels = c("T0","T1","T2")),
    Treatment = factor(Treatment),
    cells     = as.numeric(cells)
  )

kw_ctrl <- datos %>%
  filter(Treatment == "Control") %>%
  group_by(Species) %>%
  summarise(
    kw_p = {
      d <- cur_data()
      suppressWarnings(kruskal.test(shanon ~ Time, data = d)$p.value)
    },
    .groups = "drop"
  )

kable(kw_ctrl, digits = 3, caption = "Kruskal–Wallis test across T0–T1–T2 under control conditions (by species)")
```

Table 15: Kruskal–Wallis test across T0–T1–T2 under control conditions (by species)

| Species | kw\_p |
| --- | --- |
| CAU | 0.733 |
| CYM | 0.432 |

The results (Table) show no significant temporal variation in Shannon diversity under control conditions (p = 0.733 for Caulerpa, p = 0.432 for Cymodocea). This indicates that, in the absence of heatwave exposure, microbial diversity remained relatively stable across the course of the experiment in both host species.

#### 4.1.3 Temporal change under Heatwave (T0–T1–T2)

To evaluate whether heatwave exposure altered the temporal trajectory of Shannon diversity, we applied Kruskal–Wallis tests across the three time points within each species.

```
kw_hw <- datos %>%
  filter(Treatment == "HW") %>%
  group_by(Species) %>%
  summarise(
    kw_p = {
      d <- cur_data()
      suppressWarnings(kruskal.test(shanon ~ Time, data = d)$p.value)
    },
    .groups = "drop"
  )

kable(kw_hw, digits = 3, caption = "Kruskal–Wallis across T0–T1–T2 under heatwave conditions (by species)")
```

Table 16: Kruskal–Wallis across T0–T1–T2 under heatwave conditions (by species)

| Species | kw\_p |
| --- | --- |
| CAU | 0.875 |
| CYM | 0.288 |

Under heatwave conditions, no significant temporal changes were detected in either species (Caulerpa: p = 0.875; Cymodocea: p = 0.288). These results indicate that, while the figure suggests a slight T1→T2 softening in Cymodocea under heatwave exposure, the magnitude of the shift was insufficient to reach statistical significance. Combined with the control analysis, this supports the view that Shannon diversity remained broadly stable over time in both species, with only modest, non-significant fluctuations under heat stress.

#### 4.1.4 Caulerpa control averages between T1 and T2

To probe short-interval changes under control conditions, we compared Shannon diversity between T1 and T2 for Caulerpa using descriptive summaries and a Wilcoxon test as a robust alternative to the Welch t-test.

```
target_var <- "shanon"

dat <- datos %>%
  filter(Species == "CAU", Treatment == "Control", Time %in% c("T1","T2")) %>%
  select(ID = dplyr::any_of("ID"), Time, value = all_of(target_var)) %>%
  filter(!is.na(value))

summary_tbl <- dat %>%
  group_by(Time) %>%
  summarise(
    n    = dplyr::n(),
    mean = mean(value),
    sd   = sd(value),
    se   = sd/sqrt(n),
    .groups = "drop"
  )

knitr::kable(summary_tbl, digits = 3,
             caption = "Caulerpa — Control: comparison of Shannon diversity between T1 and T2 (mean, SD, SE, n)")
```

Table 17: Caulerpa — Control: comparison of Shannon diversity between T1 and T2 (mean, SD, SE, n)

| Time | n | mean | sd | se |
| --- | --- | --- | --- | --- |
| T1 | 3 | 5.202 | 0.075 | 0.043 |
| T2 | 3 | 5.195 | 0.047 | 0.027 |

```
tt_welch <- t.test(value ~ Time, data = dat, var.equal = FALSE)
tt_welch
```

```
## 
##  Welch Two Sample t-test
## 
## data:  value by Time
## t = 0.13793, df = 3.353, p-value = 0.8982
## alternative hypothesis: true difference in means between group T1 and group T2 is not equal to 0
## 95 percent confidence interval:
##  -0.1453022  0.1593022
## sample estimates:
## mean in group T1 mean in group T2 
##         5.201667         5.194667
```

```
w_test <- stats::wilcox.test(value ~ Time, data = dat, exact = FALSE)
w_test
```

```
## 
##  Wilcoxon rank sum test with continuity correction
## 
## data:  value by Time
## W = 5, p-value = 1
## alternative hypothesis: true location shift is not equal to 0
```

The summary table shows nearly identical means at T1 and T2 (5.202 vs 5.195), with small standard errors. Welch’s test confirmed no evidence of a difference (t = 0.138, df = 3.35, p = 0.898; 95% CI for the mean difference: −0.145 to 0.159). The Wilcoxon test led to the same conclusion, showing no evidence of a difference between time points (p = 1.00). Given the very small sample size (n = 3 per time), these findings should be interpreted as compatible with negligible change rather than proof of no effect. Thus, Shannon diversity in Caulerpa remained stable between T1 and T2 under control conditions.

#### 4.1.5 Caulerpa heatwave averages between T1 and T2

To check short-interval changes under heatwave conditions, we compared Shannon diversity between T1 and T2 for Caulerpa using descriptive summaries and a Wilcoxon test as a robust alternative to the Welch t-test.

```
dat <- datos %>%
  filter(Species == "CAU", Treatment == "HW", Time %in% c("T1","T2")) %>%
  select(ID = dplyr::any_of("ID"), Time, value = all_of(target_var)) %>%
  filter(!is.na(value))

summary_tbl <- dat %>%
  group_by(Time) %>%
  summarise(
    n    = dplyr::n(),
    mean = mean(value),
    sd   = sd(value),
    se   = sd/sqrt(n),
    .groups = "drop"
  )

knitr::kable(summary_tbl, digits = 3,
             caption = "Caulerpa – HW: T1 vs T2 (mean, SD, n)")
```

Table 18: Caulerpa – HW: T1 vs T2 (mean, SD, n)

| Time | n | mean | sd | se |
| --- | --- | --- | --- | --- |
| T1 | 3 | 5.202 | 0.075 | 0.043 |
| T2 | 3 | 5.180 | 0.139 | 0.080 |

```
tt_welch <- t.test(value ~ Time, data = dat, var.equal = FALSE)
tt_welch
```

```
## 
##  Welch Two Sample t-test
## 
## data:  value by Time
## t = 0.23467, df = 3.0672, p-value = 0.8293
## alternative hypothesis: true difference in means between group T1 and group T2 is not equal to 0
## 95 percent confidence interval:
##  -0.2644285  0.3070952
## sample estimates:
## mean in group T1 mean in group T2 
##         5.201667         5.180333
```

```
w_test <- stats::wilcox.test(value ~ Time, data = dat, exact = FALSE)
w_test
```

```
## 
##  Wilcoxon rank sum test with continuity correction
## 
## data:  value by Time
## W = 4, p-value = 1
## alternative hypothesis: true location shift is not equal to 0
```

The summary table shows very similar means at T1 and T2 (5.202 vs 5.180) with small standard errors. Both Welch’s test (t = 0.235, df = 3.07, p = 0.829; 95% CI for the mean difference: –0.264 to 0.308) and the Wilcoxon rank-sum test (W = 4, p = 1.00) indicated no evidence of a difference between the two time points. Thus, under heatwave conditions, Caulerpa diversity remained essentially stable between T1 and T2. Given the small sample size (n = 3 per time), these results should be interpreted as consistent with a negligible effect rather than proof of no effect.

#### 4.1.6 Cymodocea control averages between T1 and T2

```
dat <- datos %>%
  filter(Species == "CYM", Treatment == "Control", Time %in% c("T1","T2")) %>%
  select(ID = dplyr::any_of("ID"), Time, value = all_of(target_var)) %>%
  filter(!is.na(value))

summary_tbl <- dat %>%
  group_by(Time) %>%
  summarise(
    n    = dplyr::n(),
    mean = mean(value),
    sd   = sd(value),
    se   = sd/sqrt(n),
    .groups = "drop"
  )

knitr::kable(summary_tbl, digits = 3,
             caption = "Cymodocea – Control: T1 vs T2 (mean, SD, n)")
```

Table 19: Cymodocea – Control: T1 vs T2 (mean, SD, n)

| Time | n | mean | sd | se |
| --- | --- | --- | --- | --- |
| T1 | 3 | 5.073 | 0.165 | 0.095 |
| T2 | 3 | 5.084 | 0.203 | 0.117 |

```
tt_welch <- t.test(value ~ Time, data = dat, var.equal = FALSE)
tt_welch
```

```
## 
##  Welch Two Sample t-test
## 
## data:  value by Time
## t = -0.077316, df = 3.8365, p-value = 0.9422
## alternative hypothesis: true difference in means between group T1 and group T2 is not equal to 0
## 95 percent confidence interval:
##  -0.4377554  0.4144221
## sample estimates:
## mean in group T1 mean in group T2 
##         5.072667         5.084333
```

```
w_test <- stats::wilcox.test(value ~ Time, data = dat, exact = FALSE)
w_test
```

```
## 
##  Wilcoxon rank sum test with continuity correction
## 
## data:  value by Time
## W = 4.5, p-value = 1
## alternative hypothesis: true location shift is not equal to 0
```

The summary statistics indicate that Cymodocea maintained very similar Shannon diversity values between T1 (mean = 5.073, SE = 0.095) and T2 (mean = 5.084, SE = 0.117). Both Welch’s two-sample t-test (t = –0.077, df = 3.84, p = 0.942) and the Wilcoxon rank-sum test (W = 5, p = 1.00) confirmed the absence of significant differences between the two time points. These findings suggest that under control conditions, Cymodocea diversity remained stable across the short interval from T1 to T2, with no detectable changes despite minor variation in the group means.

#### 4.1.7 Cymodocea heatwave averages between T1 and T2

```
dat <- datos %>%
  filter(Species == "CYM", Treatment == "HW", Time %in% c("T1","T2")) %>%
  select(ID = dplyr::any_of("ID"), Time, value = all_of(target_var)) %>%
  filter(!is.na(value))

summary_tbl <- dat %>%
  group_by(Time) %>%
  summarise(
    n    = dplyr::n(),
    mean = mean(value),
    sd   = sd(value),
    se   = sd/sqrt(n),
    .groups = "drop"
  )

knitr::kable(summary_tbl, digits = 3,
             caption = "Cymodocea – HW: T1 vs T2 (mean, SD, n)")
```

Table 20: Cymodocea – HW: T1 vs T2 (mean, SD, n)

| Time | n | mean | sd | se |
| --- | --- | --- | --- | --- |
| T1 | 3 | 5.073 | 0.165 | 0.095 |
| T2 | 3 | 5.056 | 0.106 | 0.061 |

```
tt_welch <- t.test(value ~ Time, data = dat, var.equal = FALSE)
tt_welch
```

```
## 
##  Welch Two Sample t-test
## 
## data:  value by Time
## t = 0.14762, df = 3.4062, p-value = 0.891
## alternative hypothesis: true difference in means between group T1 and group T2 is not equal to 0
## 95 percent confidence interval:
##  -0.3195780  0.3529113
## sample estimates:
## mean in group T1 mean in group T2 
##         5.072667         5.056000
```

```
w_test <- stats::wilcox.test(value ~ Time, data = dat, exact = FALSE)
w_test
```

```
## 
##  Wilcoxon rank sum test with continuity correction
## 
## data:  value by Time
## W = 4, p-value = 1
## alternative hypothesis: true location shift is not equal to 0
```

The descriptive statistics for Cymodocea under heatwave conditions show nearly identical Shannon diversity means at T1 (5.073, SE = 0.095) and T2 (5.056, SE = 0.061). Welch’s two-sample t-test found no evidence of a difference (t = 0.148, df = 3.41, p = 0.891; 95% CI: –0.320 to 0.352), and the Wilcoxon rank-sum test likewise indicated no shift in location (W = 5, p = 1.00). These results indicate that Shannon diversity in Cymodocea remained stable between T1 and T2 under heatwave conditions, with no detectable short-term change.

### 4.2 Taxa biodiversity analysis

```
Datos.Muestras=data.frame(read_excel("TablaOTUs.xlsx"))
Samples.original=Datos.Muestras$ID 
DF.0.original=Datos.Muestras[,-c(1,2)]
row.names(DF.0.original)=Samples.original
OTUs=paste("OTU",1:dim(DF.0.original)[2],sep="")
names(DF.0.original)=OTUs

Sizes=rowSums(DF.0.original)

Tipos.original=as.factor(rep(c("CAU_T0","CAU_T1","CAU_T2C", "CAU_T2HW","CYM_T0","CYM_T1", "CYM_T2C","CYM_T2HW"),each=3))
```

#### 4.2.1 Comparison of Shannon indices of sample types

We quantified the biodiversity of each sample type (CAU\_T\* or CYM\_T\*) using the Shannon index computed from the merged sample formed by combining the three replicates of that type. To test which pairs of sample types differed significantly in Shannon diversity, we used the Hutcheson t-test, implemented in the `Hutcheson_t_test` function of the `ecolTest` package, and adjusted p-values using the Holm method.

```
# Shannon index
SH=function(x){
  vegan::diversity(x,index = "shannon")
}

p.lnp2=function(p){
  if (p==0){return(0)}
  else {
    return(p*(log(p)^2))
  }
}

# Hutcheson variance estimator
Var.SH=function(x){
N=sum(x)
y=sapply(x/sum(x),FUN=p.lnp2)
bit=sapply(x,FUN=function(x){min(x,1)})
S=sum(bit)
vv=(sum(y)-SH(x)^2)/N+(S-1)/(2*N^2)
return(vv)
}

# Standard deviation estimator
sd.SH=function(x){
  sqrt(Var.SH(x))
}
```

```
Samples=Samples.original
DF.0=DF.0.original
Tipos=Tipos.original
DF.0.agrup=aggregate(DF.0,by=list(Tipos),FUN=sum)[,-1]
```

For each sample, we computed its Shannon index and the estimated standard deviation (SD).

```
Resultados=data.frame(Samples,
round(apply(DF.0,MARGIN=1,SH),4),
round(apply(DF.0,MARGIN=1,sd.SH),4))
row.names(Resultados)=NULL
names(Resultados)=c("Samples","SH","sd")

flextable(Resultados)|>
   fontsize(size = 8, part = "all")|>
 width(width = 0.9)
```

| Samples | SH | sd |
| --- | --- | --- |
| CAU\_T0\_1 | 5.2297 | 0.0099 |
| CAU\_T0\_2 | 5.0583 | 0.0083 |
| CAU\_T0\_3 | 5.1884 | 0.0108 |
| CAU\_T1\_1 | 5.2781 | 0.0098 |
| CAU\_T1\_2 | 5.1297 | 0.0099 |
| CAU\_T1\_3 | 5.1981 | 0.0104 |
| CAU\_T2C\_1 | 5.1477 | 0.0102 |
| CAU\_T2C\_2 | 5.1980 | 0.0096 |
| CAU\_T2C\_3 | 5.2405 | 0.0109 |
| CAU\_T2HW\_1 | 5.2974 | 0.0072 |
| CAU\_T2HW\_2 | 5.0278 | 0.0085 |
| CAU\_T2HW\_3 | 5.2167 | 0.0078 |
| CYM\_T0\_1 | 4.8018 | 0.0083 |
| CYM\_T0\_2 | 4.9936 | 0.0098 |
| CYM\_T0\_3 | 4.9272 | 0.0081 |
| CYM\_T1\_1 | 4.9171 | 0.0052 |
| CYM\_T1\_2 | 5.0564 | 0.0056 |
| CYM\_T1\_3 | 5.2460 | 0.0065 |
| CYM\_T2C\_1 | 5.3003 | 0.0063 |
| CYM\_T2C\_2 | 5.0557 | 0.0063 |
| CYM\_T2C\_3 | 4.8975 | 0.0066 |
| CYM\_T2HW\_1 | 4.9353 | 0.0067 |
| CYM\_T2HW\_2 | 5.1045 | 0.0064 |
| CYM\_T2HW\_3 | 5.1299 | 0.0066 |

The next graphic depicts, for each sample, its Shannon index \(SH\) and the error bar \(SH\pm 2\cdot sd\).

```
row.names(Resultados)=Samples

plot(0.1*(1:24),Resultados$SH,
     col=rep(c(rep("green",3),rep("blue",3),rep("purple",3),rep("red",3)),2), 
     pch=c(rep(20,12),rep(18,12)),cex=1,
     xlab="",ylab="Shannon index",xaxt='n',
     main="All samples")
legend("bottomleft",col=c("black","black","green","blue","purple","red"),
       pch=c(20,18,NA,NA,NA,NA),
       lty=c(NA,NA,1,1,1,1),
       legend=c("CAU","CYM","T0","T1","T2C","T2HW"),
       seg.len=0.5,cex=0.5)
segments(0.1*(1:24),Resultados$SH-2*Resultados$sd,
         0.1*(1:24),Resultados$SH+2*Resultados$sd,
         col=rep(c(rep("green",3),rep("blue",3),rep("purple",3),rep("red",3)),2),lwd=1.5)
```

We repeated the process for sample types by merging the three samples of each type into a single composite sample. We report the intervals (SH-3·sd,SH+3·sd). The reason for the factor 3 is that there are 36 pairs of samples to compare, and hence we use as factor for sd the 0.95^1/36^ quantile of the standard normal distribution. In this way, disjoint intervals give statistical evidence (at the 5% significance level) that the corresponding two indices are different.

```
Resultados.agrupados=data.frame(levels(Tipos),
round(apply(DF.0.agrup,MARGIN=1,SH),4),
round(apply(DF.0.agrup,MARGIN=1,sd.SH),4),
round(apply(DF.0.agrup,MARGIN=1,SH)-3*apply(DF.0.agrup,MARGIN=1,sd.SH),4),
round(apply(DF.0.agrup,MARGIN=1,SH)+3*apply(DF.0.agrup,MARGIN=1,sd.SH),4)
)
row.names(Resultados.agrupados)=NULL
names(Resultados.agrupados)=c("Samples","SH","sd","SH-3sd","SH+3sd")

flextable(Resultados.agrupados)|>
   fontsize(size = 8, part = "all")|>
 width(width = 0.9)
```

| Samples | SH | sd | SH-3sd | SH+3sd |
| --- | --- | --- | --- | --- |
| CAU\_T0 | 5.2009 | 0.0056 | 5.1842 | 5.2176 |
| CAU\_T1 | 5.2607 | 0.0059 | 5.2428 | 5.2785 |
| CAU\_T2C | 5.2590 | 0.0060 | 5.2410 | 5.2770 |
| CAU\_T2HW | 5.2457 | 0.0046 | 5.2319 | 5.2594 |
| CYM\_T0 | 4.9321 | 0.0051 | 4.9168 | 4.9474 |
| CYM\_T1 | 5.1190 | 0.0034 | 5.1089 | 5.1291 |
| CYM\_T2C | 5.1226 | 0.0038 | 5.1113 | 5.1338 |
| CYM\_T2HW | 5.0875 | 0.0039 | 5.0759 | 5.0991 |

```
plot(0.1*(1:8),Resultados.agrupados$SH,col=rep(c("green","blue","purple","red"),2),
     pch=c(rep(20,4),rep(18,4)),
     xlab="",ylab="Shannon index",xaxt='n',
     main="All sample types")
segments(0.1*(1:8),Resultados.agrupados$SH-3*Resultados.agrupados$sd,
         0.1*(1:8),Resultados.agrupados$SH+3*Resultados.agrupados$sd,
         col=rep(c("green","blue","purple","red"),2),lwd=1.5)
legend("bottomleft",col=c("black","black","green","blue","purple","red"),
       pch=c(20,18,NA,NA,NA,NA),
       lty=c(NA,NA,1,1,1,1),
      legend=c("CAU","CYM","T0","T1","T2C","T2HW"),
       seg.len=0.5,cex=0.5)
```

SH indices of CAU samples are significantly different from (and actually significantly larger than) those of CYM samples.

```
plot(0.1*(1:4),Resultados.agrupados$SH[1:4],col=c("green","blue","purple","red"),
     pch=20,main="CAU sample types",
     xlab="",ylab="Shannon index",xaxt='n',
     ylim=c(min(Resultados.agrupados$SH[1:4]-3*Resultados.agrupados$sd[1:4]),
              max(Resultados.agrupados$SH[1:4]+3*Resultados.agrupados$sd[1:4])))
segments(0.1*(1:4),Resultados.agrupados$SH[1:4]-3*Resultados.agrupados$sd[1:4],
         0.1*(1:4),Resultados.agrupados$SH[1:4]+3*Resultados.agrupados$sd[1:4],
         col=c("green","blue","purple","red"),lwd=1.5)
legend("topright",col=c("green","blue","purple","red"),
       pch=20,legend=c("T0","T1","T2C","T2HW"),seg.len=0.5,cex=0.5)
```

Only the SH index of T0 samples seems to be significantly different from the others.

```
plot(0.1*(1:4),Resultados.agrupados$SH[5:8],col=c("green","blue","purple","red"),
     pch=18,main="CYM sample types",
     xlab="",ylab="Shannon index",xaxt='n',          ylim=c(min(Resultados.agrupados$SH[5:8]-3*Resultados.agrupados$sd[5:8]),max(Resultados.agrupados$SH[5:8]+3*Resultados.agrupados$sd[5:8])))
segments(0.1*(1:4),Resultados.agrupados$SH[5:8]-3*Resultados.agrupados$sd[5:8],
         0.1*(1:4),Resultados.agrupados$SH[5:8]+3*Resultados.agrupados$sd[5:8],
         col=c("green","blue","purple","red"),lwd=1.5)
legend("topleft",col=c("green","blue","purple","red"),
       pch=18,legend=c("T0","T1","T2C","T2HW"),seg.len=0.5,cex=0.5)
```

All four SH indices are significantly different, except for T1 and T2C.

We then used the Hutcheson t-test to assess whether Shannon indices differed significantly. We perform pairwise comparisons and adjust the p-values using the Holm method. The conclusions are the same.

```
pvalSH.G=matrix(NA,nrow=8,ncol=8)
for (i in 1:7){
  for (j in (i+1):8){
   pvalSH.G[i,j]= Hutcheson_t_test(DF.0.agrup[i,],DF.0.agrup[j,])$p.value
  }
}
pvalSH.G=matrix(p.adjust(pvalSH.G,method="holm"),nrow=8)

colnames(pvalSH.G)=levels(Tipos)
row.names(pvalSH.G)=levels(Tipos)

flextable(data.frame(round(pvalSH.G,6)))|>
   fontsize(size = 8, part = "all")|>
 width(width = 0.9)
```

| CAU\_T0 | CAU\_T1 | CAU\_T2C | CAU\_T2HW | CYM\_T0 | CYM\_T1 | CYM\_T2C | CYM\_T2HW |
| --- | --- | --- | --- | --- | --- | --- | --- |
|  | 0 | 0.000000 | 0.000000 | 0 | 0 | 0.000000 | 0 |
|  |  | 0.950346 | 0.179925 | 0 | 0 | 0.000000 | 0 |
|  |  |  | 0.228577 | 0 | 0 | 0.000000 | 0 |
|  |  |  |  | 0 | 0 | 0.000000 | 0 |
|  |  |  |  |  | 0 | 0.000000 | 0 |
|  |  |  |  |  |  | 0.950346 | 0 |
|  |  |  |  |  |  |  | 0 |
|  |  |  |  |  |  |  |  |

#### 4.2.2 Behaviour of Shannon indices under thresholds

We address the following question: If we set a threshold \(p\_0\), we only retain from each sample the OTUs that appear with a relative frequency \(\geqslant p\_0\) and calculate the Shannon index (SH) of those samples, how does its longitudinal behavior vary across the sequence T0, T1, T2C, and T2HW as \(p\_0\) increases?

We have used the following method to answer this question:

- We consider \(p\_0\) thresholds from 0.00005 (0.005%) to 0.05 (5%), advancing in steps of 0.00005.
- For each threshold, in each sample, we retain the OTUs whose relative frequency is \(\geqslant p\_0\)
- Then, we group the samples by sample type and compute the SH of the grouped samples.
- We visually analyze the trend in SH among CAU and CYM samples as \(p\_0\) increases.
- We also compute, for each type of sample and for relevant thresholds, the proportion of OTUs present in **at least one** sample of that type whose relative frequency in all three samples of that type is greater than or equal to the threshold.

We obtain that, in the CAU samples, at some point between the 0.15% and 0.2% thresholds, and in the CYM samples, at some point between the 0.1% and 0.15% thresholds, the longitudinal behavior of the SH changes for the first time from that observed in the whole samples, shifting from a rise-fall-fall pattern to a rise-fall-rise pattern.

For each type of CAU sample, around 12-13% of OTUs present in at least one sample of this type have relative frequency in all three samples of that type greater than or equal to 0.1%. For CYM sample types, this figure descends to 9-10%.

```
# threshold
llindar=function(x,l){
if (x>=l){return(1)}
  else{return(0)}
}
```

```
#Global sample
DF.0=as.matrix(DF.0)
DF.0.prop=DF.0/rowSums(DF.0)
DF.0.agrup=as.matrix(DF.0.agrup)
DF.0.agrup.prop=DF.0.agrup/rowSums(DF.0.agrup)
```

Original sample sizes and range of proportions within them:

```
Tamaños=rowSums(DF.0)
Props.min=round(apply(DF.0.prop,MARGIN=1,function(x){min(x[x>0])}),6)
Props.max=apply(DF.0.prop,MARGIN=1,function(x){max(x[x>0])})
Info=data.frame(Tamaños,Props.min,Props.max)
names(Info)=c("Size","Smallest prop.","Largest prop.")

flextable(Info)|>
   fontsize(size = 8, part = "all")|>
 width(width = 0.9)
```

| Size | Smallest prop. | Largest prop. |
| --- | --- | --- |
| 32452 | 0.000031 | 0.05805497 |
| 45161 | 0.000022 | 0.05832466 |
| 26298 | 0.000038 | 0.05354019 |
| 31355 | 0.000032 | 0.05243183 |
| 30592 | 0.000033 | 0.05458944 |
| 29368 | 0.000034 | 0.07147235 |
| 30554 | 0.000033 | 0.05194083 |
| 33119 | 0.000030 | 0.05694616 |
| 26568 | 0.000038 | 0.06884222 |
| 62616 | 0.000016 | 0.06188514 |
| 43726 | 0.000023 | 0.06026163 |
| 50715 | 0.000020 | 0.05223307 |
| 57062 | 0.000018 | 0.10017174 |
| 38684 | 0.000026 | 0.09365629 |
| 56482 | 0.000018 | 0.09686272 |
| 145523 | 0.000007 | 0.11633900 |
| 122687 | 0.000008 | 0.08515980 |
| 82489 | 0.000012 | 0.08112597 |
| 91168 | 0.000011 | 0.06932257 |
| 97111 | 0.000010 | 0.09009278 |
| 97125 | 0.000010 | 0.12887516 |
| 88157 | 0.000011 | 0.14189457 |
| 94560 | 0.000011 | 0.11748096 |
| 89922 | 0.000011 | 0.11695692 |

##### 4.2.2.1 Original sample

```
Resultados.Agrupados=data.frame(
  SH=apply(DF.0.agrup,MARGIN=1,SH),
  Varianza=apply(DF.0.agrup,MARGIN=1,Var.SH))
```

```
plot(0.1*(1:8),Resultados.Agrupados$SH,col=rep(c("green","blue","purple","red"),2),
     pch=c(rep(20,4),rep(18,4)),
     xlab="",ylab="SH",xaxt='n',
     main="Grouped samples")
segments(0.1*(1:8),Resultados.Agrupados$SH-3*sqrt(Resultados.Agrupados$Varianza),
         0.1*(1:8),Resultados.Agrupados$SH+3*sqrt(Resultados.Agrupados$Varianza),
         col=rep(c("green","blue","purple","red"),2),lwd=1.5)
legend("topright",col=c("black","black","green","blue","purple","red"),
       pch=c(20,18,NA,NA,NA,NA),
       lty=c(NA,NA,1,1,1,1),
      legend=c("CAU","CYM","T0","T1","T2C","T2HW"),
       seg.len=0.5,cex=0.5)
```

```
plot(0.1*(1:4),Resultados.Agrupados$SH[1:4],col=c("green","blue","purple","red"),
     pch=20,main="Grouped samples: CAU",
     xlab="",ylab="SH",xaxt='n',
     ylim=c(min(Resultados.Agrupados$SH[1:4]-3*sqrt(Resultados.Agrupados$Varianza[1:4])),
              max(Resultados.Agrupados$SH[1:4]+3*sqrt(Resultados.Agrupados$Varianza[1:4]))))
segments(0.1*(1:4),Resultados.Agrupados$SH[1:4]-3*sqrt(Resultados.Agrupados$Varianza[1:4]),
         0.1*(1:4),Resultados.Agrupados$SH[1:4]+3*sqrt(Resultados.Agrupados$Varianza[1:4]),
         col=c("green","blue","purple","red"),lwd=1.5)
legend("topleft",col=c("green","blue","purple","red"),
       pch=20,legend=c("T0","T1","T2C","T2HW"),cex=0.5)
```

```
plot(0.1*(1:4),Resultados.Agrupados$SH[5:8],col=c("green","blue","purple","red"),
     pch=18,main="Grouped samples: CYM",
     xlab="",ylab="SH",xaxt='n',          ylim=c(min(Resultados.Agrupados$SH[5:8]-3*sqrt(Resultados.Agrupados$Varianza[5:8])),max(Resultados.Agrupados$SH[5:8]+3*sqrt(Resultados.Agrupados$Varianza[5:8]))))
segments(0.1*(1:4),Resultados.Agrupados$SH[5:8]-3*sqrt(Resultados.Agrupados$Varianza[5:8]),
         0.1*(1:4),Resultados.Agrupados$SH[5:8]+3*sqrt(Resultados.Agrupados$Varianza[5:8]),
         col=c("green","blue","purple","red"),lwd=1.5)
legend("topleft",col=c("green","blue","purple","red"),
       pch=18,legend=c("T0","T1","T2C","T2HW"),cex=0.5)
```

##### 4.2.2.2 Enter thresholds

```
Resultados=c()
for (i in 1:1000){
print(i)
i0=i*0.00005
llindar.0=function(x){llindar(x,i0)}
DD=vapply(DF.0.prop, llindar.0, numeric(1))*DF.0
DD.agrup=aggregate(DD,by=list(Tipos),FUN=sum)[,-1]
Resultados=rbind(Resultados,
       c(apply(DD.agrup,MARGIN=1,SH),#Shannon
       apply(DD.agrup,MARGIN=1,sd.SH),#Variança
       apply(DD.agrup,MARGIN=1,SH)-3*apply(DD.agrup,MARGIN=1,sd.SH), #LE de l'IC
       apply(DD.agrup,MARGIN=1,SH)+3*apply(DD.agrup,MARGIN=1,sd.SH),#UE  de l'IC
       
       i0 #llindar
       ))
}


Resultados=data.frame(Resultados)

names(Resultados)=c(paste("SH",levels(Tipos),sep="."),
      paste("sd",levels(Tipos),sep="."),
      paste("LE",levels(Tipos),sep="."),
      paste("UE",levels(Tipos),sep="."),
      "threshold")

saveRDS(Resultados, file="Resultados.Shannon.Umbrales.RData")
```

```
p.valores=c()
for (i in 1:1000){
print(i)
i0=i*0.00005
llindar.0=function(x){llindar(x,i0)}
DD=vapply(DF.0.prop, llindar.0, numeric(1))*DF.0
DD.agrup=aggregate(DD,by=list(Tipos),FUN=sum)[,-1]


pvalSH.G=matrix(NA,nrow=8,ncol=8)
for (i in 1:7){
  for (j in (i+1):8){
   pvalSH.G[i,j]= Hutcheson_t_test(DD.agrup[i,],DD.agrup[j,])$p.value
  }
}
pvalSH.G=p.adjust(pvalSH.G,method="holm")

p.valores=rbind(p.valores,
                c(i0,
                  pvalSH.G))

}

write.csv(p.valores, file="p.valores.Shannon.Umbrales.csv",row.names=FALSE)
```

The results are in the file “Resultados.Shannon.Umbrales.RData”. Its variables are (for each type of sample: CAU\_T0, CAU\_T1 etc.):

- *SH.type*: The Shannon index (SH) of the union of the three samples of that type
- *sd.type*: The estimated standard deviation of the SH of the union of the three samples of that type
- *LE.type*: SH minus 3 times the estimated standard deviation
- *UE.type*: SH plus 3 times the estimated standard deviation
- *Threshold*: The corresponding threshold

We show its first 10 rows (rounded, and splitted into different tables, for horizontal space reasons), corresponding to thresholds from 0.00005 to 0.0005. :

```
Resultados=readRDS("Resultados.Shannon.Umbrales.RData")
names(Resultados)=c(paste("SH",levels(Tipos),sep="."),
      paste("sd",levels(Tipos),sep="."),
      paste("LE",levels(Tipos),sep="."),
      paste("UE",levels(Tipos),sep="."),
      "Threshold")

Resultados.r4=Resultados
Resultados.r4[,1:32]=round(Resultados.r4[,1:32],4)

flextable(Resultados.r4[1:10,c(33,1+8*(0:3),2+8*(0:3))])|>
   fontsize(size = 8, part = "all")|>
 width(width = 0.9)
```

| Threshold | SH.CAU\_T0 | sd.CAU\_T0 | LE.CAU\_T0 | UE.CAU\_T0 | SH.CAU\_T1 | sd.CAU\_T1 | LE.CAU\_T1 | UE.CAU\_T1 |
| --- | --- | --- | --- | --- | --- | --- | --- | --- |
| 0.00005 | 5.1252 | 0.0053 | 5.1092 | 5.1412 | 5.1997 | 0.0058 | 5.1824 | 5.2170 |
| 0.00010 | 5.0481 | 0.0051 | 5.0326 | 5.0635 | 5.1092 | 0.0055 | 5.0926 | 5.1257 |
| 0.00015 | 5.0011 | 0.0050 | 4.9859 | 5.0162 | 5.0479 | 0.0054 | 5.0318 | 5.0641 |
| 0.00020 | 4.9245 | 0.0049 | 4.9098 | 4.9391 | 4.9787 | 0.0052 | 4.9630 | 4.9944 |
| 0.00025 | 4.8751 | 0.0048 | 4.8607 | 4.8895 | 4.9337 | 0.0052 | 4.9182 | 4.9491 |
| 0.00030 | 4.8395 | 0.0047 | 4.8253 | 4.8537 | 4.8735 | 0.0050 | 4.8584 | 4.8887 |
| 0.00035 | 4.7807 | 0.0046 | 4.7668 | 4.7946 | 4.8427 | 0.0050 | 4.8277 | 4.8577 |
| 0.00040 | 4.7456 | 0.0046 | 4.7319 | 4.7594 | 4.8042 | 0.0049 | 4.7893 | 4.8190 |
| 0.00045 | 4.7104 | 0.0045 | 4.6968 | 4.7239 | 4.7588 | 0.0049 | 4.7442 | 4.7733 |
| 0.00050 | 4.6612 | 0.0044 | 4.6479 | 4.6745 | 4.7291 | 0.0048 | 4.7146 | 4.7435 |

```
flextable(Resultados.r4[1:10,c(33,3+8*(0:3),4+8*(0:3))])|>
   fontsize(size = 8, part = "all")|>
 width(width = 0.9)
```

| Threshold | SH.CAU\_T2C | sd.CAU\_T2C | LE.CAU\_T2C | UE.CAU\_T2C | SH.CAU\_T2HW | sd.CAU\_T2HW | LE.CAU\_T2HW | UE.CAU\_T2HW |
| --- | --- | --- | --- | --- | --- | --- | --- | --- |
| 0.00005 | 5.1943 | 0.0058 | 5.1769 | 5.2117 | 5.1539 | 0.0043 | 5.1408 | 5.1669 |
| 0.00010 | 5.1027 | 0.0056 | 5.0860 | 5.1194 | 5.0714 | 0.0042 | 5.0588 | 5.0839 |
| 0.00015 | 5.0536 | 0.0055 | 5.0372 | 5.0699 | 5.0186 | 0.0041 | 5.0063 | 5.0309 |
| 0.00020 | 4.9697 | 0.0053 | 4.9539 | 4.9855 | 4.9529 | 0.0040 | 4.9409 | 4.9649 |
| 0.00025 | 4.9234 | 0.0052 | 4.9078 | 4.9389 | 4.9069 | 0.0039 | 4.8952 | 4.9187 |
| 0.00030 | 4.8896 | 0.0051 | 4.8743 | 4.9050 | 4.8537 | 0.0038 | 4.8422 | 4.8653 |
| 0.00035 | 4.8425 | 0.0050 | 4.8274 | 4.8576 | 4.8159 | 0.0038 | 4.8045 | 4.8274 |
| 0.00040 | 4.8079 | 0.0050 | 4.7929 | 4.8228 | 4.7660 | 0.0037 | 4.7548 | 4.7772 |
| 0.00045 | 4.7851 | 0.0050 | 4.7702 | 4.7999 | 4.7365 | 0.0037 | 4.7254 | 4.7476 |
| 0.00050 | 4.7406 | 0.0049 | 4.7259 | 4.7553 | 4.7040 | 0.0037 | 4.6930 | 4.7150 |

```
flextable(Resultados.r4[1:10,c(33,5+8*(0:3),6+8*(0:3))])|>
   fontsize(size = 8, part = "all")|>
 width(width = 0.9)
```

| Threshold | SH.CYM\_T0 | sd.CYM\_T0 | LE.CYM\_T0 | UE.CYM\_T0 | SH.CYM\_T1 | sd.CYM\_T1 | LE.CYM\_T1 | UE.CYM\_T1 |
| --- | --- | --- | --- | --- | --- | --- | --- | --- |
| 0.00005 | 4.8502 | 0.0049 | 4.8355 | 4.8649 | 5.0004 | 0.0032 | 4.9909 | 5.0100 |
| 0.00010 | 4.7581 | 0.0047 | 4.7440 | 4.7722 | 4.9137 | 0.0031 | 4.9045 | 4.9229 |
| 0.00015 | 4.6853 | 0.0046 | 4.6717 | 4.6990 | 4.8593 | 0.0030 | 4.8503 | 4.8684 |
| 0.00020 | 4.6284 | 0.0045 | 4.6149 | 4.6418 | 4.7987 | 0.0030 | 4.7898 | 4.8075 |
| 0.00025 | 4.5794 | 0.0044 | 4.5662 | 4.5927 | 4.7475 | 0.0029 | 4.7387 | 4.7562 |
| 0.00030 | 4.5353 | 0.0044 | 4.5222 | 4.5483 | 4.7035 | 0.0029 | 4.6948 | 4.7121 |
| 0.00035 | 4.5003 | 0.0043 | 4.4874 | 4.5132 | 4.6659 | 0.0029 | 4.6573 | 4.6745 |
| 0.00040 | 4.4627 | 0.0043 | 4.4499 | 4.4754 | 4.6107 | 0.0028 | 4.6023 | 4.6191 |
| 0.00045 | 4.4162 | 0.0042 | 4.4036 | 4.4288 | 4.5693 | 0.0028 | 4.5609 | 4.5776 |
| 0.00050 | 4.3812 | 0.0042 | 4.3687 | 4.3937 | 4.5383 | 0.0028 | 4.5300 | 4.5466 |

```
flextable(Resultados.r4[1:10,c(33,7+8*(0:3),8+8*(0:3))])|>
   fontsize(size = 8, part = "all")|>
 width(width = 0.9)
```

| Threshold | SH.CYM\_T2C | sd.CYM\_T2C | LE.CYM\_T2C | UE.CYM\_T2C | SH.CYM\_T2HW | sd.CYM\_T2HW | LE.CYM\_T2HW | UE.CYM\_T2HW |
| --- | --- | --- | --- | --- | --- | --- | --- | --- |
| 0.00005 | 5.0126 | 0.0036 | 5.0019 | 5.0233 | 4.9765 | 0.0037 | 4.9655 | 4.9875 |
| 0.00010 | 4.9095 | 0.0034 | 4.8992 | 4.9197 | 4.8922 | 0.0036 | 4.8816 | 4.9029 |
| 0.00015 | 4.8446 | 0.0033 | 4.8346 | 4.8546 | 4.8148 | 0.0035 | 4.8044 | 4.8252 |
| 0.00020 | 4.7855 | 0.0033 | 4.7756 | 4.7953 | 4.7597 | 0.0034 | 4.7494 | 4.7699 |
| 0.00025 | 4.7292 | 0.0032 | 4.7195 | 4.7388 | 4.7049 | 0.0034 | 4.6948 | 4.7150 |
| 0.00030 | 4.6798 | 0.0032 | 4.6702 | 4.6893 | 4.6562 | 0.0033 | 4.6462 | 4.6661 |
| 0.00035 | 4.6419 | 0.0032 | 4.6324 | 4.6513 | 4.6095 | 0.0033 | 4.5997 | 4.6193 |
| 0.00040 | 4.5895 | 0.0031 | 4.5802 | 4.5988 | 4.5639 | 0.0032 | 4.5542 | 4.5736 |
| 0.00045 | 4.5482 | 0.0031 | 4.5390 | 4.5575 | 4.5322 | 0.0032 | 4.5226 | 4.5418 |
| 0.00050 | 4.5203 | 0.0030 | 4.5111 | 4.5294 | 4.5011 | 0.0032 | 4.4915 | 4.5106 |

We start by checking several thresholds:

- 0.0001: OTUs that represent more than 0.01% in their samples
- 0.001: OTUs that represent more than 0.1% in their samples
- 0.0025: OTUs that represent more than 0.25% in their samples
- 0.005: OTUs that represent more than 0.5% in their samples
- 0.0075: OTUs that represent more than 0.75% in their samples
- 0.01: OTUs that represent more than 1% in their samples
- 0.025: OTUs that represent more than 2.5% in their samples

```
Dibu.global=function(p){
  Tall=as.matrix(Resultados[Resultados$Threshold==p,])
plot(0.1*(1:8),Tall[1,1:8],
     col=rep(c("green","blue","purple","red"),2),
     pch=c(rep(20,4),rep(18,4)),
     xlab="",ylab="SH",xaxt='n',main=paste("Threshold",p,sep=" "))
segments(x0=0.1*(1:8),y0=Tall[1,17:24],
         x1=0.1*(1:8),y1=Tall[1,25:32],
         col=rep(c("green","blue","purple","red"),2),lwd=1.5)
legend("topright",col=c("black","black","green","blue","purple","red"),
       pch=c(20,18,NA,NA,NA,NA),
       lty=c(NA,NA,1,1,1,1),
      legend=c("CAU","CYM","T0","T1","T2C","T2HW"),
       seg.len=0.5,cex=0.5)
}
```

```
Dibu.global(0.0001)
```

```
Dibu.global(0.001)
```

```
Dibu.global(0.0025)
```

```
Dibu.global(0.005)
```

```
Dibu.global("0.0075")
```

```
Dibu.global(0.01)
```

```
Dibu.global(0.025)
```

As it can be seen, the longitudinal behavior of Shannon indices changes from p_0_=0.1% (which is similar to the global behaviour) to 0.25%.

Let us see the graphics for thresholds between 0.001 and 0.005:

```
for (i in 0:8){
Dibu.global(0.001+0.0005*i)
}
```

##### 4.2.2.3 How many OTUs are we talking about?

For each type of samples, the next graph represents the proportion of OTUs present in **at least one** sample of this type whose relative frequency in all three samples of that type is greater than or equal to the percentage indicated on the x-axis. The proportions are also given in a table.

```
  n=seq(from=0.001,to=0.003,by=0.0005)
  N=length(n)
Result=n
for (i in 1:8){
 X1=DF.0.prop[3*(i-1)+1,]
 X1=sort(X1[X1!=0])
 X2=DF.0.prop[3*(i-1)+2,]
 X2=sort(X2[X2!=0])
 X3=DF.0.prop[3*(i-1)+3,]
 X3=sort(X3[X3!=0])

  PP=rep(0,N)
  for(j in 1:N){PP[j]=length(X1[X1>=n[j]] & X2[X2>=n[j]] & X3[X3>=n[j]])/length(X1[X1>0] | X2[X2>0] | X3[X3>0])}
  plot(100*n,PP,pch=20,type="l",lwd=1.5,xaxp=c(0.1,0.3,N),yaxp=c(0,0.2,20),xlab="%",ylab="Proportion of OTUs whose percentage is higher than ...",main=Tipos[i])
  abline(v=0.01*(0:100),lwd=0.5,col="red")
  abline(h=0.01*(0:100),lwd=0.5,col="red")
  abline(v=0.15,lwd=1,col="blue")
 Result=cbind(Result,round(PP,4)) 
}
```

```
Result=data.frame(Result)
names(Result)=c("Threshold",levels(Tipos))
row.names(Result)=NULL


flextable(Result)|>
   fontsize(size = 8, part = "all")|>
 width(width = 0.9)
```

| Threshold | CAU\_T0 | CAU\_T1 | CAU\_T2C | CAU\_T2HW | CYM\_T0 | CYM\_T1 | CYM\_T2C | CYM\_T2HW |
| --- | --- | --- | --- | --- | --- | --- | --- | --- |
| 0.0010 | 0.1269 | 0.1283 | 0.1394 | 0.1162 | 0.1027 | 0.0896 | 0.0922 | 0.0901 |
| 0.0015 | 0.0968 | 0.0929 | 0.1004 | 0.0829 | 0.0759 | 0.0662 | 0.0643 | 0.0683 |
| 0.0020 | 0.0751 | 0.0765 | 0.0797 | 0.0652 | 0.0610 | 0.0508 | 0.0539 | 0.0550 |
| 0.0025 | 0.0609 | 0.0650 | 0.0632 | 0.0546 | 0.0521 | 0.0388 | 0.0446 | 0.0459 |
| 0.0030 | 0.0509 | 0.0526 | 0.0563 | 0.0446 | 0.0417 | 0.0342 | 0.0348 | 0.0363 |

### 4.3 Beta diversity: samples divergence analysis

According to the Bray-Curtis distance (correctly applied to compositions), the CAU and CYM samples at T0 are more different than at T1, more similar at T1 than at T2C, and again more similar at T2HW than at T2C (or than at T1 or T0, for that matter). Overall, the global sets of CAU and CYM samples are more different than the starting T0 samples, as if part of the original differences were diluted. Are these differences statistically significant?

To assess the statistical significance of the differences in median distances between two pairs of samples groups, we have devised a method based on a resampling procedure. Using this method, we obtain that these convergences and divergences from T0 to T1, from T1 to T2C, from T2C to T2HW, and from T0 to the global sample, are statistically significant.

```
DF.0.agrup=aggregate(DF.0,by=list(Tipos),FUN=sum)[,-1]
```

The next table shows, for each level T0, T1, T2C, T2HW, and Global (all samples), the median Bray-Curtis (BC) distances between the compositions of the CAU and CYM samples at those levels.

| Times | Average BC-dist. |
| --- | --- |
| T0 | 0.4124 |
| T1 | 0.4097 |
| T2C | 0.4493 |
| T2HW | 0.3766 |
| Global | 0.4189 |

The BC-distances decrease from T0 to T1. Then, from T1 to T2C they increase, while from T1 to T2HW they decrease further. Also, from T0 to Global it decreases. Are these “convergences” and “divergences” of samples statistically significant? We answer this question by means of a contrast with null hypothesis “The CAU\_T1 and CYM\_T1 samples are as similar as the CAU\_T0 and CYM\_T0 samples” and alternative hypothesis “The CAU\_T1 and CYM\_T1 samples are more similar than the CAU\_T0 and CYM\_T0 samples”.

To solve this contrast, we have resorted to a resampling process. The **broad** idea is:

- On the one hand, we merge all 3 samples CAU\_T0 in a single sample, and all 3 samples CYM\_T0 in another single sample, and we repeat 1000 times the following:

  - We split the CAU merged sample into 3 disjoint random samples of the sizes of the original CAU\_T0\_1, CAU\_T0\_2, CAU\_T0\_3 samples forming it
  - We split the CYM merged sample into 3 disjoint random samples of the sizes of the original CYM\_T0\_1, CYM\_T0\_2, CYM\_T0\_3 samples forming it
  - We compute the median distance between the 3 CAU random samples and the 3 CYM random samples

  In this way, we obtain a vector of 1000 median distances between CAU and CYM for T0
- On the other hand, we merge all 6 samples CAU\_T0 and CAU\_T1 in a single sample, and all 6 samples CYM\_T0 and CYM\_T1 in another single sample, and we repeat 1000 times the following:

  - We take from the CAU merged sample 3 disjoint random samples (without reposition) of the sizes of the original CAU\_T0\_1, CAU\_T0\_2, CAU\_T0\_3 samples
  - We take from the CYM merged sample into 3 disjoint random samples (without reposition) of the sizes of the original CYM\_T0\_1, CYM\_T0\_2, CYM\_T0\_3 samples
  - We compute the median distance between the 3 CAU random samples and the 3 CYM random samples

  In this way, we obtain a vector of 1000 median distances between CAU and CYM for the union of T0 and T1

The idea is that if the T1 samples are “as similar” as the T0 samples, the median distances between triples of disjoint random samples from the merged T0 samples should be similar to the median distances between triples of disjoint random samples (of the same sizes) from the merged T0+T1 samples. Conversely, if the T1 samples are “more similar” than the T0 samples, the median distances between triples of random samples from the merged T0 samples should tend to be larger than the median distances between triples of random samples (of the same sizes) from the merged T0+T1 samples.

So, we can use as p-value the proportion of pairs (median distance between CAU and CYM for T0, median distance between CAU and CYM for T0+T1) where the first entry is *smaller* than the second entry. If the T1 samples are “as similar” as the T0 samples, we would expect this to happen half of times, while if the T0 samples are more different than the T1 samples, we would expect this to happen with low frequency.

The problem is that the CYM samples are larger than CAU samples, and it can bias the samples; for instance if we merge CAU\_T1 and CYM\_T1 samples and take random samples, most of the taxa will come from CYM:

| Samples | Sizes |
| --- | --- |
| CAU\_T0\_1 | 32452 |
| CAU\_T0\_2 | 45161 |
| CAU\_T0\_3 | 26298 |
| CAU\_T1\_1 | 31355 |
| CAU\_T1\_2 | 30592 |
| CAU\_T1\_3 | 29368 |
| CYM\_T0\_1 | 57062 |
| CYM\_T0\_2 | 38684 |
| CYM\_T0\_3 | 56482 |
| CYM\_T1\_1 | 145523 |
| CYM\_T1\_2 | 122687 |
| CYM\_T1\_3 | 82489 |

To solve this drawback, and since we apply BC-distances to proportions, *we reescale all samples to size 10^6^ (up to rounding; we need integer frequencies for the simulations) before merging*.

```
DF=round(DF.0.prop*10^6)
DF.prop=DF/rowSums(DF)
DF.agrup=aggregate(DF,by=list(Tipos),FUN=sum)[,-1]
Sizes=rowSums(DF)
```

The new sample sizes are

| Samples | Sizes |
| --- | --- |
| CAU\_T0\_1 | 1000070 |
| CAU\_T0\_2 | 999907 |
| CAU\_T0\_3 | 999911 |
| CAU\_T1\_1 | 1000070 |
| CAU\_T1\_2 | 1000030 |
| CAU\_T1\_3 | 999912 |
| CYM\_T0\_1 | 1000234 |
| CYM\_T0\_2 | 1000111 |
| CYM\_T0\_3 | 1000043 |
| CYM\_T1\_1 | 1000078 |
| CYM\_T1\_2 | 999876 |
| CYM\_T1\_3 | 999852 |

Let us check first that with these reescaled samples (where, due to rounding, the proportions are not exactly the original ones but very close to them), the behaviour of the distances is the same as the original one:

| Times | Average BC-dist. |
| --- | --- |
| T0 | 0.4124 |
| T1 | 0.4097 |
| T2C | 0.4493 |
| T2HW | 0.3766 |
| Global | 0.4189 |

No change.

We shall use the following function:

- Function `Simulation`
  - takes the different samples CAU y CYM at the specified times, for instance, CAU\_T0, CAU\_T1, CYM\_T0 and CYM\_T1, or simply CAU\_T0 and CYM\_T0
  - considers as “base” samples the samples CAU and CYM at the first of each set of times, for instance CAU\_T0 and CYM\_T0
  - merges all samples CAU in one sample, and all samples CYM in another sample
  - it extracts, from the CAU merged sample, 3 disjoint random samples (without reposition) of the same sizes as the reescaled CAU base samples, and from the CYM merged sample, 3 disjoint random samples of the same sizes as the reescaled CYM base samples
  - it computes the median specified distance between the 3 CAU random samples and the 3 CYM random samples

```
#' II indexes of the CAU grouped samples
#' The first one is the "base" time
#' Example: CAU T0+T1 and CYM T0+T1 vs CAU T0 and CYM T0
#' II=c(1,2)
#' Example: CAU_T0 vs CYM_T0
#' II=c(1)
Simulation=function(II,distancia){
#CAU
XX1=c()
for (i in 1:length(II)){
  XX1=c(XX1,rep(OTUs,DF.agrup[II[i],]))
}
XX1=factor(XX1,levels=OTUs)
ind1=1:length(XX1)
#CYM
XX2=c()
for (i in 1:length(II)){
  XX2=c(XX2,rep(OTUs,DF.agrup[4+II[i],]))
}
XX2=factor(XX2,levels=OTUs)
ind2=1:length(XX2)

# sizes of base samples
SS=Sizes[c(3*(II[1]-1)+1:3,3*((4+II[1])-1)+1:3)]


ind1.1=sample(ind1,SS[1],replace=FALSE)
ind1.2=sample(ind1[-ind1.1],SS[2],replace=FALSE)
ind1.3=sample(ind1[-c(ind1.1,ind1.2)],SS[3],replace=FALSE)

ind2.1=sample(ind2,SS[4],replace=FALSE)
ind2.2=sample(ind2[-ind2.1],SS[5],replace=FALSE)
ind2.3=sample(ind2[-c(ind2.1,ind1.2)],SS[6],replace=FALSE)

Y1.1=prop.table(table(factor(XX1[ind1.1],levels=OTUs)))
Y1.2=prop.table(table(factor(XX1[ind1.2],levels=OTUs)))
Y1.3=prop.table(table(factor(XX1[ind1.3],levels=OTUs)))
Y2.1=prop.table(table(factor(XX2[ind2.1],levels=OTUs)))
Y2.2=prop.table(table(factor(XX2[ind2.2],levels=OTUs)))
Y2.3=prop.table(table(factor(XX2[ind2.3],levels=OTUs)))

YY=rbind(Y1.1,Y1.2,Y1.3,Y2.1,Y2.2,Y2.3)

BC=c()
for (i in 1:3){
  for (j in 4:6){
    BC=c(BC,vegan::vegdist(YY[c(i,j),], method=distancia))
  }
}
median(BC)
}
```

For reproducibility, we fix a random seed

```
#initial_seed=as.integer(Sys.time())
#print(initial_seed)
## [1] 1725986963
#initial_seed%%10000
## [1] 6963

set.seed(6963)
```

Let us perform the desired contrast for T0 vs T1 and the BC-distance

```
Sim.T0T1.T0.BC=replicate(1000,Simulation(c(1,2),"bray"))
Sim.T0.BC=replicate(1000,Simulation(c(1),"bray"))

saveRDS(Sim.T0T1.T0.BC, file="Sim.T0T1.T0.BC.RData")
saveRDS(Sim.T0.BC, file="Sim.T0.BC.RData")
```

The median BC-distances for the random T0+T1 samples go from 0.3877 to 0.3895, while the median BC-distances for the T0 samples go from 0.4057 to 0.407. So, **all** median BC-distances for T0 are larger than **any** median distance for T0+T1. Moreover, recall that the median BC-distance between the (reescaled) CAU\_T0 and CYM\_T0 samples was 0.4124, larger than the largest median BC-distance for the random T0+T1 samples.

For robustness, let us perform the contrast with T1 as base samples:

```
Sim.T0T1.T1.BC=replicate(1000,Simulation(c(2,1),"bray"))
Sim.T1.BC=replicate(1000,Simulation(c(2),"bray"))

saveRDS(Sim.T0T1.T1.BC, file="Sim.T0T1.T1.BC.RData")
saveRDS(Sim.T1.BC, file="Sim.T1.BC.RData")
```

In this case, the median BC-distances for the T0+T1 samples go from 0.3874 to 0.3894, while the median BC-distances for the T1 samples go from 0.3792 to 0.3807. The median BC-distance between the (reescaled) CAU\_T1 and CYM\_T1 samples was 0.4097.

Now T1 vs T2C, taking T1 as base samples (for the sake of completeness, we repeat the simulation for T1).

```
Sim.T1T2C.T1.BC=replicate(1000,Simulation(c(2,3),"bray"))
Sim.T1.BC.2=replicate(1000,Simulation(c(2),"bray"))

saveRDS(Sim.T1T2C.T1.BC, file="Sim.T1T2C.T1.BC.RData")
saveRDS(Sim.T1.BC.2, file="Sim.T1.BC.2.RData")
```

The median BC-distances for the T1+T2C samples go from 0.3971 to 0.3994, while the median BC-distances for the T1 samples go from 0.3792 to 0.3806. The median BC-distance between the (reescaled) CAU\_T1 and CYM\_T1 samples was 0.4097.

And T2C vs T2HW, with T2C as base samples.

```
Sim.T2CT2HW.T2C.BC=replicate(1000,Simulation(c(3,4),"bray"))
Sim.T2C.BC=replicate(1000,Simulation(c(3),"bray"))

saveRDS(Sim.T2CT2HW.T2C.BC,file="Sim.T2CT2HW.T2C.BC.RData")
saveRDS(Sim.T2C.BC, file="Sim.T2C.BC.RData")
```

The median BC-distances for the T2C+T2HW samples go from 0.385 to 0.3869, while the median BC-distances for the T2C samples go from 0.4274 to 0.4288. The median BC-distance between the (reescaled) CAU\_T2C and CYM\_T2C samples was 0.4493

Finally, the global samples:

```
Sim.Global.BC=replicate(1000,Simulation(c(1,2,3,4),"bray"))
Sim.T0.BC=replicate(1000,Simulation(c(1),"bray"))

saveRDS(Sim.Global.BC, file="Sim.Global.BC.RData")
saveRDS(Sim.T0.BC, file="Sim.T0.BC.RData")
```

The median BC-distances bewteen CAU and CYM for the Global samples go from 0.3805 to 0.3828, while the median BC-distances for the T0 samples go from 0.4057 to 0.407. The median BC-distance between the (reescaled) CAU\_T0 and CYM\_T0 samples was 0.4124

## 5 Differential abundance analysis

OTU differential abundance analyses were performed using **compositional data analysis** (**CoDA**) techniques.

For each pair of sample types considered, the analysis proceeded as follows:

1. We load *TablaOTUs.xlsx* and retain only the relevant “global” samples (all samples, only CAU samples, or only CYM samples, depending on the analysis).
2. To speed up computations, we remove from the dataset those OTUs with two or fewer reads across the selected samples, as they are unlikely to be relevant to the analysis.
3. Since CoDA methods typically rely on log-transformed proportions, zero counts must be replaced (imputed) with small positive values. To enable Bayesian-multiplicative zero imputation, we remove OTUs that appear in only one of the selected samples.
4. We then perform Bayesian-multiplicative zero imputation on the filtered count matrix using the `cmultRepl` function (`method = "GBM"`) from the `zCompositions` R package. This method replaces zeros with small positive values while preserving the compositional structure of the data.
5. We retain only the samples corresponding to the time points to be compared.
6. We assess whether, and how well, the two sample types are separated by hierarchical clustering.
7. We evaluate how well the two sample types separate when only random OTU subsets of fixed size are considered, for different subset sizes. This provides a reference against which we assess the significance of the separation observed for specific OTU groups.

To evaluate how a group of taxa separates the two sample groups, CAU_x_ and CYM_x_, we use the index  
\[
\frac{\text{distance between CAU${}\_x$ and CYM${}\_x$}}{\sqrt{(\text{distance within CAU${}\_x$})^2 + (\text{distance within CYM${}\_x$})^2}}
\]
where distances are calculated as the heights in the hierarchical tree obtained by clustering the samples using the selected taxa.

In each simulation, we fix the random seed as the last four digits of `Sys.time()` at the moment of execution.

8. We search for OTUs with differential abundance between the two sample types using several complementary approaches: significance according to ALDeX (adjusted p-values from the Kruskal–Wallis test), relevant log-ratios, bacterial signatures identified with `coda_glmnet`, and significance according to `simper`.
9. When different methods identify OTUs that consistently separate the two sample types, we record them in a table called `Significant`, which is finally exported as a `.csv` file. Its name is `"OTU_Significativos_"` followed by the pair of sample types.

### 5.1 CAU vs CYM

```
tax_otu=data.frame(read_excel("TablaOTUs.xlsx"))
Samples=tax_otu$ID 
n.OTUs.original=dim(tax_otu)[2]-2
taxa=colnames(tax_otu[,3:dim(tax_otu)[2]])
DF.0=tax_otu[,-c(1,2)]
taxa.original=taxa
OTUs=paste("OTU",1:n.OTUs.original,sep="")
colnames(DF.0)=OTUs
DF.0.total=DF.0
colnames(DF.0.total)=OTUs
rownames(DF.0.total)=Samples
rm(tax_otu)

Type=as.factor(rep(c("CAU","CYM"),each=12))
colors=c("green","blue")[Type]
#' Green: CAU  
#' Blue: CYM
```

The OTUs that appear with 2 or fewer hits in the total sample represent 15.8% of the total.

```
Miseria=as.vector(which(colSums(DF.0)<=2))
DF.0=DF.0[,-Miseria]
taxa=taxa[-Miseria]
OTUs=OTUs[-Miseria]
```

```
# DF.0.hits: for each OTU and each sample, 1 if the OTU is present in the sample and 0 otherwise
hit=function(x){min(c(x,1))}
DF.0.hits=as.matrix(DF.0)
DF.0.hits[]=vapply(DF.0.hits, hit, numeric(1))
```

OTUs that appear in only one sample but represent more than 0.05% of that sample:

```
Unics=data.frame(
Sample=rep(NA,length(which(colSums(DF.0.hits)==1))), OTUs=names(colSums(DF.0)[which(colSums(DF.0.hits)==1)]), Reads=colSums(DF.0)[which(colSums(DF.0.hits)==1)],
            Proportions=rep(0,length(which(colSums(DF.0.hits)==1)))  
)
for (i in 1:length(Unics$OTUs)){
ii=which(DF.0.hits[,Unics$OTUs[i]]==1)  
Unics$Proportions[i]=DF.0[ii,Unics$OTUs[i]]/sum(DF.0[ii,])
Unics$Sample[i]=Samples[ii]}
Unics.Freqs=Unics[Unics$Proportions>=0.05/100,]
row.names(Unics.Freqs)=NULL


Unics.Freqs[rev(order(Unics.Freqs$Proportions)),] %>%
  kbl() %>%
  kable_styling()
```

| Sample | OTUs | Reads | Proportions |
| --- | --- | --- | --- |
| CAU\_T0\_2 | OTU966 | 55 | 0.0012183 |

We remove OTUs that appear in only one sample and then perform Bayesian-multiplicative zero imputation.

```
DF.0=DF.0[,-which(colSums(DF.0.hits)==1)]
taxa=taxa[-which(colSums(DF.0.hits)==1)]
OTUs=OTUs[-which(colSums(DF.0.hits)==1)]
row.names(DF.0)=Samples
DF=zCompositions::cmultRepl(DF.0,method="GBM",output="p-counts",suppress.print=TRUE,z.warning=0.99)
row.names(DF)=Samples

rm(DF.0.hits)
rm(Miseria)
```

From 3088 initial taxa, we have reduced to 2542.

#### 5.1.1 How different are the samples?

```
HC=ClusterHC(DF,Grups=Type,barplot=FALSE,colores=colors)
```

```
separacio.global=Sep(HC)
```

The two sample types are perfectly separated from the start. The separation between groups is 2.47.

We observe below that the two sample types are so different that almost any subset of OTUs of reasonable size classifies them correctly. This will serve as a reference to compare how much improvement we gain with the “good” sets identified later.

```
> initial_seed=as.integer(Sys.time())
> print (initial_seed)
# [1] 1711395644
initial_seed %% 10000
# [1] 5644
```

```
set.seed(5644)
Experimento=function(i)
{
  ii=sample(dim(DF)[2],i,rep=FALSE)
  DFtemp=DF[,ii]
  CC=ClusterHC(DFtemp,dendrograma=FALSE,barplot=FALSE, Grups=Type,colores=colors)
  c(Encerts(CC$tabla,table(Type)),Sep(CC))
}
n=250
F.SS=c()
for (i in 10:round((dim(DF)[2])/2))
  {
  print(i)
  EE=replicate(n,Experimento(i))
  EE.2=EE[2,which(EE[1,]==0)]
XX=replicate(1000,mean(sample(EE.2,n,replace=TRUE)))
  F.SS=rbind(F.SS, c(i,
                     length(EE.2)/250,
                      mean(EE.2),
                      quantile(XX,0.025),
                      quantile(XX,0.975)
  ))
  }
colnames(F.SS)=c("n","Proportion","Avg. sep.","Q_0.025","Q_0.975")
saveRDS(F.SS, file="Fraccio.Separadors.CAUvsCYM.RData")
```

```
F.SS=readRDS("Fraccio.Separadors.CAUvsCYM.RData")
```

The first plot shows, for each \(n\) up to half the total number of OTUs, the proportion of samples of size \(n\) that perfectly classify CAU and CYM (black curve), along with the 95% Clopper–Pearson confidence interval for that proportion (red curves). We have used *smoothing splines* to smooth the curves.

```
F.SS_prop.Q_0.025=epitools::binom.exact(F.SS[,2]*250,250)$lower
F.SS_prop.Q_0.975=epitools::binom.exact(F.SS[,2]*250,250)$upper
fit = smooth.spline(F.SS[,1],F.SS[,2],cv=TRUE)
fit.low = smooth.spline(F.SS[,1],F.SS_prop.Q_0.025,cv=TRUE)
fit.up = smooth.spline(F.SS[,1],F.SS_prop.Q_0.975,cv=TRUE)


plot(F.SS[,1:2],pch=20,cex=0.25,col="darkgrey",xlab="n",ylab="Proportion",main="Proportion of perfect classifiers",xlim=c(0,1250),xaxp=c(0,1250,25),yaxp=c(0,1,10))
lines(fit,col="black",lwd=1)
lines(fit.low,col="red",lwd=1)
lines(fit.up,col="red",lwd=1)
```

The second plot shows, for each \(n\) up to half the total number of OTUs, the estimated mean separation obtained with a sample of size \(n\) that perfectly classifies CAU and CYM (black curve), along with the 95% confidence interval of this mean separation obtained via bootstrap (red curve). This plot will serve as a reference later.

```
fit = smooth.spline(F.SS[,1],F.SS[,3],cv=TRUE)
fit.low = smooth.spline(F.SS[,1],F.SS[,4],cv=TRUE)
fit.up = smooth.spline(F.SS[,1],F.SS[,5],cv=TRUE)

plot(F.SS[,c(1,3)],pch=20,cex=0.25,col="darkgrey",xlab="n",ylab="Separation",main="Average separation for perfect classifiers",xlim=c(0,1250),xaxp=c(0,1250,25),yaxp=c(0,2.5,10))
lines(fit,col="black",lwd=1)
lines(fit.low,col="red",lwd=1)
lines(fit.up,col="red",lwd=1)
abline(h=separacio.global,col="green")
```

`

#### 5.1.2 OTUs significant according to ALDeX

We always use the `aldexCesc.clr` version of the `aldex.clr` function (from the script **funcionsCODAMETACIRCLE.R**) to generate the simulated samples. These simulated instances are then used by `aldex.kw` to estimate adjusted Kruskal-Wallis p-values in a way that reflects the uncertainty introduced by the compositional structure and the zero replacement step.

```
> initial_seed=as.integer(Sys.time())
> print (initial_seed)
# [1] 1711411362
initial_seed %% 10000
# [1] 1362
```

```
set.seed(1362)
repl.kw.Cescv2.CaCy=aldexCesc.clr(t(DF), conds=Type, mc.samples=1000, verbose=FALSE)
aldex.kw.Cescv2.CaCy=aldex.kw(repl.kw.Cescv2.CaCy, verbose=FALSE)
#'
saveRDS(aldex.kw.Cescv2.CaCy, file="aldex_kw_Cescv2_CaCy.RData")
```

```
p.valores_kw=readRDS("aldex_kw_Cescv2_CaCy.RData")
```

**Adjusted Kruskal–Wallis p-value < 0.05**

```
sep.kw.005=QQ.HC.noadjust(p.valores_kw,DF,Type,q=0.05,1)
```

```
kw.005=which(p.valores_kw[,2]<0.05)
```

There are 804 taxa with adjusted Kruskal–Wallis p-value < 0.05.  
The separation between groups is 2.48.

**Adjusted Kruskal–Wallis p-value < 0.01**

```
sep.kw.001=QQ.HC.noadjust(p.valores_kw,DF,Type,q=0.01,1)
```

```
kw.001=which(p.valores_kw[,2]<0.01)
```

There are 605 taxa with adjusted Kruskal–Wallis p-value < 0.01.  
The separation between groups is 2.35.

**Adjusted Kruskal–Wallis p-value < 0.001**

```
sep.kw.0001=QQ.HC.noadjust(p.valores_kw,DF,Type,q=0.001,1)
```

```
kw.0001=which(p.valores_kw[,2]<0.001)
```

There are 408 taxa with adjusted Kruskal–Wallis p-value < 0.001.  
The separation between groups is 2.89.

```
Indicador=function(x,k=n.OTUs.original){
  xx=rep(0,k)
  y=as.numeric(gsub("\\D+", "", OTUs[x]))
  xx[y]=1
  
  return(xx)
}
```

```
Significant=data.frame(
  OTUs=paste("OTU",1:n.OTUs.original,sep=""),
  taxa=taxa.original,
  Aldex.kw.0.05=Indicador(kw.005),
  Aldex.kw.0.01=Indicador(kw.001),
  Aldex.kw.0.001=Indicador(kw.0001)
  )
```

#### 5.1.3 Taxa with maximum relevant log-ratios

Here we apply the **log-ratio (LR)** approach implemented in `explore_logratios` from the `coda4microbiome` package. For each pair of OTUs, the method quantifies how strongly the log-ratio `log(p_i/p_j)` is associated with the binary classification of interest. Based on these associations, OTUs are ranked by their overall log-ratio relevance.

Starting from the LR matrix, we proceed as follows: for each `k`, we compute the mean of the absolute values of the entries in the submatrix formed by the first `k` rows and columns of the LR matrix:

\[
\text{assoc}\_k = \frac{1}{k^2}\sum\_{1 \le i,j \le k} \left|\, lrcor\_{i,j} \,\right|.
\]

We then identify the value of `k` for which \(\text{assoc}\_k\) reaches its maximum, which corresponds to the subset of the `k` most log-ratio–relevant OTUs showing the highest average absolute log-ratio correlation among them. This subset of `k` OTUs is retained for subsequent analyses.

Finally, we plot the \(\text{assoc}\_k\) values (see main text) and select the `k` at which the curve attains its maximum.

```
LRS=explore_logratios(DF,Type)
saveRDS(LRS, file="LRS_CAUCYM.RData")
```

```
LRS=readRDS("LRS_CAUCYM.RData")
```

```
assoc=rep(0,dim(DF)[2]-4)
for (m in 5:dim(DF)[2]){
    assoc[m-4]=sum(LRS$`association log-ratio with y`[1:m,1:m])/m^2
}
saveRDS(assoc, file="assoc_CAUCYM.RData")
```

```
assoc=readRDS("assoc_CAUCYM.RData")
plot(5:dim(DF)[2],assoc,type="b",xlab="m",ylab="Average goodness of the m most logratio-relevant taxa."
     ,pch=20,cex=0.5)
```

```
m=4+which.max(assoc)
impLR=sort(as.numeric(LRS$`order of importance`[1:m]))
DF.Imp=DF[,impLR]
HC=ClusterHC(DF.Imp,dendrograma=FALSE,barplot=FALSE, Grups=Type)
LR.max=c(length(impLR),Sep(HC))
```

The maximum is attained at the set of 103 most logratio-relevant taxa.  
They perfectly separate the two sample types. The separation between groups is 6.58.

```
Significant=cbind(Significant,
                     Log_ratio.max=Indicador(impLR))
```

#### 5.1.4 Taxa relevant for the bacterial signature (according to `coda_glmnet`)

We apply `coda_glmnet` to the zero-imputed count matrix. This method fits a penalized log-contrast model for binary classification using all pairwise log-ratios, and then rewrites the fitted model in terms of single log-proportions. OTUs with non-zero coefficients form the microbial signature for the comparison, while the sign of each coefficient indicates in which sample group the corresponding OTU is relatively more abundant.

```
coda.glmnet=coda_glmnet(DF,Type,showPlots = FALSE)
saveRDS(coda.glmnet, file="coda.glmnet.CAUCYM.RData")
```

```
coda.glmnet=readRDS("coda.glmnet.CAUCYM.RData")
coda.glmnet$`signature plot`
```

```
impGLMNET=coda.glmnet$taxa.num
DF.Imp=DF[,impGLMNET]
HC=ClusterHC(DF.Imp,dendrograma=TRUE,barplot=FALSE,Grups=Type,colores=colors)
```

```
SB.glmnet=c(length(coda.glmnet$taxa.num),Sep(HC))
```

There are 118 significant taxa.  
The separation between groups is 4.55.

```
Significant=cbind(Significant,
glmnet_sign.CAU=Indicador(coda.glmnet$taxa.num[coda.glmnet$`log-contrast coefficients`>0]),
glmnet_sign.CYM=Indicador(coda.glmnet$taxa.num[coda.glmnet$`log-contrast coefficients`<0]))
```

#### 5.1.5 Simper

We use `simper` from `vegan` on the original proportion matrix as a complementary, non-CoDA ranking of the taxa contributing most to between-group dissimilarity. We then evaluate whether the OTUs retained by this approach also produce a strong separation in the compositional hierarchical clustering.

```
DF.prop=DF.0.total/rowSums(DF.0.total)
SMP.prop=simper(DF.prop,group=Type)
```

```
indexos_SMP.rel.prop=SMP.prop$CAU_CYM$ord[which(SMP.prop$CAU_CYM$cusum<=0.7)]
OTUs.simper.rel.prop=sort(attr(SMP.prop$CAU_CYM$cusum[which(SMP.prop$CAU_CYM$cusum<=0.7)],"names"))

DF.Imp=DF[,OTUs.simper.rel.prop]
HC=ClusterHC(DF.Imp,dendrograma=TRUE,barplot=FALSE,Grups=Type,colores=colors)
```

```
simper.rel.props=c(length(indexos_SMP.rel.prop),Sep(HC))
```

There are 93 significant taxa. They perfectly separate the two sample types. The separation between groups is 2.88.

```
Indicador.Gl=function(x,k=n.OTUs.original){
  xx=rep(0,k)
 xx[x]=1
  return(xx)
}

Significant=cbind(Significant,
simper.props=Indicador.Gl(indexos_SMP.rel.prop))
```

```
Significant=cbind(Significant,
t(DF.prop))
```

#### 5.1.6 Summary

```
write.csv2(Significant,"OTU_Significativos_CAUvsCYM_def.csv",row.names=FALSE)
```

```
Resultados=rbind(
sep.kw.0001,sep.kw.005,sep.kw.001,
LR.max,SB.glmnet,
simper.rel.props
)
row.names(Resultados)=c("Aldex.0.001","Aldex.0.05","Aldex.0.01",
"LR","glmnet", "simper")
colnames(Resultados)=c("Size","Separation")
```

```
par(opar)
fit = smooth.spline(F.SS[,1],F.SS[,3],cv=TRUE)
fit.low = smooth.spline(F.SS[,1],F.SS[,4],cv=TRUE)
fit.up = smooth.spline(F.SS[,1],F.SS[,5],cv=TRUE)

plot(F.SS[,c(1,3)],pch=20,cex=0.25,col="darkgrey",xlab="n",ylab="Separation",main="Average separation for perfect classifiers",xaxp=c(0,1000,40),yaxp=c(0,8.5,17),ylim=c(0,8.5),xlim=c(0,1000))
lines(fit,col="black",lwd=1)
lines(fit.low,col="red",lwd=1)
lines(fit.up,col="red",lwd=1)
points(Resultados,col="blue",type="h")
points(Resultados,col="blue",pch=20)
text(Resultados[,1],Resultados[,2]+0.1,col="blue",labels=row.names(Resultados),cex=0.75)
abline(h=2.47,col="green")
text(20,2.6,col="green",labels="Global",cex=0.75)
```

Finally, we intersect the OTUs identified as significant according to LR and `coda_glmnet`.

```
Significant.capat=Significant[,c(1,2,6,7,8)]
Significant.capat=cbind(Significant.capat,Cuánto=rowSums(Significant.capat[,3:dim(Significant.capat)[2]]))
Significant.capat=Significant.capat[Significant.capat$Cuánto==2,]
```

There are 41 such taxa. They are

```
Significant.capat[,1:2]%>%
  kbl() %>%
  kable_styling()
```

|  | OTUs | taxa |
| --- | --- | --- |
| OTU22 | OTU22 | d\_\_Bacteria.p\_\_Desulfobacterota.c\_\_Desulfobacteria.o\_\_Desulfobacterales.f\_\_Desulfosarcinaceae.g\_\_Desulfatitalea.s\_\_uncultured\_Desulfobacteraceae |
| OTU30 | OTU30 | d\_\_Bacteria.p\_\_Bacteroidota.c\_\_Ignavibacteria.o\_\_Ignavibacteriales.f\_\_PHOS.HE36.g\_\_PHOS.HE36.s\_\_uncultured\_bacterium |
| OTU32 | OTU32 | d\_\_Bacteria.p\_\_Desulfobacterota.c\_\_Desulfobacteria.o\_\_Desulfobacterales.f\_\_Desulfosarcinaceae.g**uncultured.** |
| OTU35 | OTU35 | d\_\_Bacteria.p\_\_Desulfobacterota.c\_\_Desulfobacteria.o\_\_Desulfobacterales.f\_\_Desulfosarcinaceae.g\_\_LCP.80.s\_\_uncultured\_delta |
| OTU38 | OTU38 | Unassigned.\_\_.**.**.**.**.\_\_ |
| OTU39 | OTU39 | d\_\_Bacteria.p\_\_Bacteroidota.c\_\_Bacteroidia.o\_\_Sphingobacteriales.f\_\_Lentimicrobiaceae.g\_\_Lentimicrobiaceae.s\_\_uncultured\_Bacteroidetes |
| OTU42 | OTU42 | d\_\_Bacteria.p\_\_Myxococcota.c\_\_Polyangia.o\_\_Polyangiales.f\_\_Sandaracinaceae.g**uncultured.** |
| OTU45 | OTU45 | d\_\_Bacteria.p\_\_Desulfobacterota.c\_\_Desulfobulbia.o\_\_Desulfobulbales.f\_\_Desulfocapsaceae.**.** |
| OTU46 | OTU46 | d\_\_Bacteria.p\_\_Bacteroidota.c\_\_Ignavibacteria.o\_\_Ignavibacteriales.f\_\_Ignavibacteriaceae.g**Ignavibacterium.** |
| OTU70 | OTU70 | d\_\_Bacteria.p\_\_Desulfobacterota.c\_\_Desulfobacteria.o**Desulfobacterales.**.**.** |
| OTU109 | OTU109 | d\_\_Bacteria.p\_\_Desulfobacterota.c\_\_Desulfobacteria.o\_\_Desulfobacterales.f\_\_Desulfosarcinaceae.g**LCP.80.** |
| OTU133 | OTU133 | d\_\_Bacteria.p\_\_Desulfobacterota.c\_\_Desulfobacteria.o\_\_Desulfatiglandales.f\_\_Desulfatiglandaceae.g**Desulfatiglans.** |
| OTU141 | OTU141 | d\_\_Bacteria.p\_\_Bdellovibrionota.c\_\_Oligoflexia.o\_\_0319.6G20.f\_\_0319.6G20.g**0319.6G20.** |
| OTU213 | OTU213 | d\_\_Bacteria.p\_\_Desulfobacterota.c\_\_Desulfobacteria.o\_\_Desulfobacterales.f\_\_Desulfosarcinaceae.g**Desulfatitalea.** |
| OTU218 | OTU218 | d\_\_Bacteria.p\_\_Spirochaetota.c\_\_Leptospirae.o\_\_Leptospirales.f\_\_Leptospiraceae.g\_\_RBG.16.49.21.s\_\_uncultured\_organism |
| OTU223 | OTU223 | d\_\_Bacteria.p\_\_Bacteroidota.c\_\_Bacteroidia.o\_\_Sphingobacteriales.f\_\_ST.12K33.g\_\_ST.12K33.s\_\_uncultured\_Cytophagales |
| OTU229 | OTU229 | d\_\_Bacteria.p\_\_Spirochaetota.c\_\_Leptospirae.o\_\_Leptospirales.f\_\_Leptospiraceae.g\_\_RBG.16.49.21.s\_\_uncultured\_bacterium |
| OTU230 | OTU230 | d\_\_Bacteria.p\_\_Bacteroidota.c\_\_Bacteroidia.o\_\_Cytophagales.f\_\_Cyclobacteriaceae.g\_\_uncultured.s\_\_uncultured\_Bacteroidetes |
| OTU253 | OTU253 | d\_\_Bacteria.p\_\_Desulfobacterota.c\_\_Desulfobacteria.o\_\_Desulfatiglandales.f\_\_Desulfatiglandaceae.g\_\_Desulfatiglans.s\_\_uncultured\_delta |
| OTU255 | OTU255 | d\_\_Bacteria.p\_\_Spirochaetota.c\_\_V2072.189E03.o\_\_V2072.189E03.f\_\_V2072.189E03.g\_\_V2072.189E03.s\_\_uncultured\_organism |
| OTU256 | OTU256 | d\_\_Bacteria.p\_\_Fermentibacterota.c\_\_Fermentibacteria.o\_\_Fermentibacterales.f\_\_Fermentibacteraceae.g\_\_Fermentibacteraceae.s\_\_uncultured\_bacterium |
| OTU269 | OTU269 | d\_\_Bacteria.p\_\_Proteobacteria.c\_\_Gammaproteobacteria.o\_\_Chromatiales.f\_\_Chromatiaceae.g**Candidatus\_Thiobios.** |
| OTU451 | OTU451 | d\_\_Bacteria.p\_\_Bacteroidota.c\_\_Bacteroidia.o\_\_Flavobacteriales.f\_\_Flavobacteriaceae.g**Actibacter.** |
| OTU498 | OTU498 | d\_\_Bacteria.p\_\_Desulfobacterota.c\_\_Desulfobacteria.o\_\_Desulfatiglandales.f\_\_Desulfatiglandaceae.g\_\_Desulfatiglans.s\_\_uncultured\_organism |
| OTU527 | OTU527 | d\_\_Bacteria.p\_\_Desulfobacterota.c\_\_Desulfobacteria.o\_\_Desulfobacterales.f\_\_Desulfobacteraceae.g\_\_Desulfotignum.s\_\_uncultured\_bacterium |
| OTU761 | OTU761 | d\_\_Bacteria.p\_\_Actinobacteriota.c\_\_Acidimicrobiia.o\_\_Microtrichales.f\_\_Microtrichaceae.g**Sva0996\_marine\_group.** |
| OTU811 | OTU811 | d\_\_Bacteria.p\_\_Desulfobacterota.c\_\_Desulfobacteria.o\_\_Desulfobacterales.f\_\_Desulfosarcinaceae.g\_\_Desulfosarcina.s\_\_uncultured\_bacterium |
| OTU881 | OTU881 | d\_\_Bacteria.p\_\_Proteobacteria.c\_\_Gammaproteobacteria.o\_\_Thiotrichales.f\_\_Thiotrichaceae.g\_\_uncultured.s\_\_uncultured\_gamma |
| OTU890 | OTU890 | d\_\_Bacteria.p\_\_Bacteroidota.c\_\_Bacteroidia.o\_\_Flavobacteriales.f\_\_Flavobacteriaceae.g\_\_Robiginitalea.s\_\_uncultured\_Bacteroidetes |
| OTU1107 | OTU1107 | d\_\_Bacteria.p\_\_Desulfobacterota.c\_\_Desulfobacteria.o\_\_Desulfobacterales.f\_\_Desulfosarcinaceae.g\_\_Sva0081\_sediment\_group.s\_\_metagenome |
| OTU1329 | OTU1329 | d\_\_Bacteria.p\_\_Desulfobacterota.c\_\_Desulfobacteria.o\_\_Desulfobacterales.f\_\_Desulfococcaceae.g**Desulfonema.** |
| OTU1390 | OTU1390 | d\_\_Bacteria.p\_\_Gemmatimonadota.c\_\_PAUC43f\_marine\_benthic\_group.o\_\_PAUC43f\_marine\_benthic\_group.f\_\_PAUC43f\_marine\_benthic\_group.g\_\_PAUC43f\_marine\_benthic\_group.s\_\_saltmarsh\_clone |
| OTU1419 | OTU1419 | d\_\_Bacteria.p\_\_NB1.j.c\_\_NB1.j.o\_\_NB1.j.f\_\_NB1.j.g\_\_NB1.j.s\_\_uncultured\_bacterium |
| OTU1443 | OTU1443 | d\_\_Bacteria.p\_\_Proteobacteria.c\_\_Gammaproteobacteria.o\_\_B2M28.f\_\_B2M28.g\_\_B2M28.s\_\_uncultured\_proteobacterium |
| OTU1461 | OTU1461 | d\_\_Bacteria.p\_\_Bacteroidota.c\_\_Bacteroidia.o\_\_Flavobacteriales.f\_\_Flavobacteriaceae.g\_\_Robiginitalea.s\_\_Robiginitalea\_myxolifaciens |
| OTU1532 | OTU1532 | d\_\_Bacteria.p\_\_Proteobacteria.c\_\_Gammaproteobacteria.o\_\_Chromatiales.f\_\_Chromatiaceae.g\_\_Candidatus\_Thiobios.s\_\_uncultured\_organism |
| OTU1594 | OTU1594 | d\_\_Bacteria.p\_\_Bacteroidota.c\_\_Ignavibacteria.o\_\_Ignavibacteriales.f\_\_PHOS.HE36.g**PHOS.HE36.** |
| OTU1701 | OTU1701 | d\_\_Bacteria.p\_\_Proteobacteria.c\_\_Alphaproteobacteria.o\_\_Rhizobiales.f\_\_Methyloligellaceae.g**Methyloceanibacter.** |
| OTU1839 | OTU1839 | d\_\_Bacteria.p\_\_Proteobacteria.c\_\_Gammaproteobacteria.o\_\_Ectothiorhodospirales.f\_\_Thioalkalispiraceae.g\_\_Thioalkalispira.Sulfurivermis.s\_\_uncultured\_Thioalkalispiraceae |
| OTU2338 | OTU2338 | d\_\_Bacteria.p\_\_Cyanobacteria.c\_\_Cyanobacteriia.o\_\_Cyanobacteriales.f\_\_Xenococcaceae.g**Xenococcus\_PCC.7305.** |
| OTU2371 | OTU2371 | d\_\_Bacteria.p\_\_Firmicutes.c\_\_Clostridia.o\_\_Lachnospirales.f\_\_Lachnospiraceae.g**Roseburia.** |

```
DF.Imp=DF[,Significant.capat[,1]]
HC=ClusterHC(DF.Imp,dendrograma=TRUE,barplot=FALSE,Grups=Type,colores=colors)
```

The separation between groups is 7.86.

We update the plot with the separations:

```
intersección=c(dim(Significant.capat)[1],Sep(HC))
Resultados=rbind(Resultados,intersección)
row.names(Resultados)=c("Aldex.0.001","Aldex.0.05","Aldex.0.01",
"LR","glmnet", "simper","intersection")
colnames(Resultados)=c("Size","Separation")
```

```
par(opar)
fit = smooth.spline(F.SS[,1],F.SS[,3],cv=TRUE)
fit.low = smooth.spline(F.SS[,1],F.SS[,4],cv=TRUE)
fit.up = smooth.spline(F.SS[,1],F.SS[,5],cv=TRUE)

plot(F.SS[,c(1,3)],pch=20,cex=0.25,col="darkgrey",xlab="n",ylab="Separation",main="Average separation for perfect classifiers",xaxp=c(0,1000,40),yaxp=c(0,8.5,17),ylim=c(0,8.5),xlim=c(0,1000))
lines(fit,col="black",lwd=1)
lines(fit.low,col="red",lwd=1)
lines(fit.up,col="red",lwd=1)
points(Resultados,col="blue",type="h")
points(Resultados,col="blue",pch=20)
text(Resultados[,1],Resultados[,2]+0.1,col="blue",labels=row.names(Resultados),cex=0.75)
abline(h=2.47,col="green")
text(20,2.6,col="green",labels="Global",cex=0.75)
```

```
Resultados %>%
  kbl() %>%
  kable_styling()
```

|  | Size | Separation |
| --- | --- | --- |
| Aldex.0.001 | 408 | 2.889614 |
| Aldex.0.05 | 804 | 2.481799 |
| Aldex.0.01 | 605 | 2.354071 |
| LR | 103 | 6.579296 |
| glmnet | 118 | 4.550477 |
| simper | 93 | 2.881239 |
| intersection | 41 | 7.863271 |

### 5.2 CAU T0 vs CYM T0

```
tax_otu=data.frame(read_excel("TablaOTUs.xlsx"))
Samples=tax_otu$ID 
taxa=colnames(tax_otu[,3:dim(tax_otu)[2]])
OTUs=paste("OTU",1:(dim(tax_otu)[2]-2),sep="")


DF.0=tax_otu[c(1:3,13:15),-c(1,2)]
row.names(DF.0)=Samples[c(1:3,13:15)]
colnames(DF.0)=OTUs

taxa.original=taxa
DF.0.original=DF.0
n.OTUs.original=dim(DF.0)[2]

Miseria=as.vector(which(colSums(DF.0)<=2))
DF.0=DF.0[,-Miseria]
taxa=taxa[-Miseria]
OTUs=OTUs[-Miseria]

hit=function(x){min(c(x,1))}
DF.0.hits=as.matrix(DF.0)
DF.0.hits[]=vapply(DF.0.hits, hit, numeric(1))

Unics=data.frame(
Sample=rep(NA,length(which(colSums(DF.0.hits)==1))), OTUs=names(colSums(DF.0)[which(colSums(DF.0.hits)==1)]), Reads=colSums(DF.0)[which(colSums(DF.0.hits)==1)],
            Proportions=rep(0,length(which(colSums(DF.0.hits)==1)))  
)
for (i in 1:length(Unics$OTUs)){
ii=which(DF.0.hits[,Unics$OTUs[i]]==1)  
Unics$Proportions[i]=DF.0[ii,Unics$OTUs[i]]/sum(DF.0[ii,])
Unics$Sample[i]=Samples[ii]}
Unics.Freqs=Unics[Unics$Proportions>=0.05/100,]
row.names(Unics.Freqs)=NULL


DF.0=DF.0[,-which(colSums(DF.0.hits)==1)]
taxa=taxa[-which(colSums(DF.0.hits)==1)]
OTUs=OTUs[-which(colSums(DF.0.hits)==1)]

row.names(DF.0)=Samples[c(1:3,13:15)]

DF=zCompositions::cmultRepl(DF.0,method="GBM",output="p-counts",suppress.print=TRUE,z.warning=0.99)
row.names(DF)=Samples[c(1:3,13:15)]


Type=as.factor(c(rep("CAU_T0", 3),rep("CYM_T0", 3)))
colors=c("green","blue")[Type]
```

After removing OTUs with 2 or fewer reads across the T0 samples and those appearing in only one of these samples, 1472 taxa remain.

The OTUs removed at this step that represent more than 0.05% of the sample in which they appear are:

```
Unics.Freqs[rev(order(Unics.Freqs$Proportions)),] %>%
  kbl() %>%
  kable_styling()
```

| Sample | OTUs | Reads | Proportions |
| --- | --- | --- | --- |
| CAU\_T0\_2 | OTU966 | 55 | 0.0012208 |

```
rm(tax_otu)
rm(DF.0.hits)
rm(Miseria)
rm(Unics.Freqs)
```

#### 5.2.1 How different are the samples?

```
HC=ClusterHC(DF,Grups=Type,barplot=FALSE,colores=colors)
```

```
separacio.global=Sep(HC)
```

The two sample types are perfectly separated from the start. The separation between groups is 2.43.

We examine the proportion of OTU subsets of reasonable size that already classify them correctly.

```
> initial_seed=as.integer(Sys.time())
> print (initial_seed)
# [1] 1717567904
initial_seed %% 10000
# [1] 7904
```

```
set.seed(7904)
Experimento=function(i)
{
  ii=sample(dim(DF)[2],i,rep=FALSE)
  DFtemp=DF[,ii]
  CC=ClusterHC(DFtemp,dendrograma=FALSE,barplot=FALSE, Grups=Type,colores=colors)
  c(Encerts(CC$tabla,table(Type)),Sep(CC))
}
n=250
F.SS=c()
for (i in 10:750)
  {
  print(i)
  EE=replicate(n,Experimento(i))
  EE.2=EE[2,which(EE[1,]==0)]
XX=replicate(1000,mean(sample(EE.2,n,replace=TRUE)))
  F.SS=rbind(F.SS, c(i,
                     length(EE.2)/250,
                      mean(EE.2),
                      quantile(XX,0.025),
                      quantile(XX,0.975)
  ))
  }
colnames(F.SS)=c("n","proporción","sep. media","Q_0.025","Q_0.975")
saveRDS(F.SS, file="Fraccio.Separadors.T0.RData")
```

```
F.SS=readRDS("Fraccio.Separadors.T0.RData")
```

The first plot shows, for each \(n\) up to half the total number of OTUs, the proportion of samples of size \(n\) that perfectly classify CAU\_T0 and CYM\_T0 (black curve), along with the 95% Clopper–Pearson confidence interval for that proportion (red curves). We used *smoothing splines* to smooth the curves.

```
F.SS_prop.Q_0.025=epitools::binom.exact(F.SS[,2]*250,250)$lower
F.SS_prop.Q_0.975=epitools::binom.exact(F.SS[,2]*250,250)$upper
fit = smooth.spline(F.SS[,1],F.SS[,2],cv=TRUE)
fit.low = smooth.spline(F.SS[,1],F.SS_prop.Q_0.025,cv=TRUE)
fit.up = smooth.spline(F.SS[,1],F.SS_prop.Q_0.975,cv=TRUE)


plot(F.SS[,1:2],pch=20,cex=0.25,col="darkgrey",xlab="n",ylab="Proportion",main="Proportion of perfect classifiers",xlim=c(0,750),xaxp=c(0,750,15),yaxp=c(0,1,10))
lines(fit,col="black",lwd=1)
lines(fit.low,col="red",lwd=1)
lines(fit.up,col="red",lwd=1)
```

The second plot shows, for each \(n\) up to half the total number of OTUs, the estimated mean separation obtained with a sample of size \(n\) that perfectly classifies CAU\_T0 and CYM\_T0 (black curve), along with the 95% confidence interval of this mean separation obtained via bootstrap (red curve).

```
fit = smooth.spline(F.SS[,1],F.SS[,3],cv=TRUE)
fit.low = smooth.spline(F.SS[,1],F.SS[,4],cv=TRUE)
fit.up = smooth.spline(F.SS[,1],F.SS[,5],cv=TRUE)

plot(F.SS[,c(1,3)],pch=20,cex=0.25,col="darkgrey",xlab="n",ylab="Separation",main="Average separation for perfect classifiers",xlim=c(0,750),xaxp=c(0,750,15),yaxp=c(0,2.5,10))
lines(fit,col="black",lwd=1)
lines(fit.low,col="red",lwd=1)
lines(fit.up,col="red",lwd=1)
abline(h=separacio.global,col="green")
```

`

#### 5.2.2 OTUs significant according to ALDeX

```
> initial_seed=as.integer(Sys.time())
> print (initial_seed)
# [1] 1717574148
initial_seed %% 10000
# [1] 4148
```

```
set.seed(4148)
repl.kw.Cescv2.T0=aldexCesc.clr(t(DF), conds=Type, mc.samples=1000, verbose=FALSE)
aldex.kw.Cescv2.T0=aldex.kw(repl.kw.Cescv2.T0, verbose=FALSE)
#'
saveRDS(aldex.kw.Cescv2.T0, file="aldex_kw_Cescv2.T0.RData")
```

**Taxa with statistically significant differences:**

```
p.valores_kw=readRDS("aldex_kw_Cescv2.T0.RData")
```

```
length(p.valores_kw[p.valores_kw[,2]<0.05,2]) #K-W adjusted p-value < 0.05
```

```
## [1] 0
```

```
length(p.valores_kw[p.valores_kw[,1]<0.05,2]) #p-value < 0.05
```

```
## [1] 121
```

```
length(p.valores_kw[p.valores_kw[,1]<0.01,4]) #p-value < 0.01
```

```
## [1] 0
```

There is no OTU with an adjusted Kruskal–Wallis test p-value < 0.05. There are 121 with an unadjusted p-value < 0.05 (and none < 0.01). They classify the samples correctly:

```
sep.kw.005=QQ.HC.noadjust(p.valores_kw,DF,Type,q=0.05,1,BP=FALSE)
```

```
kw.005=which(p.valores_kw[,2]<0.001)
```

The separation between groups is 3.43.

```
Significant=data.frame(
  OTUs=paste("OTU",1:n.OTUs.original,sep=""),
  taxa=taxa.original,
  Aldex.kw.0.05=Indicador(kw.005)
)
```

#### 5.2.3 Taxa with maximum relevant log-ratios

We apply the LR approach exactly as described in the first comparison: OTUs are ranked by their log-ratio relevance, and we retain the subset maximizing the average absolute within-subset log-ratio association.

```
LRS=explore_logratios(DF,Type)
saveRDS(LRS, file="LRS_T0.RData")
```

```
LRS=readRDS("LRS_T0.RData")
```

```
assoc=rep(0,dim(DF)[2]-4)
for (m in 5:dim(DF)[2]){
    assoc[m-4]=sum(LRS$`association log-ratio with y`[1:m,1:m])/m^2
}
saveRDS(assoc, file="assoc_T0.RData")
```

```
assoc=readRDS("assoc_T0.RData")
plot(5:200,assoc[1:196],type="b",xlab="m",ylab="Bondad mediana de los m más relevantes"
     ,pch=20,cex=0.5)
```

```
m=4+which.max(assoc)
impLR=sort(as.numeric(LRS$`order of importance`[1:m]))
DF.Imp=DF[,impLR]
HC=ClusterHC(DF.Imp,dendrograma=FALSE,barplot=FALSE, Grups=Type)
LR.max=c(length(impLR),Sep(HC))
```

The maximum is attained at the set of 549 most logratio-relevant taxa.  
They perfectly separate the two sample types. The separation between groups is 2.83.

**Note** In the plot we can observe a very pronounced local maximum at 46. Let us examine it:

```
m0=4+which.max(assoc[1:100])
impLR.2=sort(as.numeric(LRS$`order of importance`[1:m0]))
DF.Imp.0=DF[,impLR.2]
HC=ClusterHC(DF.Imp.0,dendrograma=TRUE,barplot=FALSE, Grups=Type)
```

```
LR.max.2=c(length(impLR.2),Sep(HC))
```

They also perfectly separate the two sample types. The separation between groups is 4.66. Fewer taxa, but better separation.

```
Significant=cbind(Significant,
                     Log_ratio.max=Indicador(impLR),
                     Log_ratio.max2=Indicador(impLR.2))
```

#### 5.2.4 Taxa relevant for the bacterial signature (according to `coda_glmnet`)

As described above, `coda_glmnet` identifies a microbial signature by fitting a penalized log-contrast model and retaining OTUs with non-zero coefficients.

```
coda.glmnet=coda_glmnet(DF,Type,showPlots = FALSE)
saveRDS(coda.glmnet, file="coda.glmnet.T0.RData")
```

```
coda.glmnet=readRDS("coda.glmnet.T0.RData")
coda.glmnet$`signature plot`
```

```
impGLMNET=coda.glmnet$taxa.num
DF.Imp=DF[,impGLMNET]
HC=ClusterHC(DF.Imp,dendrograma=TRUE,barplot=FALSE,Grups=Type,colores=colors)
```

```
glmnet.no0=c(length(coda.glmnet$taxa.num),Sep(HC))
```

There are 350 significant taxa.  
The separation between groups is 2.78.

```
Significant=cbind(Significant,
glmnet_sign.no0.CAU=Indicador(coda.glmnet$taxa.num[coda.glmnet$`log-contrast coefficients`>0]),
glmnet_sign.no0.CYM=Indicador(coda.glmnet$taxa.num[coda.glmnet$`log-contrast coefficients`<0]))
```

Because `coda_glmnet` may be unstable with only 3 vs. 3 samples, we also explore an augmented analysis based on three ALDEx-derived random replicates per sample.

```
> initial_seed=as.integer(Sys.time())
> print (initial_seed)
# [1] 1717687628
initial_seed %% 10000
# [1] 7628
```

```
M=3
set.seed(7628)
repls=aldexCesc.clr(t(DF), conds=Type, mc.samples=M)@analysisData
```

```
## [1] "operating in serial mode"
```

```
repls=t(cbind(
  repls$CAU_T0_1,repls$CAU_T0_2,repls$CAU_T0_3,
    repls$CYM_T0_1,repls$CYM_T0_2,repls$CYM_T0_3
  ))
attr(repls,"dimnames")[[2]]=NULL
colnames(repls)=colnames(DF)
row.names(repls)=c(
  paste("CAU_T0_1",1:M,sep="_"),
  paste("CAU_T0_2",1:M,sep="_"),
  paste("CAU_T0_3",1:M,sep="_"),
  paste("CYM_T0_1",1:M,sep="_"),
  paste("CYM_T0_2",1:M,sep="_"),
  paste("CYM_T0_3",1:M,sep="_")
  )
mostres_ext=rbind(repls,easyCODA::CLR(DF)$LR)
conds_ext=as.factor(c(rep("CAU", 3*M),rep("CYM", 3*M),rep("CAU", 3),rep("CYM", 3)))
colors_ext=c("green","blue")[conds_ext]
rm(repls)
```

```
LogRat_mostres_ext_T0=logratios_matrix_clr(mostres_ext)
saveRDS(LogRat_mostres_ext_T0, file="LogRat_mostres_ext_T0.RData")
```

```
LogRat_mostres_ext_T0=readRDS("LogRat_mostres_ext_T0.RData")
LogRat_mostres_ext_T0=list(LogRat_mostres_ext_T0[[1]],LogRat_mostres_ext_T0[[2]])

coda.glmnet_ext=coda_glmnet_lr_bin(LogRat_mostres_ext_T0,conds_ext,taxa,showPlots = FALSE)
```

```
coda.glmnet_ext$`signature plot`
```

```
impGLMNET_ext=coda.glmnet_ext$taxa.num
DF.Imp=DF[,impGLMNET_ext]
HC=ClusterHC(DF.Imp,dendrograma=TRUE,barplot=FALSE,Grups=Type,colores=colors)
```

```
glmnet.ext=c(length(impGLMNET_ext),Sep(HC))
```

There are 104 significant taxa.  
The separation between groups is 4.42.

```
Significant=cbind(Significant,
glmnet_sign.ext.CAU=Indicador(coda.glmnet_ext$taxa.num[coda.glmnet_ext$`log-contrast coefficients`>0]),
glmnet_sign.ext.CYM=Indicador(coda.glmnet_ext$taxa.num[coda.glmnet_ext$`log-contrast coefficients`<0]))
```

#### 5.2.5 Simper

```
DF.prop=DF.0.total[c(1:3,13:15),]/rowSums(DF.0.total[c(1:3,13:15),])
SMP.prop=simper(DF.prop,group=Type)
```

```
indexos_SMP.07=SMP.prop$CAU_T2C_CAU_T2HW$ord[which(SMP.prop$CAU_T2C_CAU_T2HW$cusum<=0.7)]
indexos_SMP.05=SMP.prop$CAU_T2C_CAU_T2HW$ord[which(SMP.prop$CAU_T2C_CAU_T2HW$cusum<=0.5)]
length(indexos_SMP.07)
```

```
## [1] 0
```

```
length(indexos_SMP.05)
```

```
## [1] 0
```

We find no significant taxon.

#### 5.2.6 Summary

```
write.csv2(Significant,"OTU_Significativos_CAUT0vsCYMT0.csv",row.names=FALSE)
```

```
Resultados=rbind(
LR.max,
LR.max.2,
sep.kw.005,
glmnet.no0,
glmnet.ext)
row.names(Resultados)=c(
"LR.max",
"LR.max.local",
"Aldex",
"glmnet",
"glmnet.ext"
)
colnames(Resultados)=c("Size","Separation")
```

```
fit = smooth.spline(F.SS[,1],F.SS[,3],cv=TRUE)
fit.low = smooth.spline(F.SS[,1],F.SS[,4],cv=TRUE)
fit.up = smooth.spline(F.SS[,1],F.SS[,5],cv=TRUE)

plot(F.SS[,c(1,3)],pch=20,cex=0.25,col="darkgrey",xlab="n",ylab="Separation",main="Average separation for perfect classifiers",xlim=c(0,750),xaxp=c(0,750,15),ylim=c(0,7),yaxp=c(0,7,28))
lines(fit,col="black",lwd=1)
lines(fit.low,col="red",lwd=1)
lines(fit.up,col="red",lwd=1)
abline(h=separacio.global,col="green")

points(Resultados,col="blue",type="h")
points(Resultados,col="blue",pch=20)
text(Resultados[,1],Resultados[,2]+0.1,col="blue",labels=row.names(Resultados),cex=0.75)
text(20,separacio.global+0.01,col="green",labels="Global",cex=0.75)
```

Finally, we intersect the OTUs identified as significant according to LR and `coda_glmnet`.

```
Significant.capat=Significant[,c(1,2,4,6,7)]
Significant.capat=cbind(Significant.capat,Cuánto=rowSums(Significant.capat[,3:dim(Significant.capat)[2]]))
Significant.capat=Significant.capat[Significant.capat$Cuánto==2,]
```

```
DF.Imp=DF[,Significant.capat[,1]]
HC=ClusterHC(DF.Imp,dendrograma=TRUE,barplot=FALSE,Grups=Type,colores=colors)
```

There are 312 taxa. They are:

```
Significant.capat[,1:2]%>%
  kbl() %>%
  kable_styling()
```

|  | OTUs | taxa |
| --- | --- | --- |
| 1 | OTU1 | d\_\_Bacteria.p\_\_Proteobacteria.c\_\_Gammaproteobacteria.o\_\_Thiotrichales.f\_\_Thiotrichaceae.g**uncultured.** |
| 2 | OTU2 | d\_\_Bacteria.p\_\_Bacteroidota.c\_\_Bacteroidia.o\_\_Flavobacteriales.f\_\_Flavobacteriaceae.g\_\_Actibacter.s\_\_uncultured\_Bacteroidetes |
| 3 | OTU3 | d\_\_Bacteria.p\_\_Desulfobacterota.c\_\_Desulfobulbia.o\_\_Desulfobulbales.f\_\_Desulfocapsaceae.g\_\_uncultured.s\_\_uncultured\_delta |
| 4 | OTU4 | d\_\_Bacteria.p\_\_Bacteroidota.c\_\_Bacteroidia.o\_\_Flavobacteriales.f\_\_Flavobacteriaceae.g\_\_Robiginitalea.s\_\_bacterium\_AMSU |
| 7 | OTU7 | d\_\_Bacteria.p\_\_Proteobacteria.c\_\_Gammaproteobacteria.**.**.**.** |
| 8 | OTU8 | d\_\_Bacteria.p\_\_Chloroflexi.c\_\_Anaerolineae.o\_\_Anaerolineales.f\_\_Anaerolineaceae.g\_\_uncultured.s\_\_uncultured\_organism |
| 9 | OTU9 | d\_\_Bacteria.p\_\_Desulfobacterota.c\_\_Desulfobacteria.o\_\_Desulfobacterales.f\_\_Desulfosarcinaceae.g**Sva0081\_sediment\_group.** |
| 10 | OTU10 | d\_\_Bacteria.p\_\_Bacteroidota.c\_\_Bacteroidia.o\_\_Cytophagales.f\_\_Cyclobacteriaceae.g**uncultured.** |
| 12 | OTU12 | d\_\_Bacteria.p\_\_Bacteroidota.c\_\_Bacteroidia.o\_\_Bacteroidales.f\_\_Bacteroidetes\_BD2.2.g**Bacteroidetes\_BD2.2.** |
| 13 | OTU13 | d\_\_Bacteria.p\_\_Desulfobacterota.c\_\_Desulfobulbia.o\_\_Desulfobulbales.f\_\_Desulfocapsaceae.g**uncultured.** |
| 15 | OTU15 | d\_\_Bacteria.p\_\_Firmicutes.c\_\_Clostridia.o\_\_Clostridia.f\_\_Hungateiclostridiaceae.g\_\_uncultured.s\_\_uncultured\_bacterium |
| 16 | OTU16 | d\_\_Bacteria.p\_\_Desulfobacterota.c\_\_Desulfobacteria.o\_\_Desulfobacterales.f\_\_Desulfosarcinaceae.**.** |
| 18 | OTU18 | d\_\_Bacteria.p\_\_Proteobacteria.c\_\_Gammaproteobacteria.o\_\_Thiotrichales.f\_\_Thiotrichaceae.g\_\_uncultured.s\_\_uncultured\_sediment |
| 19 | OTU19 | d\_\_Bacteria.p\_\_Chloroflexi.c\_\_Anaerolineae.o\_\_Anaerolineales.f\_\_Anaerolineaceae.g**uncultured.** |
| 20 | OTU20 | d\_\_Bacteria.p\_\_Bacteroidota.c\_\_Bacteroidia.o\_\_Flavobacteriales.f\_\_Flavobacteriaceae.g\_\_Robiginitalea.s\_\_uncultured\_bacterium |
| 22 | OTU22 | d\_\_Bacteria.p\_\_Desulfobacterota.c\_\_Desulfobacteria.o\_\_Desulfobacterales.f\_\_Desulfosarcinaceae.g\_\_Desulfatitalea.s\_\_uncultured\_Desulfobacteraceae |
| 23 | OTU23 | d\_\_Bacteria.p\_\_Bacteroidota.c\_\_Bacteroidia.o\_\_Sphingobacteriales.f\_\_Lentimicrobiaceae.g**Lentimicrobiaceae.** |
| 24 | OTU24 | d\_\_Bacteria.p\_\_Bacteroidota.c\_\_Bacteroidia.o**Bacteroidales.**.**.** |
| 25 | OTU25 | d\_\_Archaea.p\_\_Asgardarchaeota.c\_\_Heimdallarchaeia.o\_\_Heimdallarchaeia.f\_\_Heimdallarchaeia.g\_\_Heimdallarchaeia.s\_\_uncultured\_archaeon |
| 26 | OTU26 | d\_\_Bacteria.p\_\_Chloroflexi.c\_\_Anaerolineae.o\_\_SBR1031.f\_\_SBR1031.g\_\_SBR1031.s\_\_uncultured\_organism |
| 28 | OTU28 | d\_\_Bacteria.p\_\_Proteobacteria.c\_\_Gammaproteobacteria.o\_\_Chromatiales.f\_\_Chromatiaceae.g\_\_Candidatus\_Thiobios.s\_\_uncultured\_sediment |
| 29 | OTU29 | d\_\_Bacteria.p\_\_Proteobacteria.c\_\_Gammaproteobacteria.o\_\_Milano.WF1B.44.f\_\_Milano.WF1B.44.g\_\_Milano.WF1B.44.s\_\_uncultured\_gamma |
| 30 | OTU30 | d\_\_Bacteria.p\_\_Bacteroidota.c\_\_Ignavibacteria.o\_\_Ignavibacteriales.f\_\_PHOS.HE36.g\_\_PHOS.HE36.s\_\_uncultured\_bacterium |
| 31 | OTU31 | d\_\_Bacteria.p\_\_Proteobacteria.c\_\_Gammaproteobacteria.o**Ectothiorhodospirales.**.**.** |
| 32 | OTU32 | d\_\_Bacteria.p\_\_Desulfobacterota.c\_\_Desulfobacteria.o\_\_Desulfobacterales.f\_\_Desulfosarcinaceae.g**uncultured.** |
| 33 | OTU33 | d\_\_Bacteria.p\_\_Bacteroidota.c\_\_Bacteroidia.o\_\_Bacteroidetes\_VC2.1\_Bac22.f\_\_Bacteroidetes\_VC2.1\_Bac22.g\_\_Bacteroidetes\_VC2.1\_Bac22.s\_\_uncultured\_Bacteroidetes |
| 35 | OTU35 | d\_\_Bacteria.p\_\_Desulfobacterota.c\_\_Desulfobacteria.o\_\_Desulfobacterales.f\_\_Desulfosarcinaceae.g\_\_LCP.80.s\_\_uncultured\_delta |
| 36 | OTU36 | d\_\_Bacteria.p\_\_Proteobacteria.c\_\_Gammaproteobacteria.o\_\_Steroidobacterales.f\_\_Woeseiaceae.g**Woeseia.** |
| 37 | OTU37 | d\_\_Bacteria.p\_\_Proteobacteria.c\_\_Gammaproteobacteria.o\_\_KI89A\_clade.f\_\_KI89A\_clade.g**KI89A\_clade.** |
| 38 | OTU38 | Unassigned.\_\_.**.**.**.**.\_\_ |
| 39 | OTU39 | d\_\_Bacteria.p\_\_Bacteroidota.c\_\_Bacteroidia.o\_\_Sphingobacteriales.f\_\_Lentimicrobiaceae.g\_\_Lentimicrobiaceae.s\_\_uncultured\_Bacteroidetes |
| 40 | OTU40 | d\_\_Bacteria.p\_\_Chloroflexi.c\_\_Anaerolineae.o\_\_Anaerolineales.f\_\_Anaerolineaceae.g\_\_Pelolinea.s\_\_uncultured\_bacterium |
| 41 | OTU41 | d\_\_Bacteria.p\_\_Proteobacteria.c\_\_Gammaproteobacteria.o\_\_B2M28.f\_\_B2M28.g**B2M28.** |
| 42 | OTU42 | d\_\_Bacteria.p\_\_Myxococcota.c\_\_Polyangia.o\_\_Polyangiales.f\_\_Sandaracinaceae.g**uncultured.** |
| 43 | OTU43 | d\_\_Bacteria.p\_\_Proteobacteria.c\_\_Gammaproteobacteria.o\_\_uncultured.f\_\_uncultured.g\_\_uncultured.s\_\_uncultured\_Thioalkalivibrio |
| 44 | OTU44 | d\_\_Bacteria.p\_\_Desulfobacterota.c\_\_Desulfobacteria.o\_\_Desulfobacterales.f\_\_Desulfosarcinaceae.g**SEEP.SRB1.** |
| 45 | OTU45 | d\_\_Bacteria.p\_\_Desulfobacterota.c\_\_Desulfobulbia.o\_\_Desulfobulbales.f\_\_Desulfocapsaceae.**.** |
| 47 | OTU47 | d\_\_Archaea.p\_\_Nanoarchaeota.c\_\_Nanoarchaeia.o\_\_Woesearchaeales.f\_\_SCGC\_AAA011.D5.g\_\_SCGC\_AAA011.D5.s\_\_uncultured\_euryarchaeote |
| 49 | OTU49 | d\_\_Bacteria.p\_\_Proteobacteria.c\_\_Gammaproteobacteria.o\_\_Chromatiales.f\_\_Sedimenticolaceae.g\_\_Candidatus\_Thiodiazotropha.s\_\_Candidatus\_Thiodiazotropha |
| 50 | OTU50 | d\_\_Bacteria.p\_\_Sva0485.c\_\_Sva0485.o\_\_Sva0485.f\_\_Sva0485.g**Sva0485.** |
| 52 | OTU52 | d\_\_Bacteria.p\_\_Actinobacteriota.c\_\_Acidimicrobiia.o\_\_Actinomarinales.f\_\_uncultured.g**uncultured.** |
| 53 | OTU53 | d\_\_Bacteria.p\_\_Proteobacteria.c\_\_Gammaproteobacteria.o\_\_uncultured.f\_\_uncultured.g**uncultured.** |
| 54 | OTU54 | d\_\_Bacteria.p\_\_Actinobacteriota.c\_\_Acidimicrobiia.o\_\_Microtrichales.f\_\_Ilumatobacteraceae.g**Ilumatobacter.** |
| 55 | OTU55 | d\_\_Bacteria.p\_\_Calditrichota.c\_\_Calditrichia.o\_\_Calditrichales.f\_\_Calditrichaceae.g\_\_Caldithrix.s\_\_uncultured\_bacterium |
| 56 | OTU56 | d\_\_Bacteria.p\_\_Proteobacteria.c\_\_Gammaproteobacteria.o\_\_Arenicellales.f\_\_Arenicellaceae.g**uncultured.** |
| 57 | OTU57 | d\_\_Bacteria.p\_\_Bacteroidota.c\_\_Bacteroidia.o\_\_Flavobacteriales.f\_\_Flavobacteriaceae.g**Eudoraea.** |
| 60 | OTU60 | d\_\_Bacteria.p\_\_Calditrichota.c\_\_Calditrichia.o\_\_Calditrichales.f\_\_Calditrichaceae.g\_\_Calditrichaceae.s\_\_uncultured\_bacterium |
| 62 | OTU62 | d\_\_Bacteria.p\_\_Proteobacteria.c\_\_Alphaproteobacteria.o\_\_Rhodobacterales.f\_\_Rhodobacteraceae.**.** |
| 64 | OTU64 | d\_\_Eukaryota.**.**.**.**.**.** |
| 69 | OTU69 | d\_\_Bacteria.p\_\_Proteobacteria.c\_\_Gammaproteobacteria.o\_\_Chromatiales.f\_\_Sedimenticolaceae.**.** |
| 70 | OTU70 | d\_\_Bacteria.p\_\_Desulfobacterota.c\_\_Desulfobacteria.o**Desulfobacterales.**.**.** |
| 71 | OTU71 | d\_\_Bacteria.p\_\_Bacteroidota.c\_\_Bacteroidia.o\_\_Flavobacteriales.f\_\_Flavobacteriaceae.g**Robiginitalea.** |
| 72 | OTU72 | d\_\_Bacteria.p\_\_Calditrichota.c\_\_Calditrichia.o\_\_Calditrichales.f\_\_Calditrichaceae.g\_\_Calorithrix.s\_\_uncultured\_bacterium |
| 73 | OTU73 | d\_\_Bacteria.p\_\_Bacteroidota.c\_\_Kryptonia.o\_\_Kryptoniales.f\_\_BSV26.g**BSV26.** |
| 77 | OTU77 | d\_\_Archaea.p\_\_Thermoplasmatota.c\_\_Thermoplasmata.o\_\_uncultured.f\_\_uncultured.g\_\_uncultured.s\_\_uncultured\_Thermoplasmatales |
| 79 | OTU79 | d\_\_Bacteria.p\_\_Acidobacteriota.c\_\_Subgroup\_21.o\_\_Subgroup\_21.f\_\_Subgroup\_21.g\_\_Subgroup\_21.s\_\_uncultured\_bacterium |
| 80 | OTU80 | d\_\_Bacteria.**.**.**.**.**.** |
| 81 | OTU81 | d\_\_Bacteria.p\_\_Latescibacterota.c\_\_Latescibacterota.o\_\_Latescibacterota.f\_\_Latescibacterota.g\_\_Latescibacterota.s\_\_uncultured\_bacterium |
| 82 | OTU82 | d\_\_Bacteria.p\_\_Calditrichota.c\_\_Calditrichia.o\_\_Calditrichales.f\_\_Calditrichaceae.g\_\_Caldithrix.s\_\_uncultured\_delta |
| 83 | OTU83 | d\_\_Bacteria.p\_\_SAR324\_clade.Marine\_group\_B..c\_\_SAR324\_clade.Marine\_group\_B..o\_\_SAR324\_clade.Marine\_group\_B..f\_\_SAR324\_clade.Marine\_group\_B..g**SAR324\_clade.Marine\_group\_B..** |
| 84 | OTU84 | d\_\_Bacteria.p\_\_Latescibacterota.c\_\_Latescibacteria.o\_\_Latescibacterales.f\_\_Latescibacteraceae.g\_\_Latescibacteraceae.s\_\_uncultured\_bacterium |
| 85 | OTU85 | d\_\_Bacteria.p**Chloroflexi.**.**.**.**.** |
| 88 | OTU88 | d\_\_Bacteria.p\_\_Spirochaetota.c\_\_Spirochaetia.o\_\_Spirochaetales.f\_\_Spirochaetaceae.g**Spirochaeta\_2.** |
| 94 | OTU94 | d\_\_Bacteria.p\_\_Spirochaetota.c\_\_Spirochaetia.o\_\_Spirochaetales.f\_\_Spirochaetaceae.g\_\_Spirochaeta.s\_\_uncultured\_bacterium |
| 95 | OTU95 | d\_\_Bacteria.p\_\_Cloacimonadota.c\_\_Cloacimonadia.o\_\_Cloacimonadales.f\_\_MSBL8.g\_\_MSBL8.s\_\_uncultured\_bacterium |
| 98 | OTU98 | d\_\_Bacteria.p\_\_Acetothermia.c\_\_Acetothermiia.o\_\_Acetothermiia.f\_\_Acetothermiia.g**Acetothermiia.** |
| 102 | OTU102 | d\_\_Bacteria.p\_\_Bacteroidota.c\_\_Ignavibacteria.o\_\_Ignavibacteriales.f\_\_Melioribacteraceae.g\_\_IheB3.7.s\_\_uncultured\_Chlorobi |
| 104 | OTU104 | d\_\_Bacteria.p\_\_Proteobacteria.c\_\_Alphaproteobacteria.o\_\_Rhizobiales.f\_\_Hyphomicrobiaceae.**.** |
| 105 | OTU105 | d\_\_Bacteria.p\_\_Proteobacteria.c\_\_Gammaproteobacteria.o\_\_Chromatiales.f\_\_Chromatiaceae.g**Halochromatium.** |
| 108 | OTU108 | d\_\_Bacteria.p\_\_Spirochaetota.c\_\_Spirochaetia.o\_\_Spirochaetales.f\_\_Spirochaetaceae.g**GWE2.31.10.** |
| 111 | OTU111 | d\_\_Bacteria.p\_\_Chloroflexi.c\_\_Anaerolineae.o\_\_Ardenticatenales.f\_\_uncultured.g**uncultured.** |
| 113 | OTU113 | d\_\_Archaea.p\_\_Altiarchaeota.c\_\_Altiarchaeia.o\_\_Altiarchaeales.f\_\_Altiarchaeaceae.g\_\_Candidatus\_Altiarchaeum.s\_\_uncultured\_archaeon |
| 116 | OTU116 | d\_\_Bacteria.p\_\_Zixibacteria.c\_\_Zixibacteria.o\_\_Zixibacteria.f\_\_Zixibacteria.g\_\_Zixibacteria.s\_\_uncultured\_bacterium |
| 118 | OTU118 | d\_\_Archaea.p\_\_Thermoplasmatota.c\_\_Thermoplasmata.o\_\_Marine\_Benthic\_Group\_D\_and\_DHVEG.1.f\_\_Marine\_Benthic\_Group\_D\_and\_DHVEG.1.g\_\_Marine\_Benthic\_Group\_D\_and\_DHVEG.1.s\_\_uncultured\_archaeon |
| 119 | OTU119 | d\_\_Bacteria.p\_\_Bacteroidota.c\_\_Bacteroidia.o\_\_Bacteroidales.f\_\_Marinilabiliaceae.g\_\_uncultured.s\_\_uncultured\_Bacteroidetes |
| 120 | OTU120 | d\_\_Bacteria.p\_\_Desulfobacterota.c\_\_Desulfobulbia.o\_\_Desulfobulbales.f\_\_Desulfobulbaceae.g**Desulfobulbus.** |
| 122 | OTU122 | d\_\_Bacteria.p\_\_Bacteroidota.c\_\_Bacteroidia.o\_\_Bacteroidales.f\_\_Marinilabiliaceae.g**uncultured.** |
| 123 | OTU123 | d\_\_Bacteria.p\_\_Planctomycetota.c\_\_Planctomycetes.o\_\_Pirellulales.f\_\_Pirellulaceae.g**Pir4\_lineage.** |
| 124 | OTU124 | d\_\_Bacteria.p\_\_Spirochaetota.c\_\_Spirochaetia.o\_\_Spirochaetales.f\_\_Spirochaetaceae.g\_\_GWE2.31.10.s\_\_uncultured\_gamma |
| 130 | OTU130 | d\_\_Bacteria.p\_\_Proteobacteria.c\_\_Alphaproteobacteria.o\_\_SAR11\_clade.f\_\_Clade\_III.g**Clade\_III.** |
| 132 | OTU132 | d\_\_Bacteria.p\_\_Proteobacteria.c\_\_Gammaproteobacteria.o\_\_BD7.8.f\_\_BD7.8.g**BD7.8.** |
| 133 | OTU133 | d\_\_Bacteria.p\_\_Desulfobacterota.c\_\_Desulfobacteria.o\_\_Desulfatiglandales.f\_\_Desulfatiglandaceae.g**Desulfatiglans.** |
| 135 | OTU135 | d\_\_Bacteria.p\_\_Proteobacteria.c\_\_Alphaproteobacteria.o\_\_SAR11\_clade.f\_\_Clade\_I.g**Clade\_Ia.** |
| 136 | OTU136 | d\_\_Bacteria.p\_\_Patescibacteria.c\_\_Parcubacteria.o\_\_Candidatus\_Kaiserbacteria.f\_\_Candidatus\_Kaiserbacteria.g\_\_Candidatus\_Kaiserbacteria.s\_\_uncultured\_bacterium |
| 139 | OTU139 | d\_\_Bacteria.p\_\_Proteobacteria.c\_\_Alphaproteobacteria.o\_\_Micavibrionales.f\_\_Micavibrionaceae.g\_\_uncultured.s\_\_uncultured\_bacterium |
| 140 | OTU140 | d\_\_Bacteria.p\_\_Cyanobacteria.c\_\_Cyanobacteriia.o\_\_Cyanobacteriales.f\_\_Cyanobacteriaceae.**.** |
| 144 | OTU144 | d\_\_Bacteria.p\_\_Bacteroidota.c\_\_Bacteroidia.o\_\_Flavobacteriales.f\_\_Flavobacteriaceae.g**Aquibacter.** |
| 146 | OTU146 | d\_\_Bacteria.p\_\_Spirochaetota.c\_\_Spirochaetia.o\_\_Spirochaetales.f\_\_Spirochaetaceae.g\_\_GWE2.31.10.s\_\_uncultured\_bacterium |
| 147 | OTU147 | d\_\_Bacteria.p\_\_Nitrospirota.c\_\_Thermodesulfovibrionia.o\_\_uncultured.f\_\_uncultured.g\_\_uncultured.s\_\_uncultured\_Nitrospirae |
| 148 | OTU148 | d\_\_Bacteria.p\_\_Verrucomicrobiota.c\_\_Kiritimatiellae.o\_\_Kiritimatiellales.f\_\_Kiritimatiellaceae.g**R76.B128.** |
| 149 | OTU149 | d\_\_Bacteria.p\_\_Acidobacteriota.c\_\_Subgroup\_18.o\_\_Subgroup\_18.f\_\_Subgroup\_18.g**Subgroup\_18.** |
| 150 | OTU150 | d\_\_Archaea.**.**.**.**.**.** |
| 152 | OTU152 | d\_\_Bacteria.p\_\_Verrucomicrobiota.c\_\_Verrucomicrobiae.o\_\_Pedosphaerales.f\_\_Pedosphaeraceae.g\_\_DEV008.s\_\_uncultured\_bacterium |
| 153 | OTU153 | d\_\_Archaea.p\_\_Thermoplasmatota.c\_\_Thermoplasmata.o\_\_uncultured.f\_\_uncultured.g\_\_uncultured.s\_\_uncultured\_archaeon |
| 155 | OTU155 | d\_\_Bacteria.p\_\_Bacteroidota.c\_\_Ignavibacteria.o\_\_Ignavibacteriales.f\_\_Melioribacteraceae.g\_\_IheB3.7.s\_\_uncultured\_sediment |
| 156 | OTU156 | d\_\_Bacteria.p\_\_Spirochaetota.c\_\_Spirochaetia.o\_\_Spirochaetales.f\_\_Spirochaetaceae.g\_\_Spirochaeta\_2.s\_\_uncultured\_bacterium |
| 157 | OTU157 | d\_\_Bacteria.p\_\_Desulfobacterota.c\_\_Desulfobacteria.o\_\_Desulfobacterales.f\_\_Desulfosarcinaceae.g\_\_SEEP.SRB1.s\_\_Olavius\_ilvae |
| 158 | OTU158 | d\_\_Bacteria.p\_\_Spirochaetota.c\_\_Spirochaetia.o\_\_Spirochaetales.f\_\_Spirochaetaceae.g\_\_Sediminispirochaeta.s\_\_uncultured\_bacterium |
| 159 | OTU159 | d\_\_Bacteria.p**Acidobacteriota.**.**.**.**.** |
| 160 | OTU160 | d\_\_Bacteria.p\_\_Bacteroidota.c\_\_Bacteroidia.o\_\_Chitinophagales.f\_\_Saprospiraceae.g\_\_uncultured.s\_\_uncultured\_Alphaproteobacteria |
| 161 | OTU161 | d\_\_Bacteria.p\_\_Chloroflexi.c\_\_Anaerolineae.o\_\_Caldilineales.f\_\_Caldilineaceae.g**uncultured.** |
| 162 | OTU162 | d\_\_Bacteria.p\_\_Gemmatimonadota.c\_\_PAUC43f\_marine\_benthic\_group.o\_\_PAUC43f\_marine\_benthic\_group.f\_\_PAUC43f\_marine\_benthic\_group.g\_\_PAUC43f\_marine\_benthic\_group.s\_\_uncultured\_actinobacterium |
| 168 | OTU168 | d\_\_Bacteria.p\_\_Proteobacteria.c\_\_Gammaproteobacteria.o\_\_Thiomicrospirales.f\_\_Thiomicrospiraceae.g**endosymbionts.** |
| 169 | OTU169 | d\_\_Bacteria.p\_\_Proteobacteria.c\_\_Gammaproteobacteria.o\_\_Gammaproteobacteria\_Incertae\_Sedis.f\_\_Unknown\_Family.g**uncultured.** |
| 173 | OTU173 | d\_\_Bacteria.p\_\_Calditrichota.c\_\_Calditrichia.o\_\_Calditrichales.f\_\_Calditrichaceae.g\_\_Caldithrix.s\_\_uncultured\_Deferribacteres |
| 175 | OTU175 | d\_\_Bacteria.p\_\_Proteobacteria.c\_\_Alphaproteobacteria.o\_\_Caulobacterales.f\_\_Hyphomonadaceae.**.** |
| 181 | OTU181 | d\_\_Bacteria.p\_\_Chloroflexi.c\_\_Anaerolineae.**.**.**.** |
| 185 | OTU185 | d\_\_Bacteria.p\_\_Patescibacteria.c\_\_WWE3.o\_\_WWE3.f\_\_WWE3.g\_\_WWE3.s\_\_uncultured\_bacterium |
| 189 | OTU189 | d\_\_Bacteria.p\_\_Firmicutes.c\_\_Clostridia.o\_\_Lachnospirales.f\_\_Defluviitaleaceae.g\_\_Defluviitaleaceae\_UCG.011.s\_\_uncultured\_bacterium |
| 190 | OTU190 | d\_\_Bacteria.p\_\_Myxococcota.c\_\_Polyangia.o\_\_Polyangiales.f\_\_Sandaracinaceae.g\_\_uncultured.s\_\_bacterium\_enrichment |
| 191 | OTU191 | d\_\_Archaea.p\_\_Nanoarchaeota.c\_\_Nanoarchaeia.o\_\_Woesearchaeales.f\_\_Woesearchaeales.g\_\_Woesearchaeales.s\_\_uncultured\_crenarchaeote |
| 192 | OTU192 | d\_\_Bacteria.p\_\_Planctomycetota.c\_\_Planctomycetes.o\_\_Pirellulales.f\_\_Pirellulaceae.g**Rhodopirellula.** |
| 194 | OTU194 | d\_\_Bacteria.p\_\_Spirochaetota.c\_\_Spirochaetia.o\_\_Spirochaetales.f\_\_Spirochaetaceae.g\_\_Spirochaeta\_2.s\_\_Spirochaeta\_isovalerica |
| 204 | OTU204 | d\_\_Bacteria.p\_\_Chloroflexi.c\_\_Anaerolineae.o\_\_SBR1031.f\_\_SBR1031.g\_\_SBR1031.s\_\_metagenome |
| 205 | OTU205 | d\_\_Bacteria.p\_\_Spirochaetota.c\_\_Spirochaetia.o\_\_Spirochaetales.f\_\_Spirochaetaceae.g**Sediminispirochaeta.** |
| 208 | OTU208 | d\_\_Bacteria.p\_\_Bacteroidota.c\_\_Bacteroidia.o\_\_Flavobacteriales.f\_\_Flavobacteriaceae.g**Lutibacter.** |
| 211 | OTU211 | d\_\_Bacteria.p\_\_Desulfobacterota.c\_\_Desulfobacteria.o\_\_Desulfobacterales.f\_\_Desulfosarcinaceae.g\_\_Desulfosarcina.s\_\_uncultured\_marine |
| 213 | OTU213 | d\_\_Bacteria.p\_\_Desulfobacterota.c\_\_Desulfobacteria.o\_\_Desulfobacterales.f\_\_Desulfosarcinaceae.g**Desulfatitalea.** |
| 216 | OTU216 | d\_\_Bacteria.p\_\_Actinobacteriota.c\_\_WCHB1.81.o\_\_WCHB1.81.f\_\_WCHB1.81.g\_\_WCHB1.81.s\_\_uncultured\_bacterium |
| 217 | OTU217 | d\_\_Bacteria.p\_\_Desulfobacterota.c\_\_Desulfobulbia.o\_\_Desulfobulbales.f\_\_Desulfobulbaceae.g\_\_Desulfobulbus.s\_\_uncultured\_bacterium |
| 222 | OTU222 | d\_\_Bacteria.p\_\_Bacteroidota.c\_\_Bacteroidia.o\_\_Bacteroidales.f\_\_Prolixibacteraceae.g**Sunxiuqinia.** |
| 226 | OTU226 | d\_\_Bacteria.p\_\_Chloroflexi.c\_\_Anaerolineae.o\_\_KZNMV.5.B42.f\_\_KZNMV.5.B42.g**KZNMV.5.B42.** |
| 228 | OTU228 | d\_\_Bacteria.p\_\_Verrucomicrobiota.c\_\_Lentisphaeria.o\_\_Oligosphaerales.f\_\_Lenti.02.g\_\_Lenti.02.s\_\_uncultured\_bacterium |
| 230 | OTU230 | d\_\_Bacteria.p\_\_Bacteroidota.c\_\_Bacteroidia.o\_\_Cytophagales.f\_\_Cyclobacteriaceae.g\_\_uncultured.s\_\_uncultured\_Bacteroidetes |
| 231 | OTU231 | d\_\_Bacteria.p\_\_Proteobacteria.c\_\_Gammaproteobacteria.o\_\_Chromatiales.f\_\_Chromatiaceae.**.** |
| 232 | OTU232 | d\_\_Bacteria.p\_\_Chloroflexi.c\_\_KD4.96.o\_\_KD4.96.f\_\_KD4.96.g**KD4.96.** |
| 236 | OTU236 | d\_\_Bacteria.p\_\_Bacteroidota.c\_\_Kryptonia.o\_\_Kryptoniales.f\_\_MSB.3C8.g**MSB.3C8.** |
| 237 | OTU237 | d\_\_Bacteria.p\_\_Proteobacteria.c\_\_Gammaproteobacteria.o\_\_Gammaproteobacteria\_Incertae\_Sedis.f\_\_Unknown\_Family.g\_\_uncultured.s\_\_uncultured\_marine |
| 239 | OTU239 | d\_\_Bacteria.p\_\_Zixibacteria.c\_\_Zixibacteria.o\_\_Zixibacteria.f\_\_Zixibacteria.g**Zixibacteria.** |
| 242 | OTU242 | d\_\_Bacteria.p\_\_Proteobacteria.c\_\_Gammaproteobacteria.o\_\_Gammaproteobacteria\_Incertae\_Sedis.f\_\_Unknown\_Family.g\_\_Unknown\_Family.s\_\_uncultured\_bacterium |
| 249 | OTU249 | d\_\_Bacteria.p\_\_Proteobacteria.c\_\_Gammaproteobacteria.o\_\_Chromatiales.f\_\_Sedimenticolaceae.g**uncultured.** |
| 252 | OTU252 | d\_\_Bacteria.p\_\_Verrucomicrobiota.c\_\_Verrucomicrobiae.o\_\_Verrucomicrobiales.f\_\_DEV007.g**DEV007.** |
| 253 | OTU253 | d\_\_Bacteria.p\_\_Desulfobacterota.c\_\_Desulfobacteria.o\_\_Desulfatiglandales.f\_\_Desulfatiglandaceae.g\_\_Desulfatiglans.s\_\_uncultured\_delta |
| 260 | OTU260 | d\_\_Bacteria.p\_\_Planctomycetota.c\_\_Phycisphaerae.o\_\_CCM11a.f\_\_CCM11a.g\_\_CCM11a.s\_\_uncultured\_organism |
| 266 | OTU266 | d\_\_Bacteria.p\_\_Patescibacteria.c\_\_ABY1.o\_\_Candidatus\_Kerfeldbacteria.f\_\_Candidatus\_Kerfeldbacteria.g\_\_Candidatus\_Kerfeldbacteria.s\_\_uncultured\_bacterium |
| 267 | OTU267 | d\_\_Bacteria.p\_\_Proteobacteria.c\_\_Alphaproteobacteria.o\_\_Caulobacterales.f\_\_Hyphomonadaceae.g**uncultured.** |
| 274 | OTU274 | d\_\_Bacteria.p\_\_Chloroflexi.c\_\_Anaerolineae.o\_\_Anaerolineales.f\_\_Anaerolineaceae.g\_\_Thermomarinilinea.s\_\_uncultured\_bacterium |
| 276 | OTU276 | d\_\_Bacteria.p\_\_Proteobacteria.c\_\_Alphaproteobacteria.**.**.**.** |
| 277 | OTU277 | d\_\_Bacteria.p\_\_Proteobacteria.c\_\_Alphaproteobacteria.o\_\_Kiloniellales.f\_\_Kiloniellaceae.g**Tistlia.** |
| 284 | OTU284 | d\_\_Bacteria.p\_\_LCP.89.c\_\_LCP.89.o\_\_LCP.89.f\_\_LCP.89.g\_\_LCP.89.s\_\_saltmarsh\_clone |
| 292 | OTU292 | d\_\_Archaea.p\_\_Thermoplasmatota.c\_\_Thermoplasmatota.o\_\_Thermoplasmatota.f\_\_Thermoplasmatota.g\_\_uncultured.s\_\_uncultured\_archaeon |
| 293 | OTU293 | d\_\_Archaea.p\_\_Aenigmarchaeota.c\_\_Deep\_Sea\_Euryarchaeotic\_Group.DSEG..o\_\_Deep\_Sea\_Euryarchaeotic\_Group.DSEG..f\_\_Deep\_Sea\_Euryarchaeotic\_Group.DSEG..g\_\_Deep\_Sea\_Euryarchaeotic\_Group.DSEG..s\_\_uncultured\_archaeon |
| 300 | OTU300 | d\_\_Bacteria.p\_\_Spirochaetota.c\_\_Spirochaetia.o\_\_Spirochaetales.f\_\_Spirochaetaceae.g\_\_Sediminispirochaeta.s\_\_uncultured\_Spirochaetes |
| 304 | OTU304 | d\_\_Bacteria.p\_\_Bacteroidota.c\_\_Bacteroidia.o\_\_Flavobacteriales.f\_\_Flavobacteriaceae.g**Maritimimonas.** |
| 305 | OTU305 | d\_\_Bacteria.p\_\_Bacteroidota.c\_\_Bacteroidia.o\_\_Chitinophagales.f\_\_Saprospiraceae.g\_\_uncultured.s\_\_uncultured\_organism |
| 307 | OTU307 | d\_\_Archaea.p\_\_Nanoarchaeota.c\_\_Nanoarchaeia.o**Woesearchaeales.**.**.** |
| 314 | OTU314 | d\_\_Bacteria.p\_\_Proteobacteria.c\_\_Gammaproteobacteria.o\_\_UBA10353\_marine\_group.f\_\_UBA10353\_marine\_group.g\_\_UBA10353\_marine\_group.s\_\_uncultured\_bacterium |
| 318 | OTU318 | d\_\_Bacteria.p\_\_Proteobacteria.c\_\_Alphaproteobacteria.o\_\_Rhizobiales.f\_\_Hyphomicrobiaceae.g\_\_Filomicrobium.s\_\_uncultured\_Alphaproteobacteria |
| 328 | OTU328 | d\_\_Bacteria.p\_\_Desulfobacterota.c\_\_Desulfobacteria.o\_\_Desulfobacterales.f\_\_Desulfosarcinaceae.g\_\_Desulfatirhabdium.s\_\_uncultured\_delta |
| 334 | OTU334 | d\_\_Bacteria.p\_\_Planctomycetota.c\_\_Phycisphaerae.o\_\_MSBL9.f\_\_SG8.4.g**SG8.4.** |
| 337 | OTU337 | d\_\_Bacteria.p\_\_Chloroflexi.c\_\_Anaerolineae.o\_\_SJA.15.f\_\_SJA.15.g\_\_SJA.15.s\_\_uncultured\_Chloroflexi |
| 340 | OTU340 | d\_\_Bacteria.p\_\_Patescibacteria.c\_\_Gracilibacteria.o\_\_Absconditabacteriales\_.SR1..f\_*Absconditabacteriales*.SR1..g\_*Absconditabacteriales*.SR1..s\_\_uncultured\_bacterium |
| 341 | OTU341 | d\_\_Bacteria.p\_\_Bacteroidota.c\_\_Bacteroidia.o\_\_Flavobacteriales.f\_\_Crocinitomicaceae.g\_\_Crocinitomix.s\_\_uncultured\_bacterium |
| 353 | OTU353 | d\_\_Bacteria.p\_\_Proteobacteria.c\_\_Gammaproteobacteria.o\_\_Oceanospirillales.f\_\_Nitrincolaceae.g**uncultured.** |
| 354 | OTU354 | d\_\_Bacteria.p\_\_Spirochaetota.c\_\_Spirochaetia.o\_\_Spirochaetales.f\_\_Spirochaetaceae.g\_\_Spirochaeta\_2.s\_\_uncultured\_Spirochaetes |
| 356 | OTU356 | d\_\_Bacteria.p\_\_Planctomycetota.c\_\_Phycisphaerae.o\_\_CCM11a.f\_\_CCM11a.g\_\_CCM11a.s\_\_uncultured\_bacterium |
| 358 | OTU358 | d\_\_Bacteria.p\_\_Elusimicrobiota.c\_\_Lineage\_IIb.o\_\_Lineage\_IIb.f\_\_Lineage\_IIb.g\_\_Lineage\_IIb.s\_\_uncultured\_bacterium |
| 368 | OTU368 | d\_\_Bacteria.p\_\_Proteobacteria.c\_\_Alphaproteobacteria.o\_\_Rhodobacterales.f\_\_Rhodobacteraceae.g**HIMB11.** |
| 371 | OTU371 | d\_\_Bacteria.p\_\_Bacteroidota.c\_\_Bacteroidia.o\_\_Flavobacteriales.f\_\_Flavobacteriaceae.g\_\_Maritimimonas.s\_\_uncultured\_organism |
| 372 | OTU372 | d\_\_Bacteria.p\_\_Proteobacteria.c\_\_Gammaproteobacteria.o\_\_Oceanospirillales.f\_\_Pseudohongiellaceae.g**Pseudohongiella.** |
| 381 | OTU381 | d\_\_Bacteria.p\_\_Patescibacteria.c\_\_Gracilibacteria.o\_\_Candidatus\_Peregrinibacteria.f\_\_Candidatus\_Peregrinibacteria.g\_\_Candidatus\_Peregrinibacteria.s\_\_uncultured\_bacterium |
| 386 | OTU386 | d\_\_Bacteria.p\_\_Proteobacteria.c\_\_Gammaproteobacteria.o\_\_Vibrionales.f\_\_Vibrionaceae.g**Vibrio.** |
| 389 | OTU389 | d\_\_Bacteria.p\_\_Bdellovibrionota.c\_\_Bdellovibrionia.o\_\_Bacteriovoracales.f\_\_Bacteriovoracaceae.g\_\_Peredibacter.s\_\_uncultured\_bacterium |
| 390 | OTU390 | d\_\_Bacteria.p\_\_Latescibacterota.c\_\_Latescibacterota.o\_\_Latescibacterota.f\_\_Latescibacterota.g\_\_Latescibacterota.s\_\_uncultured\_organism |
| 392 | OTU392 | d\_\_Archaea.p\_\_Asgardarchaeota.c\_\_Odinarchaeia.o\_\_Odinarchaeia.f\_\_Odinarchaeia.g\_\_Odinarchaeia.s\_\_uncultured\_marine |
| 393 | OTU393 | d\_\_Bacteria.p\_\_Latescibacterota.c\_\_Latescibacteria.o\_\_Latescibacterales.f\_\_Latescibacteraceae.g\_\_Latescibacteraceae.s\_\_uncultured\_actinobacterium |
| 394 | OTU394 | d\_\_Bacteria.p\_\_Desulfobacterota.c\_\_Desulfobacteria.o\_\_Desulfobacterales.f\_\_Desulfosarcinaceae.g\_\_Sva0081\_sediment\_group.s\_\_uncultured\_marine |
| 397 | OTU397 | d\_\_Bacteria.p\_\_NB1.j.c\_\_NB1.j.o\_\_NB1.j.f\_\_NB1.j.g**NB1.j.** |
| 400 | OTU400 | d\_\_Bacteria.p\_\_Planctomycetota.c\_\_Phycisphaerae.o\_\_CCM11a.f\_\_CCM11a.g**CCM11a.** |
| 401 | OTU401 | d\_\_Bacteria.p\_\_Bacteroidota.c\_\_Bacteroidia.o\_\_Bacteroidales.f\_\_Marinilabiliaceae.g\_\_Labilibacter.s\_\_uncultured\_Bacteroidetes |
| 402 | OTU402 | d\_\_Bacteria.p\_\_Acetothermia.c\_\_Acetothermiia.o\_\_Acetothermiia.f\_\_Acetothermiia.g\_\_Acetothermiia.s\_\_uncultured\_organism |
| 421 | OTU421 | d\_\_Bacteria.p\_\_Proteobacteria.c\_\_Alphaproteobacteria.o\_\_Micavibrionales.f\_\_uncultured.g**uncultured.** |
| 424 | OTU424 | d\_\_Bacteria.p\_\_Gemmatimonadota.c\_\_PAUC43f\_marine\_benthic\_group.o\_\_PAUC43f\_marine\_benthic\_group.f\_\_PAUC43f\_marine\_benthic\_group.g**PAUC43f\_marine\_benthic\_group.** |
| 426 | OTU426 | d\_\_Bacteria.p\_\_Verrucomicrobiota.c\_\_Omnitrophia.o\_\_Omnitrophales.f\_\_Omnitrophaceae.g\_\_Candidatus\_Omnitrophus.s\_\_uncultured\_bacterium |
| 435 | OTU435 | d\_\_Bacteria.p\_\_Desulfobacterota.c\_\_Desulfobacteria.o\_\_Desulfobacterales.f\_\_Desulfobacteraceae.g**Desulfobacter.** |
| 436 | OTU436 | d\_\_Bacteria.p\_\_Desulfobacterota.c\_\_Desulfuromonadia.o\_\_Bradymonadales.f\_\_Bradymonadales.g**Bradymonadales.** |
| 448 | OTU448 | d\_\_Bacteria.p\_\_Patescibacteria.c\_\_ABY1.o\_\_Candidatus\_Falkowbacteria.f\_\_Candidatus\_Falkowbacteria.g**Candidatus\_Falkowbacteria.** |
| 453 | OTU453 | d\_\_Bacteria.p\_\_Modulibacteria.c\_\_Moduliflexia.o\_\_Moduliflexales.f\_\_Moduliflexaceae.g**Moduliflexaceae.** |
| 463 | OTU463 | d\_\_Bacteria.p\_\_Chloroflexi.c\_\_Anaerolineae.o\_\_RBG.13.54.9.f\_\_RBG.13.54.9.g\_\_RBG.13.54.9.s\_\_uncultured\_bacterium |
| 475 | OTU475 | d\_\_Bacteria.p\_\_Chloroflexi.c\_\_Dehalococcoidia.o\_\_GIF9.f\_\_AB.539.J10.g\_\_SCGC.AB.539.J10.s\_\_uncultured\_bacterium |
| 476 | OTU476 | d\_\_Bacteria.p\_\_Myxococcota.c\_\_Polyangia.o\_\_MidBa8.f\_\_MidBa8.g\_\_MidBa8.s\_\_uncultured\_delta |
| 480 | OTU480 | d\_\_Bacteria.p\_\_Calditrichota.c\_\_Calditrichia.o\_\_Calditrichales.f\_\_Calditrichaceae.g\_\_Calditrichaceae.s\_\_uncultured\_organism |
| 482 | OTU482 | d\_\_Bacteria.p\_\_Cyanobacteria.c\_\_Cyanobacteriia.o\_\_Chloroplast.f\_\_Chloroplast.g\_\_Chloroplast.s\_\_Chromerida\_sp. |
| 483 | OTU483 | d\_\_Bacteria.p\_\_Bdellovibrionota.c\_\_Oligoflexia.o\_\_Oligoflexales.f\_\_uncultured.g\_\_uncultured.s\_\_uncultured\_organism |
| 490 | OTU490 | d\_\_Bacteria.p\_\_Bdellovibrionota.c\_\_Bdellovibrionia.o\_\_Bacteriovoracales.f\_\_Bacteriovoracaceae.g**Peredibacter.** |
| 495 | OTU495 | d\_\_Archaea.p\_\_Micrarchaeota.c\_\_Micrarchaeia.o\_\_Micrarchaeales.f\_\_Micrarchaeales.g\_\_Micrarchaeales.s\_\_uncultured\_euryarchaeote |
| 501 | OTU501 | d\_\_Bacteria.p\_\_Latescibacterota.c\_\_Latescibacterota.o\_\_Latescibacterota.f\_\_Latescibacterota.g\_\_Latescibacterota.s\_\_uncultured\_Bacteroidetes |
| 503 | OTU503 | d\_\_Bacteria.p\_\_Fibrobacterota.c\_\_Chitinivibrionia.o\_\_Chitinivibrionales.f\_\_Chitinivibrionaceae.g\_\_possible\_genus\_03.s\_\_uncultured\_bacterium |
| 508 | OTU508 | d\_\_Bacteria.p\_\_Verrucomicrobiota.c\_\_Verrucomicrobiae.o\_\_Verrucomicrobiales.f\_\_DEV007.g\_\_DEV007.s\_\_uncultured\_organism |
| 509 | OTU509 | d\_\_Bacteria.p\_\_Proteobacteria.c\_\_Alphaproteobacteria.o\_\_Rhodospirillales.f\_\_Rhodospirillaceae.g\_\_Candidatus\_Riegeria.s\_\_uncultured\_bacterium |
| 530 | OTU530 | d\_\_Bacteria.p\_\_Proteobacteria.c\_\_Gammaproteobacteria.o\_\_Thiomicrospirales.f\_\_Thioglobaceae.g**SUP05\_cluster.** |
| 532 | OTU532 | d\_\_Bacteria.p\_\_Acetothermia.c\_\_Acetothermiia.o\_\_Acetothermiia.f\_\_Acetothermiia.g\_\_Acetothermiia.s\_\_bacterium\_enrichment |
| 537 | OTU537 | d\_\_Bacteria.p\_\_Fermentibacterota.c\_\_Fermentibacteria.o\_\_Fermentibacterales.f\_\_Fermentibacteraceae.g\_\_Fermentibacteraceae.s\_\_uncultured\_sediment |
| 539 | OTU539 | d\_\_Bacteria.p\_\_Patescibacteria.c\_\_Parcubacteria.o\_\_Candidatus\_Kaiserbacteria.f\_\_Candidatus\_Kaiserbacteria.g**Candidatus\_Kaiserbacteria.** |
| 541 | OTU541 | d\_\_Bacteria.p\_\_Proteobacteria.c\_\_Gammaproteobacteria.o\_\_Ga0077536.f\_\_Ga0077536.g\_\_Ga0077536.s\_\_uncultured\_gamma |
| 547 | OTU547 | d\_\_Bacteria.p\_\_Proteobacteria.c\_\_Alphaproteobacteria.o\_\_Rhizobiales.f\_\_Rhizobiales\_Incertae\_Sedis.g**Anderseniella.** |
| 553 | OTU553 | d\_\_Bacteria.p\_\_Campilobacterota.c\_\_Campylobacteria.o\_\_Campylobacterales.f\_\_Helicobacteraceae.**.** |
| 554 | OTU554 | d\_\_Bacteria.p\_\_Chloroflexi.c\_\_Dehalococcoidia.o\_\_GIF9.f\_\_AB.539.J10.g\_\_AB.539.J10.s\_\_uncultured\_bacterium |
| 565 | OTU565 | d\_\_Bacteria.p\_\_Bacteroidota.c\_\_Bacteroidia.o\_\_Chitinophagales.f\_\_Saprospiraceae.g\_\_Portibacter.s\_\_uncultured\_Bacteroidetes |
| 568 | OTU568 | d\_\_Bacteria.p\_\_Margulisbacteria.c\_\_Margulisbacteria.o\_\_Margulisbacteria.f\_\_Margulisbacteria.g\_\_Margulisbacteria.s\_\_uncultured\_bacterium |
| 570 | OTU570 | d\_\_Bacteria.p\_\_Chloroflexi.c\_\_Anaerolineae.o\_\_Thermoflexales.f\_\_Thermoflexaceae.g**Thermoflexus.** |
| 574 | OTU574 | d\_\_Bacteria.p\_\_Planctomycetota.c\_\_Planctomycetes.o\_\_Pirellulales.f\_\_Pirellulaceae.g\_\_uncultured.s\_\_uncultured\_organism |
| 585 | OTU585 | d\_\_Bacteria.p\_\_Bacteroidota.c\_\_Kryptonia.o\_\_Kryptoniales.f\_\_MSB.3C8.g\_\_MSB.3C8.s\_\_uncultured\_bacterium |
| 618 | OTU618 | d\_\_Bacteria.p\_\_Proteobacteria.c\_\_Gammaproteobacteria.o\_\_Ectothiorhodospirales.f\_\_Ectothiorhodospiraceae.g\_\_Thiogranum.s\_\_uncultured\_gamma |
| 620 | OTU620 | d\_\_Bacteria.p\_\_Firmicutes.c\_\_Clostridia.o\_\_Lachnospirales.f\_\_Lachnospiraceae.g\_\_uncultured.s\_\_Clostridiales\_bacterium |
| 622 | OTU622 | d\_\_Bacteria.p\_\_Bacteroidota.c\_\_Bacteroidia.o\_\_Chitinophagales.f\_\_uncultured.g\_\_uncultured.s\_\_uncultured\_Bacteroidetes |
| 628 | OTU628 | d\_\_Bacteria.p\_\_Campilobacterota.c\_\_Campylobacteria.o\_\_Campylobacterales.f\_\_Rs.M59\_termite\_group.g\_\_Rs.M59\_termite\_group.s\_\_uncultured\_bacterium |
| 631 | OTU631 | d\_\_Bacteria.p\_\_Campilobacterota.c\_\_Campylobacteria.o\_\_Campylobacterales.f\_\_Campylobacteraceae.g\_\_Campylobacter.s\_\_uncultured\_Epsilonproteobacteria |
| 657 | OTU657 | d\_\_Bacteria.p\_\_Actinobacteriota.c\_\_Coriobacteriia.o\_\_OPB41.f\_\_OPB41.g\_\_OPB41.s\_\_unidentified\_marine |
| 663 | OTU663 | d\_\_Bacteria.p\_\_Acidobacteriota.c\_\_Vicinamibacteria.o\_\_Subgroup\_9.f\_\_Subgroup\_9.g\_\_Subgroup\_9.s\_\_uncultured\_bacterium |
| 694 | OTU694 | d\_\_Bacteria.p\_\_Bacteroidota.c\_\_Bacteroidia.o\_\_Flavobacteriales.f\_\_Flavobacteriaceae.g\_\_NS5\_marine\_group.s\_\_uncultured\_Flavobacterium |
| 700 | OTU700 | d\_\_Bacteria.p\_\_SAR324\_clade.Marine\_group\_B..c\_\_SAR324\_clade.Marine\_group\_B..o\_\_SAR324\_clade.Marine\_group\_B..f\_\_SAR324\_clade.Marine\_group\_B..g\_\_SAR324\_clade.Marine\_group\_B..s\_\_uncultured\_organism |
| 706 | OTU706 | d\_\_Bacteria.p\_\_Myxococcota.c\_\_Polyangia.o\_\_UASB.TL25.f\_\_UASB.TL25.g**UASB.TL25.** |
| 708 | OTU708 | d\_\_Bacteria.p\_\_Patescibacteria.c\_\_Gracilibacteria.o\_\_Gracilibacteria.f\_\_Gracilibacteria.g\_\_Gracilibacteria.s\_\_uncultured\_organism |
| 714 | OTU714 | d\_\_Bacteria.p\_\_Verrucomicrobiota.c\_\_Omnitrophia.o\_\_Omnitrophales.f\_\_Omnitrophales.g\_\_Omnitrophales.s\_\_uncultured\_bacterium |
| 729 | OTU729 | d\_\_Bacteria.p\_\_Patescibacteria.c\_\_Parcubacteria.o\_\_Candidatus\_Kaiserbacteria.f\_\_Candidatus\_Kaiserbacteria.g\_\_Candidatus\_Kaiserbacteria.s\_\_uncultured\_organism |
| 742 | OTU742 | d\_\_Bacteria.p\_\_Planctomycetota.c\_\_Pla4\_lineage.o\_\_Pla4\_lineage.f\_\_Pla4\_lineage.g\_\_Pla4\_lineage.s\_\_uncultured\_bacterium |
| 750 | OTU750 | d\_\_Bacteria.p\_\_Desulfobacterota.c\_\_Syntrophobacteria.o\_\_Syntrophobacterales.f\_\_uncultured.g\_\_uncultured.s\_\_uncultured\_delta |
| 761 | OTU761 | d\_\_Bacteria.p\_\_Actinobacteriota.c\_\_Acidimicrobiia.o\_\_Microtrichales.f\_\_Microtrichaceae.g**Sva0996\_marine\_group.** |
| 776 | OTU776 | d\_\_Bacteria.p\_\_Proteobacteria.c\_\_Alphaproteobacteria.o\_\_Rhodospirillales.f\_\_Terasakiellaceae.g**uncultured.** |
| 789 | OTU789 | d\_\_Bacteria.p\_\_Desulfobacterota.c\_\_Desulfobulbia.o\_\_Desulfobulbales.f\_\_Desulfocapsaceae.g\_\_Desulfopila.s\_\_uncultured\_bacterium |
| 794 | OTU794 | d\_\_Bacteria.p\_\_Proteobacteria.c\_\_Alphaproteobacteria.o\_\_Rhodobacterales.f\_\_Rhodobacteraceae.g**Marivita.** |
| 803 | OTU803 | d\_\_Archaea.p\_\_Aenigmarchaeota.c\_\_Aenigmarchaeia.o\_\_Aenigmarchaeales.f\_\_Aenigmarchaeales.g**Aenigmarchaeales.** |
| 814 | OTU814 | d\_\_Archaea.p\_\_Aenigmarchaeota.c\_\_Aenigmarchaeia.o\_\_Aenigmarchaeales.f\_\_Aenigmarchaeales.g\_\_Aenigmarchaeales.s\_\_uncultured\_archaeon |
| 823 | OTU823 | d\_\_Bacteria.p\_\_WS2.c\_\_WS2.o\_\_WS2.f\_\_WS2.g\_\_WS2.s\_\_uncultured\_Firmicutes |
| 837 | OTU837 | d\_\_Bacteria.p\_\_Latescibacterota.c\_\_Latescibacteria.o\_\_Latescibacterales.f\_\_Latescibacteraceae.g\_\_Latescibacteraceae.s\_\_bacterium\_enrichment |
| 840 | OTU840 | d\_\_Bacteria.p\_\_Chloroflexi.c\_\_Dehalococcoidia.o\_\_SAR202\_clade.f\_\_SAR202\_clade.g**SAR202\_clade.** |
| 845 | OTU845 | d\_\_Bacteria.p\_\_Dependentiae.c\_\_Babeliae.o\_\_Babeliales.f\_\_Vermiphilaceae.g**Vermiphilaceae.** |
| 897 | OTU897 | d\_\_Bacteria.p\_\_Chloroflexi.c\_\_Dehalococcoidia.o\_\_GIF9.f\_\_AB.539.J10.g**AB.539.J10.** |
| 905 | OTU905 | d\_\_Bacteria.p\_\_Proteobacteria.c\_\_Gammaproteobacteria.o\_\_Granulosicoccales.f\_\_Granulosicoccaceae.g\_\_Granulosicoccus.s\_\_uncultured\_bacterium |
| 908 | OTU908 | d\_\_Bacteria.p\_\_Verrucomicrobiota.c\_\_Verrucomicrobiae.o**Opitutales.**.**.** |
| 933 | OTU933 | d\_\_Bacteria.p\_\_Campilobacterota.c\_\_Campylobacteria.o\_\_Campylobacterales.f\_\_Sulfurimonadaceae.g\_\_Sulfurimonas.s\_\_bacterium\_enrichment |
| 937 | OTU937 | d\_\_Bacteria.p\_\_Acidobacteriota.c\_\_Acidobacteriae.**.**.**.** |
| 950 | OTU950 | d\_\_Bacteria.p\_\_Proteobacteria.c\_\_Gammaproteobacteria.o\_\_CH2b56.f\_\_CH2b56.g\_\_CH2b56.s\_\_uncultured\_bacterium |
| 954 | OTU954 | d\_\_Bacteria.p\_\_Planctomycetota.c\_\_vadinHA49.o\_\_vadinHA49.f\_\_vadinHA49.g\_\_vadinHA49.s\_\_uncultured\_organism |
| 967 | OTU967 | d\_\_Bacteria.p\_\_Planctomycetota.c\_\_Pla3\_lineage.o\_\_Pla3\_lineage.f\_\_Pla3\_lineage.g\_\_Pla3\_lineage.s\_\_uncultured\_planctomycete |
| 983 | OTU983 | d\_\_Bacteria.p\_\_Desulfobacterota.c\_\_Desulfobulbia.o\_\_Desulfobulbales.f\_\_Desulfobulbaceae.g\_\_uncultured.s\_\_uncultured\_gamma |
| 987 | OTU987 | d\_\_Bacteria.p\_\_Desulfobacterota.c\_\_Desulfobacteria.o\_\_Desulfobacterales.f\_\_Desulfobacteraceae.g\_\_uncultured.s\_\_uncultured\_organism |
| 988 | OTU988 | d\_\_Bacteria.p\_\_Proteobacteria.c\_\_Alphaproteobacteria.o\_\_Sphingomonadales.f\_\_Sphingomonadaceae.g\_\_Erythrobacter.s\_\_Erythrobacter\_longus |
| 989 | OTU989 | d\_\_Bacteria.p\_\_Bacteroidota.c\_\_Bacteroidia.o\_\_Chitinophagales.f\_\_Saprospiraceae.g\_\_Lewinella.s\_\_uncultured\_organism |
| 991 | OTU991 | d\_\_Bacteria.p\_\_Acetothermia.c\_\_Acetothermiia.o\_\_Acetothermiia.f\_\_Acetothermiia.g\_\_Acetothermiia.s\_\_uncultured\_bacterium |
| 993 | OTU993 | d\_\_Bacteria.p\_\_Sumerlaeota.c\_\_Sumerlaeia.o\_\_Sumerlaeales.f\_\_Sumerlaeaceae.g\_\_Sumerlaea.s\_\_uncultured\_organism |
| 998 | OTU998 | d\_\_Bacteria.p\_\_Bacteroidota.c\_\_Bacteroidia.o\_\_Flavobacteriales.f\_\_Flavobacteriaceae.g\_\_Flavobacteriaceae.s\_\_Aureicoccus\_marinus |
| 1000 | OTU1000 | d\_\_Bacteria.p\_\_Cyanobacteria.c\_\_Cyanobacteriia.o\_\_Synechococcales.f\_\_Cyanobiaceae.g**Cyanobium\_PCC.6307.** |
| 1001 | OTU1001 | d\_\_Bacteria.p\_\_Proteobacteria.c\_\_Alphaproteobacteria.o\_\_Paracaedibacterales.f\_\_Paracaedibacteraceae.g\_\_Candidatus\_Captivus.s\_\_uncultured\_bacterium |
| 1003 | OTU1003 | d\_\_Bacteria.p\_\_Bacteroidota.c\_\_Bacteroidia.o\_\_Sphingobacteriales.f\_\_NS11.12\_marine\_group.g\_\_NS11.12\_marine\_group.s\_\_uncultured\_marine |
| 1013 | OTU1013 | d\_\_Archaea.p\_\_Nanoarchaeota.c\_\_Nanoarchaeia.o\_\_Woesearchaeales.f\_\_GW2011\_GWC1\_47\_15.g**GW2011\_GWC1\_47\_15.** |
| 1014 | OTU1014 | d\_\_Bacteria.p\_\_Proteobacteria.c\_\_Gammaproteobacteria.o**Cellvibrionales.**.**.** |
| 1041 | OTU1041 | d\_\_Bacteria.p\_\_Verrucomicrobiota.c\_\_Verrucomicrobiae.o\_\_Verrucomicrobiales.f\_\_Rubritaleaceae.g\_\_uncultured.s\_\_uncultured\_bacterium |
| 1050 | OTU1050 | d\_\_Bacteria.p\_\_Bacteroidota.c\_\_Bacteroidia.o\_\_Chitinophagales.f\_\_uncultured.g\_\_uncultured.s\_\_uncultured\_bacterium |
| 1057 | OTU1057 | d\_\_Bacteria.p\_\_Dependentiae.c\_\_Babeliae.o\_\_Babeliales.f\_\_Babeliales.g\_\_Babeliales.s\_\_uncultured\_organism |
| 1064 | OTU1064 | d\_\_Bacteria.p\_\_Dependentiae.c\_\_Babeliae.o\_\_Babeliales.f\_\_Vermiphilaceae.g\_\_Vermiphilaceae.s\_\_uncultured\_delta |
| 1070 | OTU1070 | d\_\_Bacteria.p\_\_Cyanobacteria.c\_\_Vampirivibrionia.o\_\_Gastranaerophilales.f\_\_Gastranaerophilales.g**Gastranaerophilales.** |
| 1086 | OTU1086 | d\_\_Bacteria.p\_\_Planctomycetota.c\_\_Planctomycetes.o\_\_Pirellulales.f\_\_Pirellulaceae.g**Rubripirellula.** |
| 1097 | OTU1097 | d\_\_Bacteria.p\_\_Latescibacterota.c\_\_Latescibacterota.o\_\_Latescibacterota.f\_\_Latescibacterota.g\_\_Latescibacterota.s\_\_unidentified\_bacterium |
| 1100 | OTU1100 | d\_\_Bacteria.p\_\_Bacteroidota.c\_\_Bacteroidia.o\_\_Cytophagales.f\_\_Cyclobacteriaceae.g\_\_uncultured.s\_\_uncultured\_soil |
| 1108 | OTU1108 | d\_\_Bacteria.p\_\_NB1.j.c\_\_NB1.j.o\_\_NB1.j.f\_\_NB1.j.g\_\_NB1.j.s\_\_uncultured\_prokaryote |
| 1114 | OTU1114 | d\_\_Bacteria.p\_\_Verrucomicrobiota.c\_\_Lentisphaeria.o\_\_SS1.B.02.17.f\_\_SS1.B.02.17.g\_\_SS1.B.02.17.s\_\_uncultured\_Lentisphaerae |
| 1121 | OTU1121 | d\_\_Bacteria.p\_\_Myxococcota.c\_\_Polyangia.o\_\_Polyangiales.f\_\_Eel.36e1D6.g\_\_Eel.36e1D6.s\_\_uncultured\_Polyangiaceae |
| 1127 | OTU1127 | d\_\_Bacteria.p\_\_Actinobacteriota.c\_\_WCHB1.81.o\_\_WCHB1.81.f\_\_WCHB1.81.g\_\_WCHB1.81.s\_\_uncultured\_actinobacterium |
| 1129 | OTU1129 | d\_\_Archaea.p\_\_Thermoplasmatota.c\_\_Thermoplasmata.o\_\_Marine\_Benthic\_Group\_D\_and\_DHVEG.1.f\_\_Marine\_Benthic\_Group\_D\_and\_DHVEG.1.g\_\_Marine\_Benthic\_Group\_D\_and\_DHVEG.1.s\_\_archaeon\_enrichment |
| 1142 | OTU1142 | d\_\_Bacteria.p\_\_Campilobacterota.c\_\_Campylobacteria.o\_\_Campylobacterales.f\_\_Sulfurimonadaceae.**.** |
| 1150 | OTU1150 | d\_\_Bacteria.p\_\_Proteobacteria.c\_\_Gammaproteobacteria.o\_\_Chromatiales.f\_\_Chromatiaceae.g\_\_Candidatus\_Thiobios.s\_\_uncultured\_gamma |
| 1160 | OTU1160 | d\_\_Bacteria.p\_\_Proteobacteria.c\_\_Alphaproteobacteria.o\_\_Caulobacterales.f\_\_Parvularculaceae.g**Parvularcula.** |
| 1162 | OTU1162 | d\_\_Bacteria.p\_\_Proteobacteria.c\_\_Alphaproteobacteria.o\_\_Rhodobacterales.f\_\_Rhodobacteraceae.g**Roseobacter.** |
| 1173 | OTU1173 | d\_\_Bacteria.p\_\_Actinobacteriota.c\_\_Acidimicrobiia.o\_\_Actinomarinales.f\_\_uncultured.g\_\_uncultured.s\_\_bacterium\_WH6.7 |
| 1178 | OTU1178 | d\_\_Bacteria.p\_\_Firmicutes.c\_\_Bacilli.o\_\_Izemoplasmatales.f\_\_Izemoplasmataceae.g\_\_Izimaplasma.s\_\_Candidatus\_Izimaplasma |
| 1189 | OTU1189 | d\_\_Bacteria.p\_\_Desulfobacterota.c\_\_Desulfobacteria.o\_\_Desulfobacterales.f\_\_Desulfobacteraceae.g\_\_Desulfospira.s\_\_uncultured\_bacterium |
| 1194 | OTU1194 | d\_\_Bacteria.p\_\_Proteobacteria.c\_\_Gammaproteobacteria.o\_\_Oceanospirillales.f\_\_Saccharospirillaceae.g**Reinekea.** |
| 1196 | OTU1196 | d\_\_Bacteria.p\_\_CK.2C2.2.c\_\_CK.2C2.2.o\_\_CK.2C2.2.f\_\_CK.2C2.2.g\_\_CK.2C2.2.s\_\_uncultured\_organism |
| 1198 | OTU1198 | d\_\_Bacteria.p\_\_Bacteroidota.c\_\_Bacteroidia.o\_\_Flavobacteriales.f\_\_Cryomorphaceae.g\_\_Vicingus.s\_\_uncultured\_sediment |
| 1205 | OTU1205 | d\_\_Bacteria.p\_\_Chloroflexi.c\_\_Chloroflexia.o**Thermomicrobiales.**.**.** |
| 1226 | OTU1226 | d\_\_Bacteria.p\_\_Proteobacteria.c\_\_Alphaproteobacteria.o\_\_Rhizobiales.f\_\_Hyphomicrobiaceae.g**Filomicrobium.** |
| 1228 | OTU1228 | d\_\_Bacteria.p\_\_Planctomycetota.c\_\_Planctomycetes.o\_\_Pirellulales.f\_\_Pirellulaceae.g\_\_Rhodopirellula.s\_\_uncultured\_hydrocarbon |
| 1235 | OTU1235 | d\_\_Bacteria.p\_\_Proteobacteria.c\_\_Alphaproteobacteria.o\_\_Micavibrionales.f\_\_uncultured.g\_\_uncultured.s\_\_uncultured\_organism |
| 1238 | OTU1238 | d\_\_Bacteria.p\_\_Acidobacteriota.c\_\_Thermoanaerobaculia.o\_\_Thermoanaerobaculales.f\_\_Thermoanaerobaculaceae.g\_\_Subgroup\_23.s\_\_uncultured\_prokaryote |
| 1240 | OTU1240 | d\_\_Bacteria.p\_\_Myxococcota.c\_\_Polyangia.o\_\_VHS.B4.70.f\_\_VHS.B4.70.g\_\_VHS.B4.70.s\_\_uncultured\_delta |
| 1248 | OTU1248 | d\_\_Bacteria.p\_\_Planctomycetota.c\_\_Phycisphaerae.o\_\_mle1.8.f\_\_mle1.8.g\_\_mle1.8.s\_\_uncultured\_organism |
| 1249 | OTU1249 | d\_\_Bacteria.p\_\_Myxococcota.c\_\_Polyangia.o\_\_Polyangiales.f\_\_Sandaracinaceae.g**Sandaracinus.** |
| 1250 | OTU1250 | d\_\_Bacteria.p\_\_Proteobacteria.c\_\_Gammaproteobacteria.o\_\_Nitrosococcales.f\_\_Nitrosococcaceae.g**CI75cm.2.12.** |
| 1284 | OTU1284 | d\_\_Bacteria.p\_\_Proteobacteria.c\_\_Gammaproteobacteria.o\_\_Steroidobacterales.f\_\_Woeseiaceae.g\_\_JTB255\_marine\_benthic\_group.s\_\_uncultured\_organism |
| 1285 | OTU1285 | d\_\_Bacteria.p\_\_Fibrobacterota.c\_\_Fibrobacteria.o\_\_Fibrobacterales.f\_\_TG3.g**TG3.** |
| 1287 | OTU1287 | d\_\_Bacteria.p\_\_Proteobacteria.c\_\_Gammaproteobacteria.o\_\_OM182\_clade.f\_\_OM182\_clade.g\_\_OM182\_clade.s\_\_unidentified\_marine |
| 1289 | OTU1289 | d\_\_Bacteria.p\_\_Verrucomicrobiota.c\_\_Lentisphaeria.o\_\_SS1.B.02.17.f\_\_SS1.B.02.17.g**SS1.B.02.17.** |
| 1298 | OTU1298 | d\_\_Bacteria.p\_\_Bacteroidota.c\_\_Bacteroidia.o\_\_Chitinophagales.f\_\_Saprospiraceae.g\_\_uncultured.s\_\_uncultured\_Bacteroidetes |
| 1299 | OTU1299 | d\_\_Bacteria.p\_\_Verrucomicrobiota.c\_\_Kiritimatiellae.o\_\_Kiritimatiellales.f\_\_Kiritimatiellaceae.g\_\_R76.B128.s\_\_Kiritimatiella\_sp. |
| 1301 | OTU1301 | d\_\_Bacteria.p\_\_Fusobacteriota.c\_\_Fusobacteriia.o\_\_Fusobacteriales.f\_\_Fusobacteriaceae.g**Fusobacterium.** |
| 1302 | OTU1302 | d\_\_Archaea.p\_\_Asgardarchaeota.c\_\_Lokiarchaeia.o\_\_Lokiarchaeia.f\_\_Lokiarchaeia.g\_\_Lokiarchaeia.s\_\_uncultured\_crenarchaeote |
| 1303 | OTU1303 | d\_\_Bacteria.p\_\_Desulfobacterota.c\_\_Desulfuromonadia.o\_\_uncultured.f\_\_uncultured.g\_\_uncultured.s\_\_uncultured\_bacterium |
| 1308 | OTU1308 | d\_\_Bacteria.p\_\_Planctomycetota.c\_\_Phycisphaerae.o\_\_mle1.8.f\_\_mle1.8.g**mle1.8.** |
| 1310 | OTU1310 | d\_\_Bacteria.p\_\_Spirochaetota.c\_\_MVP.15.o\_\_MVP.15.f\_\_MVP.15.g\_\_MVP.15.s\_\_uncultured\_bacterium |
| 1312 | OTU1312 | d\_\_Bacteria.p\_\_Campilobacterota.c\_\_Campylobacteria.o\_\_Campylobacterales.f\_\_Sulfurospirillaceae.g**Sulfurospirillum.** |
| 1314 | OTU1314 | d\_\_Bacteria.p\_\_Proteobacteria.c\_\_Gammaproteobacteria.o\_\_Nitrosococcales.f\_\_Nitrosococcaceae.g\_\_SZB85.s\_\_uncultured\_bacterium |
| 1318 | OTU1318 | d\_\_Archaea.p\_\_Thermoplasmatota.c\_\_Thermoplasmata.o\_\_Methanomassiliicoccales.f\_\_uncultured.g\_\_uncultured.s\_\_uncultured\_euryarchaeote |
| 1321 | OTU1321 | d\_\_Bacteria.p\_\_Cyanobacteria.c\_\_Cyanobacteriia.o\_\_Cyanobacteriales.f\_\_Xenococcaceae.g\_\_Chroococcidiopsis\_PCC.6712.s\_\_uncultured\_cyanobacterium |
| 1325 | OTU1325 | d\_\_Bacteria.p\_\_Proteobacteria.c\_\_Alphaproteobacteria.o\_\_uncultured.f\_\_uncultured.g**uncultured.** |
| 1329 | OTU1329 | d\_\_Bacteria.p\_\_Desulfobacterota.c\_\_Desulfobacteria.o\_\_Desulfobacterales.f\_\_Desulfococcaceae.g**Desulfonema.** |
| 1330 | OTU1330 | d\_\_Bacteria.p\_\_Proteobacteria.c\_\_Gammaproteobacteria.o\_\_Thiotrichales.f\_\_Thiotrichaceae.**.** |
| 1338 | OTU1338 | d\_\_Bacteria.p\_\_Myxococcota.c\_\_Polyangia.o\_\_Blfdi19.f\_\_Blfdi19.g\_\_Blfdi19.s\_\_uncultured\_bacterium |
| 1343 | OTU1343 | d\_\_Bacteria.p\_\_Acidobacteriota.c\_\_Thermoanaerobaculia.o\_\_Thermoanaerobaculales.f\_\_Thermoanaerobaculaceae.g\_\_B276.D12.s\_\_uncultured\_bacterium |
| 1347 | OTU1347 | d\_\_Bacteria.p\_\_Fibrobacterota.c\_\_Chitinivibrionia.o\_\_uncultured.f\_\_uncultured.g**uncultured.** |
| 1350 | OTU1350 | d\_\_Bacteria.p\_\_Bacteroidota.c\_\_Bacteroidia.o\_\_Flavobacteriales.f\_\_Flavobacteriaceae.g**Polaribacter.** |
| 1351 | OTU1351 | d\_\_Bacteria.p\_\_Planctomycetota.c\_\_Phycisphaerae.o\_\_Phycisphaerales.f\_\_AKAU3564\_sediment\_group.g\_\_AKAU3564\_sediment\_group.s\_\_uncultured\_bacterium |
| 1352 | OTU1352 | d\_\_Bacteria.p\_\_Proteobacteria.c\_\_Gammaproteobacteria.o\_\_D90.f\_\_D90.g\_\_D90.s\_\_uncultured\_gamma |
| 1353 | OTU1353 | d\_\_Bacteria.p\_\_Planctomycetota.c\_\_Phycisphaerae.o\_\_Amsterdam.1B.07.f\_\_Amsterdam.1B.07.g\_\_Amsterdam.1B.07.s\_\_uncultured\_bacterium |
| 1359 | OTU1359 | d\_\_Bacteria.p\_\_Patescibacteria.c\_\_Parcubacteria.o\_\_Candidatus\_Kaiserbacteria.f\_\_Candidatus\_Kaiserbacteria.g\_\_Candidatus\_Kaiserbacteria.s\_\_bacterium\_SH4.10 |
| 1361 | OTU1361 | d\_\_Bacteria.p\_\_Chloroflexi.c\_\_Anaerolineae.o\_\_Anaerolineales.f\_\_Anaerolineaceae.g**Pelolinea.** |
| 1362 | OTU1362 | d\_\_Bacteria.p\_\_Gemmatimonadota.c\_\_BD2.11\_terrestrial\_group.o\_\_BD2.11\_terrestrial\_group.f\_\_BD2.11\_terrestrial\_group.g\_\_BD2.11\_terrestrial\_group.s\_\_unidentified\_bacterium |
| 1363 | OTU1363 | d\_\_Bacteria.p\_\_Sumerlaeota.c\_\_Sumerlaeia.o\_\_uncultured.f\_\_uncultured.g\_\_uncultured.s\_\_uncultured\_organism |
| 1369 | OTU1369 | d\_\_Bacteria.p\_\_Planctomycetota.c\_\_Phycisphaerae.o\_\_Phycisphaerales.f\_\_Phycisphaeraceae.g\_\_uncultured.s\_\_uncultured\_bacterium |
| 1370 | OTU1370 | d\_\_Bacteria.p\_\_Latescibacterota.c\_\_Latescibacterota.o\_\_Latescibacterota.f\_\_Latescibacterota.g\_\_Latescibacterota.s\_\_uncultured\_Acidobacterium |
| 1374 | OTU1374 | d\_\_Bacteria.p\_\_Proteobacteria.c\_\_Alphaproteobacteria.o\_\_Defluviicoccales.f\_\_Defluviicoccaceae.g\_\_Defluviicoccus.s\_\_uncultured\_Rhodospirillaceae |

The separation between groups is 3.12.

We update the plot with the separations:

```
intersección=c(dim(Significant.capat)[1],Sep(HC))
Resultados=rbind(Resultados,intersección)
row.names(Resultados)=c(
"LR.max",
"LR.max.local",
"Aldex",
"glmnet",
"glmnet.ext",
"intersection")
colnames(Resultados)=c("Size","Separation")
```

```
par(opar)
fit = smooth.spline(F.SS[!is.na(F.SS[,3]),1],F.SS[!is.na(F.SS[,3]),3],cv=TRUE)
fit.low = smooth.spline(F.SS[!is.na(F.SS[,3]),1],F.SS[!is.na(F.SS[,3]),4],cv=TRUE)
fit.up = smooth.spline(F.SS[!is.na(F.SS[,3]),1],F.SS[!is.na(F.SS[,3]),5],cv=TRUE)

plot(F.SS[,c(1,3)],pch=20,cex=0.25,col="darkgrey",xlab="n",ylab="Separation",main="Average separation for perfect classifiers",ylim=c(0,2.25),xlim=c(0,250),xaxp=c(0,250,10),yaxp=c(0,2.5,10))
lines(fit,col="black",lwd=1)
lines(fit.low,col="red",lwd=1)
lines(fit.up,col="red",lwd=1)
points(Resultados,col="blue",type="h")
points(Resultados,col="blue",pch=20)
text(Resultados[,1],Resultados[,2]+0.02,col="blue",labels=row.names(Resultados),cex=0.75)
abline(h=2.47,col="green")
text(20,2.6,col="green",labels="Global",cex=0.75)
```

```
Resultados %>%
  kbl() %>%
  kable_styling()
```

|  | Size | Separation |
| --- | --- | --- |
| LR.max | 549 | 2.829712 |
| LR.max.local | 46 | 4.655234 |
| Aldex | 121 | 3.429094 |
| glmnet | 350 | 2.776603 |
| glmnet.ext | 104 | 4.421816 |
| intersection | 312 | 3.116542 |

### 5.3 CAU T2C vs CAU T2HW

```
tax_otu=as.data.frame(read_excel("TablaOTUs.xlsx"))[1:12,]

Samples=tax_otu$ID 
taxa=colnames(tax_otu[,3:dim(tax_otu)[2]])
OTUs=paste("OTU",1:(dim(tax_otu)[2]-2),sep="")

DF.0=tax_otu[,-c(1,2)]
row.names(DF.0)=Samples
colnames(DF.0)=OTUs

taxa.original=taxa
DF.0.original=DF.0
n.OTUs.original=dim(DF.0)[2]

Miseria=as.vector(which(colSums(DF.0)<=2))
DF.0=DF.0[,-Miseria]
taxa=taxa[-Miseria]
OTUs=OTUs[-Miseria]

hit=function(x){min(c(x,1))}
DF.0.hits=as.matrix(DF.0)
DF.0.hits[]=vapply(DF.0.hits, hit, numeric(1))

Unics=data.frame(
Sample=rep(NA,length(which(colSums(DF.0.hits)==1))), OTUs=names(colSums(DF.0)[which(colSums(DF.0.hits)==1)]), Reads=colSums(DF.0)[which(colSums(DF.0.hits)==1)],
            Proportions=rep(0,length(which(colSums(DF.0.hits)==1)))  
)
for (i in 1:length(Unics$OTUs)){
ii=which(DF.0.hits[,Unics$OTUs[i]]==1)  
Unics$Proportions[i]=DF.0[ii,Unics$OTUs[i]]/sum(DF.0[ii,])
Unics$Sample[i]=Samples[ii]}
Unics.Freqs=Unics[Unics$Proportions>=0.05/100,]
row.names(Unics.Freqs)=NULL


DF.0=DF.0[,-which(colSums(DF.0.hits)==1)]
taxa=taxa[-which(colSums(DF.0.hits)==1)]
OTUs=OTUs[-which(colSums(DF.0.hits)==1)]

row.names(DF.0)=Samples

DF=zCompositions::cmultRepl(DF.0,method="GBM",output="p-counts",suppress.print=TRUE,z.warning=0.99)
```

```
DF=DF[7:12,]
DF.0=DF.0[7:12,]
DF.0.original=DF.0.original[7:12 ,]

Type=as.factor(c(rep("CAU_T2C", 3),rep("CAU_T2HW", 3)))
colors=c("purple","red")[Type]
```

After removing OTUs with 2 or fewer reads across the CAU samples and those appearing in only one of these samples, 1682 taxa remain.

The OTUs removed at this step that represent more than 0.05% of the sample in which they appear are:

```
Unics.Freqs[rev(order(Unics.Freqs$Proportions)),] %>%
  kbl() %>%
  kable_styling()
```

|  | Sample | OTUs | Reads | Proportions |
| --- | --- | --- | --- | --- |
| 2 | CAU\_T0\_2 | OTU966 | 55 | 0.0012193 |
| 1 | CAU\_T2HW\_1 | OTU727 | 55 | 0.0008798 |

```
rm(tax_otu)
rm(DF.0.hits)
rm(Miseria)
rm(Unics.Freqs)
```

#### 5.3.1 How different are the samples?

```
HC=ClusterHC(DF,Grups=Type,colores=colors)
```

```
base=Sep(HC)
```

The two sample types are NOT well separated.

We examine the proportion of OTU subsets of reasonable size that already classify them correctly.

```
# Fix a random seed for the simulation
> initial_seed=as.integer(Sys.time())
> print (initial_seed)
# [1] 1715851715853234
initial_seed %% 10000
# [1] 3234
```

```
set.seed(3234)
Experimento=function(i)
{
  ii=sample(dim(DF)[2],i,rep=FALSE)
  DFtemp=DF[,ii]
  CC=ClusterHC(DFtemp,dendrograma=FALSE,barplot=FALSE, Grups=Type,colores=colors)
  c(Encerts(CC$tabla,table(Type)),Sep(CC))
}
n=500
F.SS=c()
for (i in 10:1000)
  {
  print(i)
  EE=replicate(n,Experimento(i))
  EE.2=EE[2,which(EE[1,]==0)]
  if(length(which(EE[1,]==0))>0){
XX=replicate(1000,mean(sample(EE.2,n,replace=TRUE)))
  F.SS=rbind(F.SS, c(i,
                     length(EE.2)/250,
                      mean(EE.2),
                      quantile(XX,0.025),
                      quantile(XX,0.975)
  ))}
  else {
    F.SS=rbind(F.SS, c(i,
                     0,
                      NA,
                      NA,
                      NA))
  }
  }
colnames(F.SS)=c("n","Proportion","Avg. sep.","Q_0.025","Q_0.975")
saveRDS(F.SS, file="Fraccio.Separadors.CAU.T2CvsT2HW.RData")
```

```
F.SS=readRDS("Fraccio.Separadors.CAU.T2CvsT2HW.RData")
```

The first plot shows, for each \(n\) up to 600, the proportion of samples of size \(n\) that perfectly classify CAU\_T2C and CAU\_T2HW (black curve), along with the 95% Clopper–Pearson confidence interval for that proportion (red curves). We used *smoothing splines* to smooth the curves.

```
F.SS_prop.Q_0.025=epitools::binom.exact(F.SS[,2]*250,250)$lower
F.SS_prop.Q_0.975=epitools::binom.exact(F.SS[,2]*250,250)$upper
fit = smooth.spline(F.SS[,1],F.SS[,2],cv=TRUE)
fit.low = smooth.spline(F.SS[,1],F.SS_prop.Q_0.025,cv=TRUE)
fit.up = smooth.spline(F.SS[,1],F.SS_prop.Q_0.975,cv=TRUE)


plot(F.SS[,1:2],pch=20,cex=0.25,col="darkgrey",xlab="n",ylab="Proportion",main="Proportion of perfect classifiers",ylim=c(0,0.1),xlim=c(0,600),xaxp=c(0,600,12),yaxp=c(0,0.1,10))
lines(fit,col="black",lwd=1)
lines(fit.low,col="red",lwd=1)
lines(fit.up,col="red",lwd=1)
```

The second plot shows, for each \(n\) up to 600, the estimated mean separation obtained with a sample of size \(n\) that perfectly classifies CAU\_T2C and CAU\_T2HW (black curve), along with the 95% confidence interval of this mean separation obtained via bootstrap (red curve). Note that from \(n=350\) onward, NO subset is found that separates the two groups.

```
fit = smooth.spline(F.SS[!is.na(F.SS[,3]),1],F.SS[!is.na(F.SS[,3]),3],cv=TRUE)
fit.low = smooth.spline(F.SS[!is.na(F.SS[,3]),1],F.SS[!is.na(F.SS[,3]),4],cv=TRUE)
fit.up = smooth.spline(F.SS[!is.na(F.SS[,3]),1],F.SS[!is.na(F.SS[,3]),5],cv=TRUE)

plot(F.SS[,c(1,3)],pch=20,cex=0.25,col="darkgrey",xlab="n",ylab="Separation",main="Average separation for perfect classifiers",xlim=c(0,600),xaxp=c(0,600,12),yaxp=c(0,1.5,15))
lines(fit,col="black",lwd=1)
lines(fit.low,col="red",lwd=1)
lines(fit.up,col="red",lwd=1)
```

`

#### 5.3.2 Taxa with maximum relevant log-ratios

We apply the LR approach exactly as described in the first comparison: OTUs are ranked by their log-ratio relevance, and we retain the subset maximizing the average absolute within-subset log-ratio association.

```
LRS=explore_logratios(DF,Type)
saveRDS(LRS, file="LRS_CAU_2C2HW.RData")
```

```
LRS=readRDS("LRS_CAU_2C2HW.RData")
```

```
assoc=rep(0,dim(DF)[2]-4)
for (m in 5:dim(DF)[2]){
    assoc[m-4]=sum(LRS$`association log-ratio with y`[1:m,1:m])/m^2
}
saveRDS(assoc, file="assoc_CAU_2C2HW.RData")
```

```
assoc=readRDS("assoc_CAU_2C2HW.RData")
plot(5:dim(DF)[2],assoc,type="b",xlab="m",ylab="Average goodness of the m most relevant." ,pch=20,cex=0.5)
```

```
m=4+which.max(assoc)
impLR=sort(as.numeric(LRS$`order of importance`[1:m]))

HC=ClusterHC(DF[,impLR],barplot=FALSE,Grups=Type,colores=colors)
```

```
LR.max=c(length(impLR),Sep(HC))
```

The maximum is attained at the set of 95 most logratio-relevant taxa.  
They perfectly separate the two sample types. The separation between groups is 1.98.

```
Significant=data.frame(
  OTUs=paste("OTU",1:n.OTUs.original,sep=""),
  taxa=taxa.original,
  Log_ratio.max=Indicador(impLR))
```

#### 5.3.3 Taxa relevant for the bacterial signature (according to `coda_glmnet`)

As described above, `coda_glmnet` identifies a microbial signature by fitting a penalized log-contrast model and retaining OTUs with non-zero coefficients.

```
coda.glmnet=coda_glmnet(DF,Type,showPlots = FALSE)
```

```
## [1] "No variables are selected. The prediction and the signature plots are not displayed."
```

```
coda.glmnet$`signature plot`
```

```
## NULL
```

In this 3 vs. 3 comparison, `coda_glmnet` may fail to identify non-zero coefficients because the penalized model has limited power with such a small sample size. To explore whether a more stable microbial signature can be recovered, we augment the analysis with ALDEx-derived random replicates for each original sample. These replicated compositional instances are not treated as new biological observations, but as a sensitivity analysis intended to stabilize the log-ratio structure before refitting the penalized model. We evaluate this strategy using 3, 5, and 10 replicates per original sample.

```
> initial_seed=as.integer(Sys.time())
> print (initial_seed)
# [1] 1711435693
initial_seed %% 10000
# [1] 5693
```

```
# 3 replicates
set.seed(5693)
M=3
repls=aldexCesc.clr(t(DF), conds=Type, mc.samples=M)@analysisData
```

```
## [1] "operating in serial mode"
```

```
repls=t(cbind(repls$CAU_T2C_1,repls$CAU_T2C_2,repls$CAU_T2C_3,repls$CAU_T2HW_1,repls$CAU_T2HW_2,repls$CAU_T2HW_3))
attr(repls,"dimnames")[[2]]=NULL
colnames(repls)=colnames(DF)
row.names(repls)=c(paste("CAU_T2C_1",1:M,sep="_"),
  paste("CAU_T2C_2",1:M,sep="_"),
  paste("CAU_T2C_3",1:M,sep="_"),
  paste("CAU_T2HW_1",1:M,sep="_"),
  paste("CAU_T2HW_2",1:M,sep="_"),
  paste("CAU_T2HW_3",1:M,sep="_"))
mostres=rbind(repls,easyCODA::CLR(DF)$LR)
rm(repls)
Type_ext=as.factor(c(rep("CAU_T2C", 3*M),rep("CAU_T2HW", 3*M),rep("CAU_T2C", 3),rep("CAU_T2HW", 3)))
colors_ext=c("purple","red")[Type_ext]
```

```
LogRat_mostres=logratios_matrix_clr(mostres)
saveRDS(LogRat_mostres, file="LogRat_mostres_CAU_2C2HW_3copies.RData")
```

```
LogRat_mostres=readRDS("LogRat_mostres_CAU_2C2HW_3copies.RData")
LogRat_mostres=list(LogRat_mostres[[1]],LogRat_mostres[[2]])

coda.glmnet.3=coda_glmnet_lr_bin(LogRat_mostres,Type_ext,taxa,showPlots = FALSE)
```

```
impGLMNET_ext.3=coda.glmnet.3$taxa.num
HC=ClusterHC(DF[,impGLMNET_ext.3],dendrograma=TRUE,barplot=FALSE,Grups=Type,colores=colors)
```

```
glmnet.ext.3=c(length(impGLMNET_ext.3),Sep(HC))
```

With 3 replicates, 42 significant taxa are found, perfectly separating the two groups, with a separation of 1.49.

```
# 5 replicates
set.seed(5693)
M=5
repls=aldexCesc.clr(t(DF), conds=Type, mc.samples=M)@analysisData
```

```
## [1] "operating in serial mode"
```

```
repls=t(cbind(repls$CAU_T2C_1,repls$CAU_T2C_2,repls$CAU_T2C_3,repls$CAU_T2HW_1,repls$CAU_T2HW_2,repls$CAU_T2HW_3))
attr(repls,"dimnames")[[2]]=NULL
colnames(repls)=colnames(DF)
row.names(repls)=c(paste("CAU_T2C_1",1:M,sep="_"),
  paste("CAU_T2C_2",1:M,sep="_"),
  paste("CAU_T2C_3",1:M,sep="_"),
  paste("CAU_T2HW_1",1:M,sep="_"),
  paste("CAU_T2HW_2",1:M,sep="_"),
  paste("CAU_T2HW_3",1:M,sep="_"))
mostres=rbind(repls,easyCODA::CLR(DF)$LR)
rm(repls)
Type_ext=as.factor(c(rep("CAU_T2C", 3*M),rep("CAU_T2HW", 3*M),rep("CAU_T2C", 3),rep("CAU_T2HW", 3)))
colors_ext=c("purple","red")[Type_ext]
```

```
LogRat_mostres=logratios_matrix_clr(mostres)
saveRDS(LogRat_mostres, file="LogRat_mostres_CAU_2C2HW_5copies.RData")
```

```
LogRat_mostres=readRDS("LogRat_mostres_CAU_2C2HW_5copies.RData")
LogRat_mostres=list(LogRat_mostres[[1]],LogRat_mostres[[2]])

coda.glmnet.5=coda_glmnet_lr_bin(LogRat_mostres,Type_ext,taxa,showPlots = FALSE)
```

```
impGLMNET_ext.5=coda.glmnet.5$taxa.num
HC=ClusterHC(DF[,impGLMNET_ext.5],dendrograma=TRUE,barplot=FALSE,Grups=Type,colores=colors)
```

```
glmnet.ext.5=c(length(impGLMNET_ext.5),Sep(HC))
```

With 5 replicates, 53 significant taxa are found, perfectly separating the two groups, with a separation of 1.2.

```
Significant=cbind(Significant,
glmnet_sign.ext.5.T2C=Indicador(coda.glmnet.5$taxa.num[coda.glmnet.5$`log-contrast coefficients`>0]),
glmnet_sign.ext.5.T2HW=Indicador(coda.glmnet.5$taxa.num[coda.glmnet.5$`log-contrast coefficients`<0]))
```

```
set.seed(5693)
M=10
repls=aldexCesc.clr(t(DF), conds=Type, mc.samples=M)@analysisData
```

```
## [1] "operating in serial mode"
```

```
repls=t(cbind(repls$CAU_T2C_1,repls$CAU_T2C_2,repls$CAU_T2C_3,repls$CAU_T2HW_1,repls$CAU_T2HW_2,repls$CAU_T2HW_3))
attr(repls,"dimnames")[[2]]=NULL
colnames(repls)=colnames(DF)
row.names(repls)=c(paste("CAU_T2C_1",1:M,sep="_"),
  paste("CAU_T2C_2",1:M,sep="_"),
  paste("CAU_T2C_3",1:M,sep="_"),
  paste("CAU_T2HW_1",1:M,sep="_"),
  paste("CAU_T2HW_2",1:M,sep="_"),
  paste("CAU_T2HW_3",1:M,sep="_"))
mostres=rbind(repls,easyCODA::CLR(DF)$LR)
rm(repls)
Type_ext=as.factor(c(rep("CAU_T2C", 3*M),rep("CAU_T2HW", 3*M),rep("CAU_T2C", 3),rep("CAU_T2HW", 3)))
colors_ext=c("purple","red")[Type_ext]
```

```
LogRat_mostres=logratios_matrix_clr(mostres)
saveRDS(LogRat_mostres, file="LogRat_mostres_CAU_2C2HW_10copies.RData")
```

```
LogRat_mostres=readRDS("LogRat_mostres_CAU_2C2HW_10copies.RData")
LogRat_mostres=list(LogRat_mostres[[1]],LogRat_mostres[[2]])

coda.glmnet.10=coda_glmnet_lr_bin(LogRat_mostres,Type_ext,taxa,showPlots = FALSE)
```

```
impGLMNET_ext.10=coda.glmnet.10$taxa.num
HC=ClusterHC(DF[,impGLMNET_ext.10],dendrograma=TRUE,barplot=FALSE,Grups=Type,colores=colors)
```

```
glmnet.ext.10=c(length(impGLMNET_ext.10),Sep(HC))
```

With 10 replicates, 59 significant taxa are found, perfectly separating the two groups, with a separation of 1.12.

```
Significant=cbind(Significant,
glmnet_sign.ext.10.T2C=Indicador(coda.glmnet.10$taxa.num[coda.glmnet.10$`log-contrast coefficients`>0]),
glmnet_sign.ext.10.T2HW=Indicador(coda.glmnet.10$taxa.num[coda.glmnet.10$`log-contrast coefficients`<0]))
```

#### 5.3.4 OTUs significant according to ALDeX

```
> initial_seed=as.integer(Sys.time())
> print (initial_seed)
# [1] 1711461735
initial_seed %% 10000
# [1] 1735
```

```
set.seed(1735)
repl_kw=aldexCesc.clr(t(DF), conds=Type, mc.samples=1000, verbose=FALSE)
aldex_kw=aldex.kw(repl_kw, verbose=FALSE)
#
saveRDS(repl_kw, file="repl_kw_CAU_2C2HW.RData")
saveRDS(aldex_kw, file="aldex_kw_CAU_2C2HW.RData")
```

```
p.valores_kw=readRDS("aldex_kw_CAU_2C2HW.RData")
```

```
min(p.valores_kw[,1])
```

```
## [1] 0.04999719
```

```
min(p.valores_kw[,2])
```

```
## [1] 0.3641574
```

```
min(p.valores_kw[,3])
```

```
## [1] 0.002010094
```

```
min(p.valores_kw[,4])
```

```
## [1] 0.07355742
```

```
length(p.valores_kw[p.valores_kw[,3]<0.01,2])
```

```
## [1] 11
```

```
length(p.valores_kw[p.valores_kw[,3]<0.05,2])
```

```
## [1] 68
```

There are no taxa with even an unadjusted p-value < 0.05.

#### 5.3.5 Simper

```
DF.prop=DF.0.original/rowSums(DF.0.original)
SMP.prop=simper(DF.prop,group=Type)
indexos_SMP.07=SMP.prop$CAU_T2C_CAU_T2HW$ord[which(SMP.prop$CAU_T2C_CAU_T2HW$cusum<=0.7)]
indexos_SMP.05=SMP.prop$CAU_T2C_CAU_T2HW$ord[which(SMP.prop$CAU_T2C_CAU_T2HW$cusum<=0.5)]

OTUs_SMP.07=sort(attr(SMP.prop$CAU_T2C_CAU_T2HW$cusum[which(SMP.prop$CAU_T2C_CAU_T2HW$cusum<=0.7)],"names"))
OTUs_SMP.good.07=setdiff(OTUs_SMP.07,Unics$OTUs)
```

`simper` identifies 170 significant taxa, but they do not separate the samples well.

```
HC=ClusterHC(DF[,OTUs_SMP.good.07],dendrograma=TRUE,barplot=FALSE,Grups=Type,colores=colors)
```

```
Significant=cbind(Significant,
t(DF.prop))
```

#### 5.3.6 Summary

```
write.csv2(Significant,"OTU_Significativos_CAU_T2.csv",row.names=FALSE)
```

```
Resultados=rbind(
LR.max,
glmnet.ext.3,
glmnet.ext.5,
glmnet.ext.10

)
row.names(Resultados)=c(
"LR",
"glmnet.ext.3",
"glmnet.ext.5",
"glmnet.ext.10")
colnames(Resultados)=c("Size","Separation")
```

```
fit = smooth.spline(F.SS[!is.na(F.SS[,3]),1],F.SS[!is.na(F.SS[,3]),3],cv=TRUE)
fit.low = smooth.spline(F.SS[!is.na(F.SS[,3]),1],F.SS[!is.na(F.SS[,3]),4],cv=TRUE)
fit.up = smooth.spline(F.SS[!is.na(F.SS[,3]),1],F.SS[!is.na(F.SS[,3]),5],cv=TRUE)

plot(F.SS[,c(1,3)],pch=20,cex=0.25,col="darkgrey",xlab="n",ylab="Separation",main="Average separation for perfect classifiers",ylim=c(0,2.25),xlim=c(0,250),xaxp=c(0,250,10),yaxp=c(0,2.5,10))
lines(fit,col="black",lwd=1)
lines(fit.low,col="red",lwd=1)
lines(fit.up,col="red",lwd=1)

points(Resultados,col="blue",type="h")
points(Resultados,col="blue",pch=20)
text(Resultados[,1],Resultados[,2]+0.02,col="blue",labels=row.names(Resultados),cex=0.75)
```

Finally, we intersect the OTUs identified as significant according to LR and `coda_glmnet` with 3 replicates

```
Significant.capat=Significant[,c(1,2,3,4,5)]
Significant.capat=cbind(Significant.capat,Cuánto=rowSums(Significant.capat[,3:dim(Significant.capat)[2]]))
Significant.capat=Significant.capat[Significant.capat$Cuánto==2,]
```

```
DF.Imp=DF[,Significant.capat[,1]]
HC=ClusterHC(DF.Imp,dendrograma=TRUE,barplot=FALSE,Grups=Type,colores=colors)
```

There are 10 taxa. They are:

```
Significant.capat[,1:2]%>%
  kbl() %>%
  kable_styling()
```

|  | OTUs | taxa |
| --- | --- | --- |
| OTU38 | OTU38 | Unassigned;**;**;**;**;**;** |
| OTU108 | OTU108 | d\_\_Bacteria;p\_\_Spirochaetota;c\_\_Spirochaetia;o\_\_Spirochaetales;f\_\_Spirochaetaceae;g**GWE2-31-10;** |
| OTU199 | OTU199 | d\_\_Bacteria;p\_\_Proteobacteria;c\_\_Gammaproteobacteria;o\_\_Oceanospirillales;f\_\_Halomonadaceae;g**Cobetia;** |
| OTU208 | OTU208 | d\_\_Bacteria;p\_\_Bacteroidota;c\_\_Bacteroidia;o\_\_Flavobacteriales;f\_\_Flavobacteriaceae;g**Lutibacter;** |
| OTU337 | OTU337 | d\_\_Bacteria;p\_\_Chloroflexi;c\_\_Anaerolineae;o\_\_SJA-15;f\_\_SJA-15;g\_\_SJA-15;s\_\_uncultured\_Chloroflexi |
| OTU365 | OTU365 | d\_\_Bacteria;p\_\_Firmicutes;c\_\_Clostridia;o**Lachnospirales;**;**;** |
| OTU383 | OTU383 | d\_\_Bacteria;p\_\_Proteobacteria;c\_\_Gammaproteobacteria;o\_\_Alteromonadales;f\_\_Alteromonadaceae;**;** |
| OTU685 | OTU685 | d\_\_Bacteria;p\_\_PAUC34f;c\_\_PAUC34f;o\_\_PAUC34f;f\_\_PAUC34f;g\_\_PAUC34f;s\_\_uncultured\_bacterium |
| OTU708 | OTU708 | d\_\_Bacteria;p\_\_Patescibacteria;c\_\_Gracilibacteria;o\_\_Gracilibacteria;f\_\_Gracilibacteria;g\_\_Gracilibacteria;s\_\_uncultured\_organism |
| OTU1120 | OTU1120 | d\_\_Bacteria;p\_\_Calditrichota;c\_\_Calditrichia;o\_\_Calditrichales;f\_\_Calditrichaceae;g**Caldithrix;** |

The separation between groups is 1.96.

We update the plot with the separations:

```
intersección=c(dim(Significant.capat)[1],Sep(HC))
Resultados=rbind(Resultados,intersección)
row.names(Resultados)=c(
"LR",
"glmnet.ext.3",
"glmnet.ext.5",
"glmnet.ext.10",
"intersection")
colnames(Resultados)=c("Size","Separation")
```

```
par(opar)
fit = smooth.spline(F.SS[!is.na(F.SS[,3]),1],F.SS[!is.na(F.SS[,3]),3],cv=TRUE)
fit.low = smooth.spline(F.SS[!is.na(F.SS[,3]),1],F.SS[!is.na(F.SS[,3]),4],cv=TRUE)
fit.up = smooth.spline(F.SS[!is.na(F.SS[,3]),1],F.SS[!is.na(F.SS[,3]),5],cv=TRUE)

plot(F.SS[,c(1,3)],pch=20,cex=0.25,col="darkgrey",xlab="n",ylab="Separation",main="Average separation for perfect classifiers",ylim=c(0,2.25),xlim=c(0,250),xaxp=c(0,250,10),yaxp=c(0,2.5,10))
lines(fit,col="black",lwd=1)
lines(fit.low,col="red",lwd=1)
lines(fit.up,col="red",lwd=1)
points(Resultados,col="blue",type="h")
points(Resultados,col="blue",pch=20)
text(Resultados[,1],Resultados[,2]+0.02,col="blue",labels=row.names(Resultados),cex=0.75)
abline(h=2.47,col="green")
text(20,2.6,col="green",labels="Global",cex=0.75)
```

```
Resultados %>%
  kbl() %>%
  kable_styling()
```

|  | Size | Separation |
| --- | --- | --- |
| LR | 95 | 1.981986 |
| glmnet.ext.3 | 42 | 1.492428 |
| glmnet.ext.5 | 53 | 1.203924 |
| glmnet.ext.10 | 59 | 1.120839 |
| intersection | 10 | 1.960728 |

### 5.4 CYM T2C vs CYM T2HW

```
tax_otu=as.data.frame(read_excel("TablaOTUs.xlsx"))[13:24,]
Samples=tax_otu$ID 
taxa=colnames(tax_otu[,3:dim(tax_otu)[2]])
OTUs=paste("OTU",1:(dim(tax_otu)[2]-2),sep="")

DF.0=tax_otu[,-c(1,2)]
row.names(DF.0)=Samples
colnames(DF.0)=OTUs

taxa.original=taxa
DF.0.original=DF.0
n.OTUs.original=dim(DF.0)[2]
indices=1:n.OTUs.original

hit=function(x){min(c(x,1))}
DF.0.hits=as.matrix(DF.0)
DF.0.hits[]=vapply(DF.0.hits, hit, numeric(1))

Unics=data.frame(
Sample=rep(NA,length(which(colSums(DF.0.hits)==1))), OTUs=names(colSums(DF.0)[which(colSums(DF.0.hits)==1)]), Reads=colSums(DF.0)[which(colSums(DF.0.hits)==1)],
            Proportions=rep(0,length(which(colSums(DF.0.hits)==1)))  
)
for (i in 1:length(Unics$OTUs)){
ii=which(DF.0.hits[,Unics$OTUs[i]]==1)  
Unics$Proportions[i]=DF.0[ii,Unics$OTUs[i]]/sum(DF.0[ii,])
Unics$Sample[i]=Samples[ii]}
Unics.Freqs=Unics[Unics$Proportions>=0.05/100,]
row.names(Unics.Freqs)=NULL

Miseria=as.vector(which(colSums(DF.0)<=2|colSums(DF.0.hits)==1))
DF.0=DF.0[,-Miseria]
taxa=taxa[-Miseria]
OTUs=OTUs[-Miseria]
indices=indices[-Miseria]

row.names(DF.0)=Samples

DF=zCompositions::cmultRepl(DF.0,method="GBM",output="p-counts",suppress.print=TRUE,z.warning=0.99)
```

```
DF.0=DF.0[7:12,]
DF=DF[7:12,]
DF.0.original=DF.0.original[7:12 ,]

Type=as.factor(c(rep("CYM_T2C", 3),rep("CYM_T2HW", 3)))
colors=c("purple","red")[Type]
```

After removing OTUs with 2 or fewer reads across the CAU samples and those appearing in only one of these samples, 2247 taxa remain.

The OTUs removed at this step that represent more than 0.05% of the sample in which they appear are:

```
Unics.Freqs[rev(order(Unics.Freqs$Proportions)),] %>%
  kbl() %>%
  kable_styling()
```

| Sample | OTUs | Reads | Proportions |
| --- | --- | --- | --- |
| CYM\_T2C\_1 | OTU2121 | 186 | 0.0020402 |

```
rm(tax_otu)
rm(DF.0.hits)
rm(Miseria)
rm(Unics.Freqs)
```

#### 5.4.1 How different are the samples?

```
HC=ClusterHC(DF,Grups=Type,colores=colors)
```

```
base=Sep(HC)
```

The two sample types are perfectly separated from the start. The separation between groups is 1.21.

We examine the proportion of OTU subsets of reasonable size that already classify them correctly.

```
> initial_seed=as.integer(Sys.time())
> print (initial_seed)
# [1] 1714640507
initial_seed %% 10000
# [1] 507
```

```
set.seed(507)
Experimento=function(i)
{
  ii=sample(dim(DF)[2],i,rep=FALSE)
  DFtemp=DF[,ii]
  CC=ClusterHC(DFtemp,dendrograma=FALSE,barplot=FALSE, Grups=Type,colores=colors)
  c(Encerts(CC$tabla,table(Type)),Sep(CC))
}
n=250
F.SS=c()
for (i in 10:1000)
  {
  print(i)
  EE=replicate(n,Experimento(i))
  EE.2=EE[2,which(EE[1,]==0)]
XX=replicate(1000,mean(sample(EE.2,n,replace=TRUE)))
  F.SS=rbind(F.SS, c(i,
                     length(EE.2)/250,
                      mean(EE.2),
                      quantile(XX,0.025),
                      quantile(XX,0.975)
  ))
  }
colnames(F.SS)=c("n","Proportion","Avg. sep.","Q_0.025","Q_0.975")
saveRDS(F.SS, file="Fraccio.Separadors.CYM.T2CvsT2HW.RData")
```

```
F.SS=readRDS("Fraccio.Separadors.CYM.T2CvsT2HW.RData")
```

The first plot shows, for each \(n\) up to 600, the proportion of samples of size \(n\) that perfectly classify CYM\_T2C and CYM\_T2HW (black curve), along with the 95% Clopper–Pearson confidence interval for that proportion (red curves). We used *smoothing splines* to smooth the curves.

```
F.SS_prop.Q_0.025=epitools::binom.exact(F.SS[,2]*250,250)$lower
F.SS_prop.Q_0.975=epitools::binom.exact(F.SS[,2]*250,250)$upper
fit = smooth.spline(F.SS[,1],F.SS[,2],cv=TRUE)
fit.low = smooth.spline(F.SS[,1],F.SS_prop.Q_0.025,cv=TRUE)
fit.up = smooth.spline(F.SS[,1],F.SS_prop.Q_0.975,cv=TRUE)


plot(F.SS[,1:2],pch=20,cex=0.25,col="darkgrey",xlab="n",ylab="Proportion",main="Proportion of perfect classifiers",xlim=c(0,1000),xaxp=c(0,1000,20),yaxp=c(0,1,10))
lines(fit,col="black",lwd=1)
lines(fit.low,col="red",lwd=1)
lines(fit.up,col="red",lwd=1)
```

The second plot shows, for each \(n\) up to 600, the estimated mean separation obtained with a sample of size \(n\) that perfectly classifies CYM\_T2C and CYM\_T2HW (black curve), along with the 95% confidence interval of this mean separation obtained via bootstrap (red curve).

```
fit = smooth.spline(F.SS[,1],F.SS[,3],cv=TRUE)
fit.low = smooth.spline(F.SS[,1],F.SS[,4],cv=TRUE)
fit.up = smooth.spline(F.SS[,1],F.SS[,5],cv=TRUE)

plot(F.SS[,c(1,3)],pch=20,cex=0.25,col="darkgrey",xlab="n",ylab="Separation",main="Average separation for perfect classifiers",xlim=c(0,1000),xaxp=c(0,1000,20),yaxp=c(0,2.5,10))
lines(fit,col="black",lwd=1)
lines(fit.low,col="red",lwd=1)
lines(fit.up,col="red",lwd=1)
abline(h=base,col="green")
```

`

#### 5.4.2 Taxa with maximum relevant log-ratios

We apply the LR approach exactly as described in the first comparison: OTUs are ranked by their log-ratio relevance, and we retain the subset maximizing the average absolute within-subset log-ratio association.

```
LRS=explore_logratios(DF,Type)
saveRDS(LRS, file="LRS_CYM_2C2HW.RData")
```

```
LRS=readRDS("LRS_CYM_2C2HW.RData")
```

```
assoc=rep(0,dim(DF)[2]-4)
for (m in 5:dim(DF)[2]){
    assoc[m-4]=sum(LRS$`association log-ratio with y`[1:m,1:m])/m^2
}
saveRDS(assoc, file="assoc_CYM_2C2HW.RData")
```

```
assoc=readRDS("assoc_CYM_2C2HW.RData")
plot(5:dim(DF)[2],assoc,type="b",xlab="m",ylab="Average goodness of the m most relevant." ,pch=20,cex=0.5)
```

```
m=4+which.max(assoc)
impLR=sort(as.numeric(LRS$`order of importance`[1:m]))

HC=ClusterHC(DF[,impLR],barplot=FALSE,Grups=Type,colores=colors)
```

```
LR.max=c(length(impLR),Sep(HC))
```

The maximum is attained at the set of 563 most logratio-relevant taxa.  
They perfectly separate the two sample types. The separation between groups is 2.15.

```
Significant=data.frame(
  OTUs=paste("OTU",1:n.OTUs.original,sep=""),
  taxa=taxa.original,
  Log_ratio.max=Indicador(impLR)
  )
```

#### 5.4.3 Taxa relevant for the bacterial signature (according to `coda_glmnet`)

As described above, `coda_glmnet` identifies a microbial signature by fitting a penalized log-contrast model and retaining OTUs with non-zero coefficients.

```
coda.glmnet=coda_glmnet(DF,Type,showPlots = FALSE)
```

```
## [1] "No variables are selected. The prediction and the signature plots are not displayed."
```

```
coda.glmnet$`signature plot`
```

```
## NULL
```

`coda_glmnet` does not find taxa with significant differences. We add random replicates. We test with 3, 5, and 10 replicates.

```
# 3 replicates
set.seed(5693)
M=3
repls=aldexCesc.clr(t(DF), conds=Type, mc.samples=M)@analysisData
```

```
## [1] "operating in serial mode"
```

```
repls=t(cbind(repls$CYM_T2C_1,repls$CYM_T2C_2,repls$CYM_T2C_3,repls$CYM_T2HW_1,repls$CYM_T2HW_2,repls$CYM_T2HW_3))
attr(repls,"dimnames")[[2]]=NULL
colnames(repls)=colnames(DF)
row.names(repls)=c(paste("CYM_T2C_1",1:M,sep="_"),
  paste("CYM_T2C_2",1:M,sep="_"),
  paste("CYM_T2C_3",1:M,sep="_"),
  paste("CYM_T2HW_1",1:M,sep="_"),
  paste("CYM_T2HW_2",1:M,sep="_"),
  paste("CYM_T2HW_3",1:M,sep="_"))
mostres=rbind(repls,easyCODA::CLR(DF)$LR)
rm(repls)
Type_ext=as.factor(c(rep("CYM_T2C", 3*M),rep("CYM_T2HW", 3*M),rep("CYM_T2C", 3),rep("CYM_T2HW", 3)))
colors_ext=c("purple","red")[Type_ext]
```

```
LogRat_mostres=logratios_matrix_clr(mostres)
saveRDS(LogRat_mostres, file="LogRat_mostres_CYM_2C2HW_3copies.RData")
```

```
LogRat_mostres=readRDS("LogRat_mostres_CYM_2C2HW_3copies.RData")
LogRat_mostres=list(LogRat_mostres[[1]],LogRat_mostres[[2]])

coda.glmnet.3=coda_glmnet_lr_bin(LogRat_mostres,Type_ext,taxa,showPlots = FALSE)
```

```
impGLMNET_ext.3=coda.glmnet.3$taxa.num
HC=ClusterHC(DF[,impGLMNET_ext.3],dendrograma=TRUE,barplot=FALSE,Grups=Type,colores=colors)
```

```
glmnet.ext.3=c(length(impGLMNET_ext.3),Sep(HC))
```

With 3 replicates, 81 significant taxa are found, perfectly separating the two groups, with a separation of 3.67.

```
Significant=cbind(Significant,
glmnet_sign.ext.3.T2C=Indicador(coda.glmnet.3$taxa.num[coda.glmnet.3$`log-contrast coefficients`>0]),
glmnet_sign.ext.3.T2HW=Indicador(coda.glmnet.3$taxa.num[coda.glmnet.3$`log-contrast coefficients`<0]))
```

```
# 5 replicates
set.seed(5693)
M=5
repls=aldexCesc.clr(t(DF), conds=Type, mc.samples=M)@analysisData
```

```
## [1] "operating in serial mode"
```

```
repls=t(cbind(repls$CYM_T2C_1,repls$CYM_T2C_2,repls$CYM_T2C_3,repls$CYM_T2HW_1,repls$CYM_T2HW_2,repls$CYM_T2HW_3))
attr(repls,"dimnames")[[2]]=NULL
colnames(repls)=colnames(DF)
row.names(repls)=c(paste("CYM_T2C_1",1:M,sep="_"),
  paste("CYM_T2C_2",1:M,sep="_"),
  paste("CYM_T2C_3",1:M,sep="_"),
  paste("CYM_T2HW_1",1:M,sep="_"),
  paste("CYM_T2HW_2",1:M,sep="_"),
  paste("CYM_T2HW_3",1:M,sep="_"))
mostres=rbind(repls,easyCODA::CLR(DF)$LR)
rm(repls)
Type_ext=as.factor(c(rep("CYM_T2C", 3*M),rep("CYM_T2HW", 3*M),rep("CYM_T2C", 3),rep("CYM_T2HW", 3)))
colors_ext=c("purple","red")[Type_ext]
```

```
LogRat_mostres=logratios_matrix_clr(mostres)
saveRDS(LogRat_mostres, file="LogRat_mostres_CYM_2C2HW_5copies.RData")
```

```
LogRat_mostres=readRDS("LogRat_mostres_CYM_2C2HW_5copies.RData")
LogRat_mostres[[1]]=LogRat_mostres[[1]][c(1:30,37:42),]
LogRat_mostres=list(LogRat_mostres[[1]],LogRat_mostres[[2]])

coda.glmnet.5=coda_glmnet_lr_bin(LogRat_mostres,Type_ext,taxa,showPlots = FALSE)
```

```
impGLMNET_ext.5=coda.glmnet.5$taxa.num
HC=ClusterHC(DF[,impGLMNET_ext.5],dendrograma=TRUE,barplot=FALSE,Grups=Type,colores=colors)
```

```
glmnet.ext.5=c(length(impGLMNET_ext.5),Sep(HC))
```

With 5 replicates, 84 significant taxa are found, perfectly separating the two groups, with a separation of 3.4.

```
Significant=cbind(Significant,
glmnet_sign.ext.5.T2C=Indicador(coda.glmnet.5$taxa.num[coda.glmnet.5$`log-contrast coefficients`>0]),
glmnet_sign.ext.5.T2HW=Indicador(coda.glmnet.5$taxa.num[coda.glmnet.5$`log-contrast coefficients`<0]))
```

```
set.seed(5693)
M=10
repls=aldexCesc.clr(t(DF), conds=Type, mc.samples=M)@analysisData
```

```
## [1] "operating in serial mode"
```

```
repls=t(cbind(repls$CYM_T2C_1,repls$CYM_T2C_2,repls$CYM_T2C_3,repls$CYM_T2HW_1,repls$CYM_T2HW_1,repls$CYM_T2HW_3))
attr(repls,"dimnames")[[2]]=NULL
colnames(repls)=colnames(DF)
row.names(repls)=c(paste("CYM_T2C_1",1:M,sep="_"),
  paste("CYM_T2C_2",1:M,sep="_"),
  paste("CYM_T2C_3",1:M,sep="_"),
  paste("CYM_T2HW_1",1:M,sep="_"),
  paste("CYM_T2HW_2",1:M,sep="_"),
  paste("CYM_T2HW_3",1:M,sep="_"))
mostres=rbind(repls,easyCODA::CLR(DF)$LR)
rm(repls)
Type_ext=as.factor(c(rep("CYM_T2C", 3*M),rep("CYM_T2HW", 3*M),rep("CYM_T2C", 3),rep("CYM_T2HW", 3)))
colors_ext=c("purple","red")[Type_ext]
```

```
LogRat_mostres=logratios_matrix_clr(mostres)
saveRDS(LogRat_mostres, file="LogRat_mostres_CYM_2C2HW_10copies.RData")
```

```
LogRat_mostres=readRDS("LogRat_mostres_CYM_2C2HW_10copies.RData")
LogRat_mostres[[1]]=LogRat_mostres[[1]][c(1:60,67:72),]
LogRat_mostres=list(LogRat_mostres[[1]],LogRat_mostres[[2]])

coda.glmnet.10=coda_glmnet_lr_bin(LogRat_mostres,Type_ext,taxa,showPlots = FALSE)
```

```
impGLMNET_ext.10=coda.glmnet.10$taxa.num
HC=ClusterHC(DF[,impGLMNET_ext.10],dendrograma=TRUE,barplot=FALSE,Grups=Type,colores=colors)
```

```
glmnet.ext.10=c(length(impGLMNET_ext.10),Sep(HC))
```

With 10 replicates, 82 significant taxa are found, perfectly separating the two groups, with a separation of 3.09.

```
Significant=cbind(Significant,
glmnet_sign.ext.10.T2C=Indicador(coda.glmnet.10$taxa.num[coda.glmnet.10$`log-contrast coefficients`>0]),
glmnet_sign.ext.10.T2HW=Indicador(coda.glmnet.10$taxa.num[coda.glmnet.10$`log-contrast coefficients`<0]))
```

#### 5.4.4 OTUs significant according to ALDeX

```
> initial_seed=as.integer(Sys.time())
> print (initial_seed)
# [1] 1711815195
initial_seed %% 10000
# [1] 5195
```

```
set.seed(5195)
repl_kw=aldexCesc.clr(t(DF), Type=Type, mc.samples=1000, verbose=FALSE)
aldex_kw=aldex.kw(repl_kw, verbose=FALSE)
#
saveRDS(repl_kw, file="repl_kw_2C2HW.RData")
saveRDS(aldex_kw, file="aldex_kw_2C2HW.RData")
```

```
p.valores_kw=readRDS("aldex_kw_2C2HW.RData")
```

```
length(p.valores_kw[p.valores_kw[,1]<0.01,1])
```

```
## [1] 0
```

```
length(p.valores_kw[p.valores_kw[,1]<0.05,1])
```

```
## [1] 29
```

```
length(p.valores_kw[p.valores_kw[,2]<0.01,2])
```

```
## [1] 0
```

```
length(p.valores_kw[p.valores_kw[,2]<0.01,2])
```

```
## [1] 0
```

```
sep.kw.noadjust.0.05=QQ.HC.noadjust(p.valores_kw,DF,Type,q=0.05,1,BP=TRUE)
```

There are no taxa with a Kruskal–Wallis adjusted p-value < 0.05. There are 29 with an unadjusted p-value < 0.05 (none < 0.01); in the absence of a better option, we select them. They perfectly separate the two groups, with a separation of 3.11.

```
Significant=cbind(Significant,
Aldex.kw.noadjust.0.05=Indicador(sep.kw.noadjust.0.05))
```

#### 5.4.5 Simper

```
DF.prop=DF.0.original/rowSums(DF.0.original)
SMP.prop=simper(DF.prop,group=Type)
indexos_SMP=SMP.prop$CYM_T2C_CYM_T2HW$ord[which(SMP.prop$CYM_T2C_CYM_T2HW$cusum<=0.7)]
OTUs_SMP=sort(attr(SMP.prop$CYM_T2C_CYM_T2HW$cusum[which(SMP.prop$CYM_T2C_CYM_T2HW$cusum<=0.7)],"names"))
OTUs_SMP.good=setdiff(OTUs_SMP,Unics$OTUs)
HC=ClusterHC(DF[,OTUs_SMP.good],dendrograma=TRUE,barplot=FALSE,Grups=Type,colores=colors)
simper.props=c(length(indexos_SMP),Sep(HC))
```

We obtain 120 significant taxa, which perfectly separate the two sample types.They include OTU2121, which we had previously removed because it appeared in only one sample. The separation between groups is 1.42.

```
Indicador.Gl=function(x,k=n.OTUs.original){
  xx=rep(0,k)
 xx[x]=1
  return(xx)
}
Significant=cbind(Significant,
simper.rel=Indicador.Gl(indexos_SMP))
Significant=cbind(Significant,
t(DF.prop))
```

#### 5.4.6 Summary

```
write.csv2(Significant,"OTU_Significativos_CYM_T2.csv",row.names=FALSE)
```

```
Resultados=rbind(
LR.max,
glmnet.ext.3,
glmnet.ext.5,
glmnet.ext.10,
sep.kw.noadjust.0.05,
simper.props
)
row.names(Resultados)=c(
"LR",
"glmnet.ext.3",
"glmnet.ext.5",
"glmnet.ext.10",
"Aldex",
"simper")
colnames(Resultados)=c("Size","Separation")
```

```
par(opar)
fit = smooth.spline(F.SS[,1],F.SS[,3],cv=TRUE)
fit.low = smooth.spline(F.SS[,1],F.SS[,4],cv=TRUE)
fit.up = smooth.spline(F.SS[,1],F.SS[,5],cv=TRUE)

plot(F.SS[,c(1,3)],pch=20,cex=0.25,col="darkgrey",xlab="n",ylab="Separation",main="Average separation for perfect classifiers",xlim=c(0,600),xaxp=c(0,600,12),yaxp=c(0,6,20),ylim=c(0,6))
lines(fit,col="black",lwd=1)
lines(fit.low,col="red",lwd=1)
lines(fit.up,col="red",lwd=1)
points(Resultados,col="blue",type="h")
points(Resultados,col="blue",pch=20)
text(Resultados[,1],Resultados[,2]+0.01,col="blue",labels=row.names(Resultados),cex=0.75)
```

Finally, we intersect the OTUs identified as significant according to LR and `coda_glmnet` with 3 replicates

```
Significant.capat=Significant[,c(1,2,3,4,5)]
Significant.capat=cbind(Significant.capat,Cuánto=rowSums(Significant.capat[,3:dim(Significant.capat)[2]]))
Significant.capat=Significant.capat[Significant.capat$Cuánto==2,]
```

```
DF.Imp=DF[,Significant.capat[,1]]
HC=ClusterHC(DF.Imp,dendrograma=TRUE,barplot=FALSE,Grups=Type,colores=colors)
```

There are 60 taxa. They are:

```
Significant.capat[,1:2]%>%
  kbl() %>%
  kable_styling()
```

|  | OTUs | taxa |
| --- | --- | --- |
| OTU4 | OTU4 | d\_\_Bacteria;p\_\_Bacteroidota;c\_\_Bacteroidia;o\_\_Flavobacteriales;f\_\_Flavobacteriaceae;g\_\_Robiginitalea;s\_\_bacterium\_AMSU |
| OTU6 | OTU6 | d\_\_Bacteria;p\_\_Bacteroidota;c\_\_Bacteroidia;o\_\_Flavobacteriales;f\_\_Flavobacteriaceae;g\_\_Maritimimonas;s\_\_uncultured\_Bacteroidetes |
| OTU19 | OTU19 | d\_\_Bacteria;p\_\_Chloroflexi;c\_\_Anaerolineae;o\_\_Anaerolineales;f\_\_Anaerolineaceae;g**uncultured;** |
| OTU25 | OTU25 | d\_\_Archaea;p\_\_Asgardarchaeota;c\_\_Heimdallarchaeia;o\_\_Heimdallarchaeia;f\_\_Heimdallarchaeia;g\_\_Heimdallarchaeia;s\_\_uncultured\_archaeon |
| OTU26 | OTU26 | d\_\_Bacteria;p\_\_Chloroflexi;c\_\_Anaerolineae;o\_\_SBR1031;f\_\_SBR1031;g\_\_SBR1031;s\_\_uncultured\_organism |
| OTU27 | OTU27 | d\_\_Bacteria;p\_\_Bacteroidota;c\_\_Bacteroidia;o\_\_Flavobacteriales;f\_\_Flavobacteriaceae;g\_\_Ulvibacter;s\_\_Aureitalea\_sp. |
| OTU31 | OTU31 | d\_\_Bacteria;p\_\_Proteobacteria;c\_\_Gammaproteobacteria;o**Ectothiorhodospirales;**;**;** |
| OTU34 | OTU34 | d\_\_Bacteria;p\_\_Bacteroidota;c\_\_Bacteroidia;o\_\_Flavobacteriales;f\_\_Flavobacteriaceae;g**Maribacter;** |
| OTU48 | OTU48 | d\_\_Bacteria;p\_\_Patescibacteria;c\_\_Parcubacteria;**;**;**;** |
| OTU56 | OTU56 | d\_\_Bacteria;p\_\_Proteobacteria;c\_\_Gammaproteobacteria;o\_\_Arenicellales;f\_\_Arenicellaceae;g**uncultured;** |
| OTU78 | OTU78 | d\_\_Bacteria;p\_\_Spirochaetota;c\_\_Spirochaetia;o\_\_Spirochaetales;f\_\_Spirochaetaceae;g**Spirochaeta;** |
| OTU85 | OTU85 | d\_\_Bacteria;p**Chloroflexi;**;**;**;**;** |
| OTU94 | OTU94 | d\_\_Bacteria;p\_\_Spirochaetota;c\_\_Spirochaetia;o\_\_Spirochaetales;f\_\_Spirochaetaceae;g\_\_Spirochaeta;s\_\_uncultured\_bacterium |
| OTU102 | OTU102 | d\_\_Bacteria;p\_\_Bacteroidota;c\_\_Ignavibacteria;o\_\_Ignavibacteriales;f\_\_Melioribacteraceae;g\_\_IheB3-7;s\_\_uncultured\_Chlorobi |
| OTU107 | OTU107 | d\_\_Bacteria;p\_\_Actinobacteriota;c\_\_WCHB1-81;o\_\_WCHB1-81;f\_\_WCHB1-81;g**WCHB1-81;** |
| OTU118 | OTU118 | d\_\_Archaea;p\_\_Thermoplasmatota;c\_\_Thermoplasmata;o\_\_Marine\_Benthic\_Group\_D\_and\_DHVEG-1;f\_\_Marine\_Benthic\_Group\_D\_and\_DHVEG-1;g\_\_Marine\_Benthic\_Group\_D\_and\_DHVEG-1;s\_\_uncultured\_archaeon |
| OTU123 | OTU123 | d\_\_Bacteria;p\_\_Planctomycetota;c\_\_Planctomycetes;o\_\_Pirellulales;f\_\_Pirellulaceae;g**Pir4\_lineage;** |
| OTU126 | OTU126 | d\_\_Bacteria;p\_\_Bacteroidota;c\_\_Bacteroidia;o\_\_Chitinophagales;f\_\_Saprospiraceae;g**uncultured;** |
| OTU133 | OTU133 | d\_\_Bacteria;p\_\_Desulfobacterota;c\_\_Desulfobacteria;o\_\_Desulfatiglandales;f\_\_Desulfatiglandaceae;g**Desulfatiglans;** |
| OTU136 | OTU136 | d\_\_Bacteria;p\_\_Patescibacteria;c\_\_Parcubacteria;o\_\_Candidatus\_Kaiserbacteria;f\_\_Candidatus\_Kaiserbacteria;g\_\_Candidatus\_Kaiserbacteria;s\_\_uncultured\_bacterium |
| OTU137 | OTU137 | d\_\_Archaea;p\_\_Thermoplasmatota;c\_\_Thermoplasmata;o\_\_Marine\_Benthic\_Group\_D\_and\_DHVEG-1;f\_\_Marine\_Benthic\_Group\_D\_and\_DHVEG-1;g**Marine\_Benthic\_Group\_D\_and\_DHVEG-1;** |
| OTU143 | OTU143 | d\_\_Archaea;p\_\_Crenarchaeota;c\_\_Bathyarchaeia;o\_\_Bathyarchaeia;f\_\_Bathyarchaeia;g**Bathyarchaeia;** |
| OTU144 | OTU144 | d\_\_Bacteria;p\_\_Bacteroidota;c\_\_Bacteroidia;o\_\_Flavobacteriales;f\_\_Flavobacteriaceae;g**Aquibacter;** |
| OTU163 | OTU163 | d\_\_Bacteria;p\_\_Modulibacteria;c\_\_Moduliflexia;o\_\_Moduliflexales;f\_\_Moduliflexaceae;g\_\_Moduliflexaceae;s\_\_uncultured\_bacterium |
| OTU168 | OTU168 | d\_\_Bacteria;p\_\_Proteobacteria;c\_\_Gammaproteobacteria;o\_\_Thiomicrospirales;f\_\_Thiomicrospiraceae;g**endosymbionts;** |
| OTU174 | OTU174 | d\_\_Bacteria;p\_\_Bacteroidota;c\_\_Bacteroidia;o\_\_Bacteroidales;f\_\_SB-5;g\_\_SB-5;s\_\_bacterium\_YC-LK-LKJ31 |
| OTU178 | OTU178 | d\_\_Bacteria;p\_\_Bacteroidota;c\_\_Bacteroidia;o\_\_Flavobacteriales;f\_\_Flavobacteriaceae;g**Winogradskyella;** |
| OTU181 | OTU181 | d\_\_Bacteria;p\_\_Chloroflexi;c\_\_Anaerolineae;**;**;**;** |
| OTU182 | OTU182 | d\_\_Bacteria;p\_\_Chloroflexi;c\_\_Anaerolineae;o\_\_uncultured;f\_\_uncultured;g**uncultured;** |
| OTU186 | OTU186 | d\_\_Bacteria;p\_\_LCP-89;c\_\_LCP-89;o\_\_LCP-89;f\_\_LCP-89;g\_\_LCP-89;s\_\_uncultured\_bacterium |
| OTU200 | OTU200 | d\_\_Bacteria;p\_\_Bacteroidota;c\_\_Bacteroidia;o\_\_Chitinophagales;f\_\_Saprospiraceae;**;** |
| OTU212 | OTU212 | d\_\_Bacteria;p\_\_Bdellovibrionota;c\_\_Bdellovibrionia;o\_\_Bacteriovoracales;f\_\_Bacteriovoracaceae;g\_\_uncultured;s\_\_uncultured\_bacterium |
| OTU216 | OTU216 | d\_\_Bacteria;p\_\_Actinobacteriota;c\_\_WCHB1-81;o\_\_WCHB1-81;f\_\_WCHB1-81;g\_\_WCHB1-81;s\_\_uncultured\_bacterium |
| OTU224 | OTU224 | d\_\_Bacteria;p\_\_Proteobacteria;c\_\_Gammaproteobacteria;o\_\_Chromatiales;f\_\_Sedimenticolaceae;g\_\_uncultured;s\_\_uncultured\_bacterium |
| OTU241 | OTU241 | d\_\_Bacteria;p\_\_Chloroflexi;c\_\_Anaerolineae;o\_\_Anaerolineales;f\_\_Anaerolineaceae;g\_\_uncultured;s\_\_uncultured\_Chloroflexi |
| OTU283 | OTU283 | d\_\_Bacteria;p\_\_Calditrichota;c\_\_Calditrichia;o\_\_Calditrichales;f\_\_Calditrichaceae;**;** |
| OTU296 | OTU296 | d\_\_Bacteria;p\_\_Patescibacteria;c\_\_Parcubacteria;o\_\_Candidatus\_Kaiserbacteria;f\_\_Candidatus\_Kaiserbacteria;g\_\_Candidatus\_Kaiserbacteria;s\_\_uncultured\_prokaryote |
| OTU309 | OTU309 | d\_\_Bacteria;p\_\_Bacteroidota;c\_\_Bacteroidia;o\_\_Chitinophagales;f\_\_Saprospiraceae;g**Lewinella;** |
| OTU329 | OTU329 | d\_\_Bacteria;p\_\_Bacteroidota;c\_\_Bacteroidia;o\_\_Flavobacteriales;f\_\_Flavobacteriaceae;g\_\_Aquibacter;s\_\_uncultured\_marine |
| OTU347 | OTU347 | d\_\_Bacteria;p\_\_Chloroflexi;c\_\_Dehalococcoidia;**;**;**;** |
| OTU372 | OTU372 | d\_\_Bacteria;p\_\_Proteobacteria;c\_\_Gammaproteobacteria;o\_\_Oceanospirillales;f\_\_Pseudohongiellaceae;g**Pseudohongiella;** |
| OTU412 | OTU412 | d\_\_Bacteria;p\_\_Desulfobacterota;c\_\_Desulfobulbia;o**Desulfobulbales;**;**;** |
| OTU426 | OTU426 | d\_\_Bacteria;p\_\_Verrucomicrobiota;c\_\_Omnitrophia;o\_\_Omnitrophales;f\_\_Omnitrophaceae;g\_\_Candidatus\_Omnitrophus;s\_\_uncultured\_bacterium |
| OTU433 | OTU433 | d\_\_Bacteria;p\_\_Bacteroidota;c\_\_Bacteroidia;o\_\_Chitinophagales;f\_\_Saprospiraceae;g\_\_Lewinella;s\_\_uncultured\_marine |
| OTU490 | OTU490 | d\_\_Bacteria;p\_\_Bdellovibrionota;c\_\_Bdellovibrionia;o\_\_Bacteriovoracales;f\_\_Bacteriovoracaceae;g**Peredibacter;** |
| OTU516 | OTU516 | d\_\_Bacteria;p\_\_Bdellovibrionota;c\_\_Bdellovibrionia;o\_\_Bdellovibrionales;f\_\_Bdellovibrionaceae;g**Bdellovibrio;** |
| OTU565 | OTU565 | d\_\_Bacteria;p\_\_Bacteroidota;c\_\_Bacteroidia;o\_\_Chitinophagales;f\_\_Saprospiraceae;g\_\_Portibacter;s\_\_uncultured\_Bacteroidetes |
| OTU574 | OTU574 | d\_\_Bacteria;p\_\_Planctomycetota;c\_\_Planctomycetes;o\_\_Pirellulales;f\_\_Pirellulaceae;g\_\_uncultured;s\_\_uncultured\_organism |
| OTU597 | OTU597 | d\_\_Bacteria;p\_\_Acidobacteriota;c\_\_Thermoanaerobaculia;o\_\_Thermoanaerobaculales;f\_\_Thermoanaerobaculaceae;g\_\_Subgroup\_23;s\_\_uncultured\_bacterium |
| OTU622 | OTU622 | d\_\_Bacteria;p\_\_Bacteroidota;c\_\_Bacteroidia;o\_\_Chitinophagales;f\_\_uncultured;g\_\_uncultured;s\_\_uncultured\_Bacteroidetes |
| OTU625 | OTU625 | d\_\_Bacteria;p\_\_Proteobacteria;c\_\_Gammaproteobacteria;o\_\_pItb-vmat-80;f\_\_pItb-vmat-80;g**pItb-vmat-80;** |
| OTU659 | OTU659 | d\_\_Bacteria;p\_\_Proteobacteria;c\_\_Alphaproteobacteria;o\_\_Rickettsiales;f\_\_Mitochondria;g\_\_Mitochondria;s\_\_uncultured\_bacterium |
| OTU785 | OTU785 | d\_\_Archaea;p\_\_Asgardarchaeota;c\_\_Odinarchaeia;o\_\_Odinarchaeia;f\_\_Odinarchaeia;g\_\_Odinarchaeia;s\_\_uncultured\_crenarchaeote |
| OTU854 | OTU854 | d\_\_Bacteria;p\_\_Verrucomicrobiota;c\_\_Verrucomicrobiae;o\_\_Opitutales;f\_\_Puniceicoccaceae;g**Lentimonas;** |
| OTU980 | OTU980 | d\_\_Bacteria;p\_\_Bacteroidota;c\_\_Bacteroidia;o\_\_Chitinophagales;f\_\_Saprospiraceae;g\_\_Phaeodactylibacter;s\_\_uncultured\_organism |
| OTU1088 | OTU1088 | d\_\_Bacteria;p\_\_Proteobacteria;c\_\_Gammaproteobacteria;o\_\_Cellvibrionales;f\_\_Halieaceae;g**Congregibacter;** |
| OTU1502 | OTU1502 | d\_\_Archaea;p\_\_Thermoplasmatota;c\_\_Thermoplasmatota;o\_\_Thermoplasmatota;f\_\_Thermoplasmatota;g**uncultured;** |
| OTU1532 | OTU1532 | d\_\_Bacteria;p\_\_Proteobacteria;c\_\_Gammaproteobacteria;o\_\_Chromatiales;f\_\_Chromatiaceae;g\_\_Candidatus\_Thiobios;s\_\_uncultured\_organism |
| OTU1539 | OTU1539 | d\_\_Bacteria;p\_\_Acidobacteriota;c\_\_Vicinamibacteria;o\_\_Subgroup\_17;f\_\_Subgroup\_17;g**Subgroup\_17;** |
| OTU2370 | OTU2370 | d\_\_Bacteria;p\_\_Bdellovibrionota;c\_\_Bdellovibrionia;o\_\_Bacteriovoracales;f\_\_Bacteriovoracaceae;g\_\_uncultured;s\_\_uncultured\_deep-sea |

The separation between groups is 4.31.

We update the plot with the separations:

```
intersección=c(dim(Significant.capat)[1],Sep(HC))
Resultados=rbind(Resultados,intersección)
row.names(Resultados)=c(
"LR",
"glmnet.ext.3",
"glmnet.ext.5",
"glmnet.ext.10",
"Aldex",
"simper",
"Intersection")
colnames(Resultados)=c("Size","Separation")
```

```
plot(F.SS[-1,c(1,3)],pch=20,cex=0.25,col="darkgrey",xlab="n",ylab="Separation",main="Average separation for perfect classifiers",xlim=c(0,150),xaxp=c(0,150,15),yaxp=c(0,6,12),ylim=c(0,6))
lines(fit,col="black",lwd=1)
lines(fit.low,col="red",lwd=1)
lines(fit.up,col="red",lwd=1)
points(Resultados[-dim(Resultados)[1],],col="blue",type="h")
points(Resultados[-dim(Resultados)[1],],col="blue",pch=20)
points(intersección[1],intersección[2],col="purple",type="h")
points(intersección[1],intersección[2],col="purple",pch=20)
text(Resultados[-dim(Resultados)[1],1],Resultados[-dim(Resultados)[1],2]+0.1,col="blue",labels=row.names(Resultados)[-dim(Resultados)[1]],cex=0.75)
text(intersección[1],intersección[2]+0.1,col="purple",labels="intersección",cex=0.75)

abline(h=base,col="green")
text(20,base+0.1,col="green",labels="Global",cex=0.75)
```

```
Resultados %>%
  kbl() %>%
  kable_styling()
```

|  | Size | Separation |
| --- | --- | --- |
| LR | 563 | 2.150788 |
| glmnet.ext.3 | 81 | 3.668474 |
| glmnet.ext.5 | 84 | 3.399211 |
| glmnet.ext.10 | 82 | 3.085298 |
| Aldex | 29 | 3.114325 |
| simper | 120 | 1.415174 |
| Intersection | 60 | 4.306244 |

### 5.5 CAU T0 vs CAU T1

```
tax_otu=as.data.frame(read_excel("TablaOTUs.xlsx"))[1:12,]

Samples=tax_otu$ID 
taxa=colnames(tax_otu[,3:dim(tax_otu)[2]])
OTUs=paste("OTU",1:(dim(tax_otu)[2]-2),sep="")

DF.0=tax_otu[,-c(1,2)]
row.names(DF.0)=Samples
colnames(DF.0)=OTUs

taxa.original=taxa
DF.0.original=DF.0
n.OTUs.original=dim(DF.0)[2]

Miseria=as.vector(which(colSums(DF.0[1:6,])<=3))
DF.0=DF.0[,-Miseria]
taxa=taxa[-Miseria]
OTUs=OTUs[-Miseria]

hit=function(x){min(c(x,1))}
DF.0.hits=as.matrix(DF.0)
DF.0.hits[]=vapply(DF.0.hits, hit, numeric(1))

Unics=data.frame(
Sample=rep(NA,length(which(colSums(DF.0.hits)==1))), OTUs=names(colSums(DF.0)[which(colSums(DF.0.hits)==1)]), Reads=colSums(DF.0)[which(colSums(DF.0.hits)==1)],
            Proportions=rep(0,length(which(colSums(DF.0.hits)==1)))  
)
for (i in 1:length(Unics$OTUs)){
ii=which(DF.0.hits[,Unics$OTUs[i]]==1)  
Unics$Proportions[i]=DF.0[ii,Unics$OTUs[i]]/sum(DF.0[ii,])
Unics$Sample[i]=Samples[ii]}
Unics.Freqs=Unics[Unics$Proportions>=0.05/100,]
row.names(Unics.Freqs)=NULL


DF.0=DF.0[,-which(colSums(DF.0.hits)==1)]
taxa=taxa[-which(colSums(DF.0.hits)==1)]
OTUs=OTUs[-which(colSums(DF.0.hits)==1)]

row.names(DF.0)=Samples

DF=zCompositions::cmultRepl(DF.0,method="GBM",output="p-counts",suppress.print=TRUE,z.warning=0.99)
```

```
DF=DF[1:6,]
DF.0=DF.0[1:6,]
DF.0.original=DF.0.original[1:6 ,]

Type=as.factor(c(rep("CAU_T0", 3),rep("CAU_T1", 3)))
colors=c("blue","green")[Type]
```

After removing OTUs with 2 or fewer reads across the samples and those appearing in only one of these samples, 1194 taxa remain.

The OTUs removed at this step that represent more than 0.05% of the sample in which they appear are:

```
Unics.Freqs[rev(order(Unics.Freqs$Proportions)),] %>%
  kbl() %>%
  kable_styling()
```

| Sample | OTUs | Reads | Proportions |
| --- | --- | --- | --- |
| CAU\_T0\_2 | OTU966 | 55 | 0.0012226 |

```
rm(tax_otu)
rm(DF.0.hits)
rm(Miseria)
rm(Unics.Freqs)
```

#### 5.5.1 How different are the samples?

```
HC=ClusterHC(DF,Grups=Type,colores=colors)
```

```
base=Sep(HC)
```

The two sample types are not initially separated.

We now examine the proportion of OTU subsets of reasonable size that already classify them correctly.

```
> initial_seed=as.integer(Sys.time())
> print (initial_seed)
# [1] 1717749309
initial_seed %% 10000
# [1] 9309
```

```
set.seed(9309)
Experimento=function(i)
{
  ii=sample(dim(DF)[2],i,rep=FALSE)
  DFtemp=DF[,ii]
  CC=ClusterHC(DFtemp,dendrograma=FALSE,barplot=FALSE, Grups=Type,colores=colors)
  c(Encerts(CC$tabla,table(Type)),Sep(CC))
}
n=500
F.SS=c()
for (i in 10:600) 
  {
  print(i)
  EE=replicate(n,Experimento(i))
  EE.2=EE[2,which(EE[1,]==0)]
  if(length(which(EE[1,]==0))>0){
XX=replicate(1000,mean(sample(EE.2,n,replace=TRUE)))
  F.SS=rbind(F.SS, c(i,
                     length(EE.2)/250,
                      mean(EE.2),
                      quantile(XX,0.025),
                      quantile(XX,0.975)
  ))}
    else {
    F.SS=rbind(F.SS, c(i,
                     0,
                      NA,
                      NA,
                      NA))
  }
  }
colnames(F.SS)=c("n","Proportion","Avg. sep.","Q_0.025","Q_0.975")
saveRDS(F.SS, file="Fraccio.Separadors.CAU.T0vsT1.RData")
```

```
F.SS=readRDS("Fraccio.Separadors.CAU.T0vsT1.RData")
```

The first plot shows, for each \(n\) up to 500, the proportion of samples of size \(n\) that perfectly classify CAU\_T0 and CAU\_T1 (black curve), along with the 95% Clopper–Pearson confidence interval for that proportion (red curves). We used *smoothing splines* to smooth the curves.

```
F.SS_prop.Q_0.025=epitools::binom.exact(F.SS[,2]*250,250)$lower
F.SS_prop.Q_0.975=epitools::binom.exact(F.SS[,2]*250,250)$upper
fit = smooth.spline(F.SS[,1],F.SS[,2],cv=TRUE)
fit.low = smooth.spline(F.SS[,1],F.SS_prop.Q_0.025,cv=TRUE)
fit.up = smooth.spline(F.SS[,1],F.SS_prop.Q_0.975,cv=TRUE)


plot(F.SS[,1:2],pch=20,cex=0.25,col="darkgrey",xlab="n",ylab="Proportion",main="Proportion of perfect classifiers",ylim=c(0,0.1),xlim=c(0,600),xaxp=c(0,600,12),yaxp=c(0,0.1,10))
lines(fit,col="black",lwd=1)
lines(fit.low,col="red",lwd=1)
lines(fit.up,col="red",lwd=1)
```

The second plot shows, for each \(n\) up to 600, the estimated mean separation obtained with a sample of size \(n\) that perfectly classifies CAU\_T0 and CAU\_T1 (black curve), along with the 95% confidence interval of this mean separation obtained via bootstrap (red curve).

```
fit = smooth.spline(F.SS[!is.na(F.SS[,3]),1],F.SS[!is.na(F.SS[,3]),3],cv=TRUE)
fit.low = smooth.spline(F.SS[!is.na(F.SS[,3]),1],F.SS[!is.na(F.SS[,3]),4],cv=TRUE)
fit.up = smooth.spline(F.SS[!is.na(F.SS[,3]),1],F.SS[!is.na(F.SS[,3]),5],cv=TRUE)

plot(F.SS[,c(1,3)],pch=20,cex=0.25,col="darkgrey",xlab="n",ylab="Separation",main="Average separation for perfect classifiers",xlim=c(0,600),xaxp=c(0,600,12),yaxp=c(0,1.5,15))
lines(fit,col="black",lwd=1)
lines(fit.low,col="red",lwd=1)
lines(fit.up,col="red",lwd=1)
```

`

#### 5.5.2 Taxa with maximum relevant log-ratios

We apply the LR approach exactly as described in the first comparison: OTUs are ranked by their log-ratio relevance, and we retain the subset maximizing the average absolute within-subset log-ratio association.

```
LRS=explore_logratios(DF,Type)
saveRDS(LRS, file="LRS_CAU_T0T1.RData")
```

```
LRS=readRDS("LRS_CAU_T0T1.RData")

assoc=rep(0,dim(DF)[2]-4)
for (m in 5:dim(DF)[2]){
    assoc[m-4]=sum(LRS$`association log-ratio with y`[1:m,1:m])/m^2
}

plot(5:dim(DF)[2],assoc,type="b",xlab="m",ylab="Average goodness of the m most relevant." ,pch=20,cex=0.5)
```

```
m=4+which.max(assoc)
impLR=sort(as.numeric(LRS$`order of importance`[1:m]))

HC=ClusterHC(DF[,impLR],barplot=FALSE,Grups=Type,colores=colors)
```

```
LR.max=c(length(impLR),Sep(HC))
```

The maximum is attained at the set of 24 most logratio-relevant taxa.  
They perfectly separate the two sample types. The separation between groups is 2.3.

```
Significant=data.frame(
  OTUs=paste("OTU",1:n.OTUs.original,sep=""),
  taxa=taxa.original,
  Log_ratio.max=Indicador(impLR))
```

#### 5.5.3 Taxa relevant for the bacterial signature (according to `coda_glmnet`)

```
coda.glmnet=coda_glmnet(DF,Type,showPlots = FALSE)
```

```
## [1] "No variables are selected. The prediction and the signature plots are not displayed."
```

```
coda.glmnet$`signature plot`
```

```
## NULL
```

`coda_glmnet` does not find taxa with significant differences. We add random replicates. We test with 3, 5, and 10 replicates.

```
> initial_seed=as.integer(Sys.time())
> print (initial_seed)
# [1]  1717764843
initial_seed %% 10000
# [1]  4843
```

```
# 3 replicates
set.seed(4843)
M=3
repls=aldexCesc.clr(t(DF), conds=Type, mc.samples=M)@analysisData
```

```
## [1] "operating in serial mode"
```

```
repls=t(cbind(repls$CAU_T0_1,repls$CAU_T0_2,repls$CAU_T0_3,repls$CAU_T1_1,repls$CAU_T1_2,repls$CAU_T1_3))
attr(repls,"dimnames")[[2]]=NULL
colnames(repls)=colnames(DF)
row.names(repls)=c(paste("CAU_T0_1",1:M,sep="_"),
  paste("CAU_T0_2",1:M,sep="_"),
  paste("CAU_T0_3",1:M,sep="_"),
  paste("CAU_T1_1",1:M,sep="_"),
  paste("CAU_T1_2",1:M,sep="_"),
  paste("CAU_T1_3",1:M,sep="_"))
mostres=rbind(repls,easyCODA::CLR(DF)$LR)
rm(repls)
Type_ext=as.factor(c(rep("CAU_T0", 3*M),rep("CAU_T1", 3*M),rep("CAU_T0", 3),rep("CAU_T1", 3)))
colors_ext=c("blue","green")[Type_ext]
```

```
LogRat_mostres=logratios_matrix_clr(mostres)
LogRat_mostres=list(LogRat_mostres[[1]],LogRat_mostres[[2]])
saveRDS(LogRat_mostres, file="LogRat_mostres_CAU_T0T1_3copies.RData")
```

```
LogRat_mostres=readRDS("LogRat_mostres_CAU_T0T1_3copies.RData")
coda.glmnet.3=coda_glmnet_lr_bin(LogRat_mostres,Type_ext,DF,showPlots = FALSE)
```

```
impGLMNET_ext.3=coda.glmnet.3$taxa.num
HC=ClusterHC(DF[,impGLMNET_ext.3],dendrograma=TRUE,barplot=FALSE,Grups=Type,colores=colors)
```

```
glmnet.ext.3=c(length(impGLMNET_ext.3),Sep(HC))
```

With 3 replicates, 45 significant taxa are found, perfectly separating the two groups, with a separation of 0.95.

```
Significant=cbind(Significant,
glmnet_sign.ext.3.T0=Indicador(coda.glmnet.3$taxa.num[coda.glmnet.3$`log-contrast coefficients`>0]),
glmnet_sign.ext.3.T1=Indicador(coda.glmnet.3$taxa.num[coda.glmnet.3$`log-contrast coefficients`<0]))
```

```
# 5 replicates
set.seed(4843)
M=5
repls=aldexCesc.clr(t(DF), conds=Type, mc.samples=M)@analysisData
```

```
## [1] "operating in serial mode"
```

```
repls=t(cbind(repls$CAU_T0_1,repls$CAU_T0_2,repls$CAU_T0_3,repls$CAU_T1_1,repls$CAU_T1_2,repls$CAU_T1_3))
attr(repls,"dimnames")[[2]]=NULL
colnames(repls)=colnames(DF)
row.names(repls)=c(paste("CAU_T0_1",1:M,sep="_"),
  paste("CAU_T0_2",1:M,sep="_"),
  paste("CAU_T0_3",1:M,sep="_"),
  paste("CAU_T1_1",1:M,sep="_"),
  paste("CAU_T1_2",1:M,sep="_"),
  paste("CAU_T1_3",1:M,sep="_"))
mostres=rbind(repls,easyCODA::CLR(DF)$LR)
rm(repls)
Type_ext=as.factor(c(rep("CAU_T0", 3*M),rep("CAU_T1", 3*M),rep("CAU_T0", 3),rep("CAU_T1", 3)))
colors_ext=c("blue","green")[Type_ext]
```

```
LogRat_mostres=logratios_matrix_clr(mostres)
LogRat_mostres=list(LogRat_mostres[[1]],LogRat_mostres[[2]])
saveRDS(LogRat_mostres, file="LogRat_mostres_CAU_T0T1_5copies.RData")
```

```
LogRat_mostres=readRDS("LogRat_mostres_CAU_T0T1_5copies.RData")
coda.glmnet.5=coda_glmnet_lr_bin(LogRat_mostres,Type_ext,DF,showPlots = FALSE)
```

```
impGLMNET_ext.5=coda.glmnet.5$taxa.num
HC=ClusterHC(DF[,impGLMNET_ext.5],dendrograma=TRUE,barplot=FALSE,Grups=Type,colores=colors)
```

```
glmnet.ext.5=c(length(impGLMNET_ext.5),Sep(HC))
```

With 5 replicates, 51 significant taxa are found, perfectly separating the two groups, with a separation of 0.88.

```
Significant=cbind(Significant,
glmnet_sign.ext.5.T0=Indicador(coda.glmnet.5$taxa.num[coda.glmnet.5$`log-contrast coefficients`>0]),
glmnet_sign.ext.5.T1=Indicador(coda.glmnet.5$taxa.num[coda.glmnet.5$`log-contrast coefficients`<0]))
```

```
# 10 replicates
set.seed(4843)
M=10
repls=aldexCesc.clr(t(DF), conds=Type, mc.samples=M)@analysisData
```

```
## [1] "operating in serial mode"
```

```
repls=t(cbind(repls$CAU_T0_1,repls$CAU_T0_2,repls$CAU_T0_3,repls$CAU_T1_1,repls$CAU_T1_2,repls$CAU_T1_3))
attr(repls,"dimnames")[[2]]=NULL
colnames(repls)=colnames(DF)
row.names(repls)=c(paste("CAU_T0_1",1:M,sep="_"),
  paste("CAU_T0_2",1:M,sep="_"),
  paste("CAU_T0_3",1:M,sep="_"),
  paste("CAU_T1_1",1:M,sep="_"),
  paste("CAU_T1_2",1:M,sep="_"),
  paste("CAU_T1_3",1:M,sep="_"))
mostres=rbind(repls,easyCODA::CLR(DF)$LR)
rm(repls)
Type_ext=as.factor(c(rep("CAU_T0", 3*M),rep("CAU_T1", 3*M),rep("CAU_T0", 3),rep("CAU_T1", 3)))
colors_ext=c("blue","green")[Type_ext]
```

```
LogRat_mostres=logratios_matrix_clr(mostres)
LogRat_mostres=list(LogRat_mostres[[1]],LogRat_mostres[[2]])
saveRDS(LogRat_mostres, file="LogRat_mostres_CAU_T0T1_10copies.RData")
```

```
LogRat_mostres=readRDS("LogRat_mostres_CAU_T0T1_10copies.RData")
coda.glmnet.10=coda_glmnet_lr_bin(LogRat_mostres,Type_ext,DF,showPlots = FALSE)
```

```
impGLMNET_ext.10=coda.glmnet.10$taxa.num
HC=ClusterHC(DF[,impGLMNET_ext.10],dendrograma=TRUE,barplot=FALSE,Grups=Type,colores=colors)
```

```
glmnet.ext.10=c(length(impGLMNET_ext.10),Sep(HC))
```

With 10 replicates, 49 significant taxa are found, but they do not classify well the samples.

```
Significant=cbind(Significant,
glmnet_sign.ext.10.T0=Indicador(coda.glmnet.5$taxa.num[coda.glmnet.10$`log-contrast coefficients`>0]),
glmnet_sign.ext.10.T1=Indicador(coda.glmnet.5$taxa.num[coda.glmnet.10$`log-contrast coefficients`<0]))
```

#### 5.5.4 OTUs significant according to ALDeX

```
> initial_seed=as.integer(Sys.time())
> print (initial_seed)
# [1] 1717772151
initial_seed %% 10000
# [1] 2151
```

```
set.seed(2151)
repl_kw=aldexCesc.clr(t(DF), conds=Type, mc.samples=1000, verbose=FALSE)
aldex_kw=aldex.kw(repl_kw, verbose=FALSE)
#
saveRDS(aldex_kw, file="aldex_kw_CAU_T0T1.RData")
```

```
p.valores_kw=readRDS("aldex_kw_CAU_T0T1.RData")
```

```
length(p.valores_kw[p.valores_kw[,1]<0.05,2])
```

```
## [1] 0
```

There are no taxa with a p-value < 0.05, even without adjustment.

#### 5.5.5 Simper

The significant taxa identified by `simper` do not classify the samples well:

```
DF.prop=DF.0.original/rowSums(DF.0.original)
SMP.prop=simper(DF.prop,group=Type)
indexos_SMP=SMP.prop$CAU_T0_CAU_T1$ord[which(SMP.prop$CAU_T0_CAU_T1$cusum<=0.7)]
OTUs_SMP=sort(attr(SMP.prop$CAU_T0_CAU_T1$cusum[which(SMP.prop$CAU_T0_CAU_T1$cusum<=0.7)],"names"))
OTUs_SMP.good=setdiff(OTUs_SMP,Unics$OTUs)
HC=ClusterHC(DF[,OTUs_SMP.good],dendrograma=TRUE,barplot=FALSE,Grups=Type,colores=colors)
```

```
Significant=cbind(Significant,
t(DF.prop))
```

#### 5.5.6 Summary

```
write.csv2(Significant,"OTU_Significativos_CAU_T0T1.csv",row.names=FALSE)
```

```
Resultados=rbind(
LR.max,
glmnet.ext.3,
glmnet.ext.5,
glmnet.ext.10
)
row.names(Resultados)=c(
"LR",
"glmnet.ext.3",
"glmnet.ext.5",
"glmnet.ext.10")
colnames(Resultados)=c("Size","Separation")
```

```
par(opar)
fit = smooth.spline(F.SS[!is.na(F.SS[,3]),1],F.SS[!is.na(F.SS[,3]),3],cv=TRUE)
fit.low = smooth.spline(F.SS[!is.na(F.SS[,3]),1],F.SS[!is.na(F.SS[,3]),4],cv=TRUE)
fit.up = smooth.spline(F.SS[!is.na(F.SS[,3]),1],F.SS[!is.na(F.SS[,3]),5],cv=TRUE)

plot(F.SS[,c(1,3)],pch=20,cex=0.25,col="darkgrey",xlab="n",ylab="Separation",main="Average separation for perfect classifiers",xlim=c(0,650),xaxp=c(0,650,13),ylim=c(0,max(Resultados[,2])+0.1))


lines(fit,col="black",lwd=1)
lines(fit.low,col="red",lwd=1)
lines(fit.up,col="red",lwd=1)
points(Resultados,col="blue",type="h")
points(Resultados,col="blue",pch=20)
text(Resultados[,1],Resultados[,2]+0.01,col="blue",labels=row.names(Resultados),cex=0.75)
```

We intersect the OTUs identified as significant according to LR and `coda_glmnet` with 3 replicates

```
Significant.capat=Significant[,c(1,2,3,4,5)]
Significant.capat=cbind(Significant.capat,Cuánto=rowSums(Significant.capat[,3:dim(Significant.capat)[2]]))
Significant.capat=Significant.capat[Significant.capat$Cuánto==2,]
```

```
Significant.capat[,1:2]%>%
  kbl() %>%
  kable_styling()
```

|  | OTUs | taxa |
| --- | --- | --- |
| OTU952 | OTU952 | d\_\_Bacteria;p\_\_Proteobacteria;c\_\_Gammaproteobacteria;o\_\_Beggiatoales;f\_\_Beggiatoaceae;g\_\_Thioflexothrix;s\_\_Thiotrichales\_bacterium |

A single OTU. The same happens with 5 replicates:

```
Significant.capat=Significant[,c(1,2,3,6,7)]
Significant.capat=cbind(Significant.capat,Cuánto=rowSums(Significant.capat[,3:dim(Significant.capat)[2]]))
Significant.capat=Significant.capat[Significant.capat$Cuánto==2,]

Significant.capat[,1:2]%>%
  kbl() %>%
  kable_styling()
```

|  | OTUs | taxa |
| --- | --- | --- |
| OTU828 | OTU828 | d\_\_Bacteria;p\_\_Verrucomicrobiota;c\_\_Verrucomicrobiae;o\_\_Verrucomicrobiales;f\_\_Rubritaleaceae;g\_\_Roseibacillus;s\_\_uncultured\_organism |

```
Resultados %>%
  kbl() %>%
  kable_styling()
```

|  | Size | Separation |
| --- | --- | --- |
| LR | 24 | 2.2969800 |
| glmnet.ext.3 | 45 | 0.9462113 |
| glmnet.ext.5 | 51 | 0.8765157 |
| glmnet.ext.10 | 49 | 0.8640208 |

### 5.6 CYM T0 vs CYM T1

```
tax_otu=as.data.frame(read_excel("TablaOTUs.xlsx"))[13:24,]

Samples=tax_otu$ID 
taxa=colnames(tax_otu[,3:dim(tax_otu)[2]])
OTUs=paste("OTU",1:(dim(tax_otu)[2]-2),sep="")

DF.0=tax_otu[,-c(1,2)]
row.names(DF.0)=Samples
colnames(DF.0)=OTUs

taxa.original=taxa
DF.0.original=DF.0
n.OTUs.original=dim(DF.0)[2]

Miseria=as.vector(which(colSums(DF.0[1:6,])<=3))
DF.0=DF.0[,-Miseria]
taxa=taxa[-Miseria]
OTUs=OTUs[-Miseria]

hit=function(x){min(c(x,1))}
DF.0.hits=as.matrix(DF.0)
DF.0.hits[]=vapply(DF.0.hits, hit, numeric(1))

Unics=data.frame(
Sample=rep(NA,length(which(colSums(DF.0.hits)==1))), OTUs=names(colSums(DF.0)[which(colSums(DF.0.hits)==1)]), Reads=colSums(DF.0)[which(colSums(DF.0.hits)==1)],
            Proportions=rep(0,length(which(colSums(DF.0.hits)==1)))  
)
for (i in 1:length(Unics$OTUs)){
ii=which(DF.0.hits[,Unics$OTUs[i]]==1)  
Unics$Proportions[i]=DF.0[ii,Unics$OTUs[i]]/sum(DF.0[ii,])
Unics$Sample[i]=Samples[ii]}
Unics.Freqs=Unics[Unics$Proportions>=0.05/100,]
row.names(Unics.Freqs)=NULL

DF.0=DF.0[,-which(colSums(DF.0.hits)==1)]
taxa=taxa[-which(colSums(DF.0.hits)==1)]
OTUs=OTUs[-which(colSums(DF.0.hits)==1)]

row.names(DF.0)=Samples

DF=zCompositions::cmultRepl(DF.0,method="GBM",output="p-counts",suppress.print=TRUE,z.warning=0.99)
```

```
DF=DF[1:6,]
DF.0=DF.0[1:6,]
DF.0.original=DF.0.original[1:6 ,]

Type=as.factor(c(rep("CYM_T0", 3),rep("CYM_T1", 3)))
colors=c("blue","green")[Type]
```

After removing OTUs with 2 or fewer reads across the samples and those appearing in only one of these samples, 1658 taxa remain.

The OTUs removed at this step that represent more than 0.05% of the sample in which they appear are:

```
Unics.Freqs[rev(order(Unics.Freqs$Proportions)),] %>%
  kbl() %>%
  kable_styling()
```

| Sample | OTUs | Reads | Proportions |
| --- | --- | --- | --- |
|  |  |  |  |

```
rm(tax_otu)
rm(DF.0.hits)
rm(Miseria)
rm(Unics.Freqs)
```

#### 5.6.1 How different are the samples?

```
HC=ClusterHC(DF,Grups=Type,colores=colors)
```

```
base=Sep(HC)
```

The two sample types are not initially separated.

We now examine the proportion of OTU subsets of reasonable size that classify them correctly.

```
> initial_seed=as.integer(Sys.time())
> print (initial_seed)
# [1] 1717668665
initial_seed %% 10000
# [1] 8665
```

```
set.seed(8665)
Experimento=function(i)
{
  ii=sample(dim(DF)[2],i,rep=FALSE)
  DFtemp=DF[,ii]
  CC=ClusterHC(DFtemp,dendrograma=FALSE,barplot=FALSE, Grups=Type,colores=colors)
  c(Encerts(CC$tabla,table(Type)),Sep(CC))
}
n=250
F.SS=c()
for (i in 10:650) #10
  {
  print(i)
  EE=replicate(n,Experimento(i))
  EE.2=EE[2,which(EE[1,]==0)]
  if(length(which(EE[1,]==0))>0){
XX=replicate(1000,mean(sample(EE.2,n,replace=TRUE)))
  F.SS=rbind(F.SS, c(i,
                     length(EE.2)/250,
                      mean(EE.2),
                      quantile(XX,0.025),
                      quantile(XX,0.975)
  ))}
    else {
    F.SS=rbind(F.SS, c(i,
                     0,
                      NA,
                      NA,
                      NA))
  }
  }
colnames(F.SS)=c("n","Proportion","Avg. sep.","Q_0.025","Q_0.975")
saveRDS(F.SS, file="Fraccio.Separadors.CYM.T0vsT1.RData")
```

```
F.SS=readRDS("Fraccio.Separadors.CYM.T0vsT1.RData")
```

The first plot shows, for each \(n\) up to 500, the proportion of samples of size \(n\) that perfectly classify CYM\_T0 and CYM\_T1 (black curve), along with the 95% Clopper–Pearson confidence interval for that proportion (red curves). We used *smoothing splines* to smooth the curves.

```
F.SS_prop.Q_0.025=epitools::binom.exact(F.SS[,2]*250,250)$lower
F.SS_prop.Q_0.975=epitools::binom.exact(F.SS[,2]*250,250)$upper
fit = smooth.spline(F.SS[,1],F.SS[,2],cv=TRUE)
fit.low = smooth.spline(F.SS[,1],F.SS_prop.Q_0.025,cv=TRUE)
fit.up = smooth.spline(F.SS[,1],F.SS_prop.Q_0.975,cv=TRUE)


plot(F.SS[,1:2],pch=20,cex=0.25,col="darkgrey",xlab="n",ylab="Proportion",main="Proportion of perfect classifiers",ylim=c(0,0.1),xlim=c(0,650),xaxp=c(0,650,13),yaxp=c(0,0.1,10))
lines(fit,col="black",lwd=1)
lines(fit.low,col="red",lwd=1)
lines(fit.up,col="red",lwd=1)
```

The second plot shows, for each \(n\) up to 600, the estimated mean separation obtained with a sample of size \(n\) that perfectly classifies CYM\_T0 and CYM\_T1 (black curve), along with the 95% confidence interval of this mean separation obtained via bootstrap (red curve).

```
fit = smooth.spline(F.SS[!is.na(F.SS[,3]),1],F.SS[!is.na(F.SS[,3]),3],cv=TRUE)
fit.low = smooth.spline(F.SS[!is.na(F.SS[,3]),1],F.SS[!is.na(F.SS[,3]),4],cv=TRUE)
fit.up = smooth.spline(F.SS[!is.na(F.SS[,3]),1],F.SS[!is.na(F.SS[,3]),5],cv=TRUE)

plot(F.SS[,c(1,3)],pch=20,cex=0.25,col="darkgrey",xlab="n",ylab="Separation",main="Average separation for perfect classifiers",xlim=c(0,650),xaxp=c(0,650,13),yaxp=c(0,1.5,15))
lines(fit,col="black",lwd=1)
lines(fit.low,col="red",lwd=1)
lines(fit.up,col="red",lwd=1)
```

`

#### 5.6.2 Taxa with maximum relevant log-ratios

We apply the LR approach exactly as described in the first comparison: OTUs are ranked by their log-ratio relevance, and we retain the subset maximizing the average absolute within-subset log-ratio association.

```
LRS=explore_logratios(DF,Type)
saveRDS(LRS, file="LRS_CYM_T0T1.RData")
```

```
LRS=readRDS("LRS_CYM_T0T1.RData")

assoc=rep(0,dim(DF)[2]-4)
for (m in 5:dim(DF)[2]){
    assoc[m-4]=sum(LRS$`association log-ratio with y`[1:m,1:m])/m^2
}

plot(5:dim(DF)[2],assoc,type="b",xlab="m",ylab="Average goodness of the m most relevant." ,pch=20,cex=0.5)
```

```
m=4+which.max(assoc)
impLR=sort(as.numeric(LRS$`order of importance`[1:m]))

HC=ClusterHC(DF[,impLR],barplot=FALSE,Grups=Type,colores=colors)
```

```
LR.max=c(length(impLR),Sep(HC))
```

The maximum is attained at the set of 169 most logratio-relevant taxa.  
They perfectly separate the two sample types. The separation between groups is 2.46.

```
Significant=data.frame(
  OTUs=paste("OTU",1:n.OTUs.original,sep=""),
  taxa=taxa.original,
  Log_ratio.max=Indicador(impLR))
```

#### 5.6.3 Taxa relevant for the bacterial signature (according to `coda_glmnet`)

As described above, `coda_glmnet` identifies a microbial signature by fitting a penalized log-contrast model and retaining OTUs with non-zero coefficients.

```
coda.glmnet=coda_glmnet(DF,Type,showPlots = FALSE)
```

```
## [1] "No variables are selected. The prediction and the signature plots are not displayed."
```

```
coda.glmnet$`signature plot`
```

```
## NULL
```

`coda_glmnet` does not find taxa with significant differences. We add random replicates. We test with 3, 5, and 10 replicates.

```
> initial_seed=as.integer(Sys.time())
> print (initial_seed)
# [1]  1717709647
initial_seed %% 10000
# [1]  9647
```

```
# 3 replicates
set.seed(9647)
M=3
repls=aldexCesc.clr(t(DF), conds=Type, mc.samples=M)@analysisData
```

```
## [1] "operating in serial mode"
```

```
repls=t(cbind(repls$CYM_T0_1,repls$CYM_T0_2,repls$CYM_T0_3,repls$CYM_T1_1,repls$CYM_T1_2,repls$CYM_T1_3))
attr(repls,"dimnames")[[2]]=NULL
colnames(repls)=colnames(DF)
row.names(repls)=c(paste("CYM_T0_1",1:M,sep="_"),
  paste("CYM_T0_2",1:M,sep="_"),
  paste("CYM_T0_3",1:M,sep="_"),
  paste("CYM_T1_1",1:M,sep="_"),
  paste("CYM_T1_2",1:M,sep="_"),
  paste("CYM_T1_3",1:M,sep="_"))
mostres=rbind(repls,easyCODA::CLR(DF)$LR)
rm(repls)
Type_ext=as.factor(c(rep("CYM_T0", 3*M),rep("CYM_T1", 3*M),rep("CYM_T0", 3),rep("CYM_T1", 3)))
colors_ext=c("blue","green")[Type_ext]
```

```
LogRat_mostres=logratios_matrix_clr(mostres)
LogRat_mostres=list(LogRat_mostres[[1]],LogRat_mostres[[2]])
saveRDS(LogRat_mostres, file="LogRat_mostres_CYM_T0T1_3copies.RData")
```

```
LogRat_mostres=readRDS("LogRat_mostres_CYM_T0T1_3copies.RData")
coda.glmnet.3=coda_glmnet_lr_bin(LogRat_mostres,Type_ext,DF,showPlots = FALSE)
```

```
impGLMNET_ext.3=coda.glmnet.3$taxa.num
HC=ClusterHC(DF[,impGLMNET_ext.3],dendrograma=TRUE,barplot=FALSE,Grups=Type,colores=colors)
```

```
glmnet.ext.3=c(length(impGLMNET_ext.3),Sep(HC))
```

With 3 replicates, 62 significant taxa are found, perfectly separating the two groups, with a separation of 2.05.

```
Significant=cbind(Significant,
glmnet_sign.ext.3.T0=Indicador(coda.glmnet.5$taxa.num[coda.glmnet.5$`log-contrast coefficients`>0]),
glmnet_sign.ext.3.T1=Indicador(coda.glmnet.5$taxa.num[coda.glmnet.5$`log-contrast coefficients`<0]))
```

```
# 5 replicates
set.seed(9647)
M=5
repls=aldexCesc.clr(t(DF), conds=Type, mc.samples=M)@analysisData
```

```
## [1] "operating in serial mode"
```

```
repls=t(cbind(repls$CYM_T0_1,repls$CYM_T0_2,repls$CYM_T0_3,repls$CYM_T1_1,repls$CYM_T1_2,repls$CYM_T1_3))
attr(repls,"dimnames")[[2]]=NULL
colnames(repls)=colnames(DF)
row.names(repls)=c(paste("CYM_T0_1",1:M,sep="_"),
  paste("CYM_T0_2",1:M,sep="_"),
  paste("CYM_T0_3",1:M,sep="_"),
  paste("CYM_T1_1",1:M,sep="_"),
  paste("CYM_T1_2",1:M,sep="_"),
  paste("CYM_T1_3",1:M,sep="_"))
mostres=rbind(repls,easyCODA::CLR(DF)$LR)
rm(repls)
Type_ext=as.factor(c(rep("CYM_T0", 3*M),rep("CYM_T1", 3*M),rep("CYM_T0", 3),rep("CYM_T1", 3)))
colors_ext=c("blue","green")[Type_ext]
```

```
LogRat_mostres=logratios_matrix_clr(mostres)
LogRat_mostres=list(LogRat_mostres[[1]],LogRat_mostres[[2]])
saveRDS(LogRat_mostres, file="LogRat_mostres_CYM_T0T1_5copies.RData")
```

```
LogRat_mostres=readRDS("LogRat_mostres_CYM_T0T1_5copies.RData")
coda.glmnet.5=coda_glmnet_lr_bin(LogRat_mostres,Type_ext,DF,showPlots = FALSE)
```

```
impGLMNET_ext.5=coda.glmnet.5$taxa.num
HC=ClusterHC(DF[,impGLMNET_ext.5],dendrograma=TRUE,barplot=FALSE,Grups=Type,colores=colors)
```

```
glmnet.ext.5=c(length(impGLMNET_ext.5),Sep(HC))
```

With 5 replicates, 74 significant taxa are found, perfectly separating the two groups, with a separation of 1.36.

```
Significant=cbind(Significant,
glmnet_sign.ext.5.T0=Indicador(coda.glmnet.5$taxa.num[coda.glmnet.5$`log-contrast coefficients`>0]),
glmnet_sign.ext.5.T1=Indicador(coda.glmnet.5$taxa.num[coda.glmnet.5$`log-contrast coefficients`<0]))
```

```
# 10 replicates
set.seed(9647)
M=10
repls=aldexCesc.clr(t(DF), conds=Type, mc.samples=M)@analysisData
```

```
## [1] "operating in serial mode"
```

```
repls=t(cbind(repls$CYM_T0_1,repls$CYM_T0_2,repls$CYM_T0_3,repls$CYM_T1_1,repls$CYM_T1_2,repls$CYM_T1_3))
attr(repls,"dimnames")[[2]]=NULL
colnames(repls)=colnames(DF)
row.names(repls)=c(paste("CYM_T0_1",1:M,sep="_"),
  paste("CYM_T0_2",1:M,sep="_"),
  paste("CYM_T0_3",1:M,sep="_"),
  paste("CYM_T1_1",1:M,sep="_"),
  paste("CYM_T1_2",1:M,sep="_"),
  paste("CYM_T1_3",1:M,sep="_"))
mostres=rbind(repls,easyCODA::CLR(DF)$LR)
rm(repls)
Type_ext=as.factor(c(rep("CYM_T0", 3*M),rep("CYM_T1", 3*M),rep("CYM_T0", 3),rep("CYM_T1", 3)))
colors_ext=c("blue","green")[Type_ext]
```

```
LogRat_mostres=logratios_matrix_clr(mostres)
LogRat_mostres=list(LogRat_mostres[[1]],LogRat_mostres[[2]])
saveRDS(LogRat_mostres, file="LogRat_mostres_CYM_T0T1_10copies.RData")
```

```
LogRat_mostres=readRDS("LogRat_mostres_CYM_T0T1_10copies.RData")
coda.glmnet.10=coda_glmnet_lr_bin(LogRat_mostres,Type_ext,DF,showPlots = FALSE)
```

```
impGLMNET_ext.10=coda.glmnet.10$taxa.num
HC=ClusterHC(DF[,impGLMNET_ext.10],dendrograma=TRUE,barplot=FALSE,Grups=Type,colores=colors)
```

```
glmnet.ext.10=c(length(impGLMNET_ext.10),Sep(HC))
```

With 10 replicates, 67 significant taxa are found, perfectly separating the two groups, with a separation of 1.26.

```
Significant=cbind(Significant,
glmnet_sign.ext.10.T0=Indicador(coda.glmnet.10$taxa.num[coda.glmnet.10$`log-contrast coefficients`>0]),
glmnet_sign.ext.10.T1=Indicador(coda.glmnet.10$taxa.num[coda.glmnet.10$`log-contrast coefficients`<0]))
```

#### 5.6.4 OTUs significant according to ALDeX

```
> initial_seed=as.integer(Sys.time())
> print (initial_seed)
# [1] 1717738742
initial_seed %% 10000
# [1] 8742
```

```
set.seed(8742)
repl_kw=aldexCesc.clr(t(DF), conds=Type, mc.samples=1000, verbose=FALSE)
aldex_kw=aldex.kw(repl_kw, verbose=FALSE)
#
repl_glm=aldexCesc.clr(t(DF), conds=model.matrix(~Type,data.frame(Type)), mc.samples=1000, verbose=FALSE)
aldex_glm=aldex.glm(repl_glm, model.matrix(~Type,data.frame(Type)))
#'
saveRDS(aldex_kw, file="aldex_kw_CYM_T0T1.RData")
saveRDS(aldex_glm, file="aldex_glm_CYM_T0T1.RData")
```

```
p.valores_kw=readRDS("aldex_kw_CYM_T0T1.RData")
```

```
length(p.valores_kw[p.valores_kw[,2]<0.05,2])
```

```
## [1] 0
```

```
length(p.valores_kw[p.valores_kw[,4]<0.05,2])
```

```
## [1] 88
```

```
length(p.valores_kw[p.valores_kw[,4]<0.01,4])
```

```
## [1] 6
```

There are no OTUs with an adjusted Kruskal–Wallis p-value < 0.05. There are 66 OTUs with an unadjusted p-value < 0.05, but they do not separate the samples well:

```
sep.kw.noadjust.0.05=QQ.HC.noadjust(p.valores_kw,DF,Type,q=0.05,1,BP=FALSE)
```

```
Significant=cbind(Significant,
Aldex.kw.noadjust.0.05=Indicador(sep.kw.noadjust.0.05))
```

#### 5.6.5 Simper

The significant taxa identified by `simper` do not classify the samples well:

```
DF.prop=DF.0.original/rowSums(DF.0.original)
SMP.prop=simper(DF.prop,group=Type)
indexos_SMP=SMP.prop$CYM_T0_CYM_T1$ord[which(SMP.prop$CYM_T0_CYM_T1$cusum<=0.7)]
OTUs_SMP=sort(attr(SMP.prop$CYM_T0_CYM_T1$cusum[which(SMP.prop$CYM_T0_CYM_T1$cusum<=0.7)],"names"))
OTUs_SMP.good=setdiff(OTUs_SMP,Unics$OTUs)
HC=ClusterHC(DF[,OTUs_SMP.good],dendrograma=TRUE,barplot=FALSE,Grups=Type,colores=colors)
```

```
Significant=cbind(Significant,
t(DF.prop))
```

#### 5.6.6 Summary

```
write.csv2(Significant,"OTU_Significativos_CYM_T0T1.csv",row.names=FALSE)
```

```
Resultados=rbind(
LR.max,
glmnet.ext.3,
glmnet.ext.5,
glmnet.ext.10)
row.names(Resultados)=c(
"LR",
"glmnet.ext.3",
"glmnet.ext.5",
"glmnet.ext.10"
)
colnames(Resultados)=c("Size","Separation")
```

```
par(opar)
fit = smooth.spline(F.SS[!is.na(F.SS[,3]),1],F.SS[!is.na(F.SS[,3]),3],cv=TRUE)
fit.low = smooth.spline(F.SS[!is.na(F.SS[,3]),1],F.SS[!is.na(F.SS[,3]),4],cv=TRUE)
fit.up = smooth.spline(F.SS[!is.na(F.SS[,3]),1],F.SS[!is.na(F.SS[,3]),5],cv=TRUE)

plot(F.SS[,c(1,3)],pch=20,cex=0.25,col="darkgrey",xlab="n",ylab="Separation",main="Average separation for perfect classifiers",xlim=c(0,650),xaxp=c(0,650,13),ylim=c(0,max(Resultados[,2])+0.1))


lines(fit,col="black",lwd=1)
lines(fit.low,col="red",lwd=1)
lines(fit.up,col="red",lwd=1)
points(Resultados,col="blue",type="h")
points(Resultados,col="blue",pch=20)
text(Resultados[,1],Resultados[,2]+0.01,col="blue",labels=row.names(Resultados),cex=0.75)
```

We intersect the OTUs identified as significant according to LR and `coda_glmnet` with 3 replicates.

```
Significant.capat=Significant[,c(1,2,3,4,5)]
Significant.capat=cbind(Significant.capat,Cuánto=rowSums(Significant.capat[,3:dim(Significant.capat)[2]]))
Significant.capat=Significant.capat[Significant.capat$Cuánto==2,]
```

```
DF.Imp=DF[,Significant.capat[,1]]
HC=ClusterHC(DF.Imp,dendrograma=TRUE,barplot=FALSE,Grups=Type,colores=colors)
```

There are 1 taxa. They are:

```
Significant.capat[,1:2]%>%
  kbl() %>%
  kable_styling()
```

|  | OTUs | taxa |
| --- | --- | --- |
| OTU235 | OTU235 | d\_\_Bacteria;p\_\_Proteobacteria;c\_\_Gammaproteobacteria;o\_\_Thiomicrospirales;f\_\_Thiomicrospiraceae;g\_\_endosymbionts;s\_\_uncultured\_bacterium |

The separation between groups is 0.98.

We update the plot with the separations:

```
intersección=c(dim(Significant.capat)[1],Sep(HC))
Resultados=rbind(Resultados,intersección)
row.names(Resultados)=c(
"LR",
"glmnet.ext.3",
"glmnet.ext.5",
"glmnet.ext.10",
"intersection")
colnames(Resultados)=c("Size","Separation")
```

```
par(opar)
fit = smooth.spline(F.SS[!is.na(F.SS[,3]),1],F.SS[!is.na(F.SS[,3]),3],cv=TRUE)
fit.low = smooth.spline(F.SS[!is.na(F.SS[,3]),1],F.SS[!is.na(F.SS[,3]),4],cv=TRUE)
fit.up = smooth.spline(F.SS[!is.na(F.SS[,3]),1],F.SS[!is.na(F.SS[,3]),5],cv=TRUE)

plot(F.SS[,c(1,3)],pch=20,cex=0.25,col="darkgrey",xlab="n",ylab="Separation",main="Average separation for perfect classifiers",ylim=c(0,2.25),xlim=c(0,250),xaxp=c(0,250,10),yaxp=c(0,2.5,10))
lines(fit,col="black",lwd=1)
lines(fit.low,col="red",lwd=1)
lines(fit.up,col="red",lwd=1)
points(Resultados,col="blue",type="h")
points(Resultados,col="blue",pch=20)
text(Resultados[,1],Resultados[,2]+0.02,col="blue",labels=row.names(Resultados),cex=0.75)
abline(h=2.47,col="green")
text(20,2.6,col="green",labels="Global",cex=0.75)
```

```
Resultados %>%
  kbl() %>%
  kable_styling()
```
